# Microbial Transformation of Yakuchinone A and Cytotoxicity Evaluation of Its Metabolites

**DOI:** 10.3390/ijms23073992

**Published:** 2022-04-03

**Authors:** Chen Huo, Fubo Han, Yina Xiao, Hyun Jung Kim, Ik-Soo Lee

**Affiliations:** 1 College of Pharmacy, Chonnam National University, Gwangju 61186, Korea; huochen_0213@163.com (C.H.); hanfubo0306@gmail.com (F.H.); yogurtxiao@163.com (Y.X.); 2 College of Pharmacy and Natural Medicine Research Institute, Mokpo National University, Muan-gun 58554, Jeonnam, Korea

**Keywords:** yakuchinone A, microbial transformation, cytotoxicity, Mosher’s method

## Abstract

Yakuchinone A (**1**) is a bioactive diarylheptanoid isolated from the dried fruits of *Alpinia oxyphylla.* Microbial transformation has been recognized as an efficient method to produce new biologically active derivatives from natural products. In the present study, microbial transformation of yakuchinone A was performed with the fungus *Mucor hiemalis* KCTC 26779, which led to the isolation of nine new metabolites (**2**, **3a**, **3b**, and **4**–**9**). Their structures were elucidated as (3*S*)-oxyphyllacinol (**2**), (3*S,7R*)- and (3*S,7S*)-7-hydroxyoxyphyllacinol (**3a** and **3b**), (3*S*)-oxyphyllacinol-4′-*O*-β-d-glucopyranoside (**4**), (3*S*)-4″-hydroxyoxyphyllacinol (**5**), (3*S*)-3″-hydroxyoxyphyllacinol (**6**), (3*S*)-2″-hydroxyoxyphyllacinol (**7**), (3*S*)-2″-hydroxyoxyphyllacinol-2″-*O*-β-d-glucopyranoside (**8**), and (3*S*)-oxyphyllacinol-3-*O*-β-d-glucopyranoside (**9**) based on the comprehensive spectroscopic analyses and the application of modified Mosher’s method. All compounds were evaluated for their cytotoxic activities against melanoma, as well as breast, lung, and colorectal cancer cell lines. Compound **9**, which was *O*-glucosylated on the diarylheptanoid alkyl chain, exhibited the most selective cytotoxic activities against melanoma cell lines with the IC_50_ values ranging from 6.09 to 9.74 μM, indicating that it might be considered as a possible anti-cancer lead compound.

## 1. Introduction

*Alpinia oxyphylla* Miq. (Zingiberaceae) is a medicinal plant widely distributed in the subtropical parts of Asia. It has been used as traditional medicine for the treatment of nephrotic syndrome, ulceration, dementia, and intestinal disorders for more than a thousand years [1]. Pharmacological and phytochemical investigations of *A. oxyphylla* have led to the identification of a variety of biologically active components including fruit oil, flavonoids, sesquiterpenes, diarylheptanoids, and phenolic compounds [2,3]. Among them, diarylheptanoids have been identified as a major class of characteristic constituents in *A. oxyphylla* with diverse biological effects including anti-inflammatory, antioxidant, anti-hepatotoxic, antitumor, and anti-proliferative activities [4,5]. Especially, yakuchinone A (**1**), a major pungent ingredient of *A. oxyphylla*, has been reported to exhibit antioxidant, anti-inflammatory, antibacterial, antimutagenic, and gastroprotective activities [6,7,8]. It was also reported to show a strong inhibitory effect against prostaglandin synthase, which was considered to be associated with anti-platelet aggregation activity [9]. Moreover, the skin carcinogenesis and tumor promoter-induced inflammation in mice have been suppressed after treatment with **1** [10]. Compound **1** has been shown to induce apoptotic death in HL-60 cells and appeared to be relatively less cytotoxic to normal cells [11], indicating the potential of **1** as an effective agent for cancer chemoprevention. Considering the potential biological effects of **1**, it is meaningful to expand its structural diversity and produce more derivatives with enhanced bioavailability and pharmacological activities.

Microbial transformation has been regarded as an efficient and green method for the conversion of bioactive compounds to structurally related derivatives with potential biological properties [12,13]. The transformation processes are facilitated by the enzymes produced by the microbial strains. Microbial transformation is usually used as an alternative method to produce bioactive compounds which are difficult to prepare by chemical approaches. These transformations are carried out under multiple enzymatic reactions including oxidation, reduction, hydroxylation, glycosylation, and isomerization [14]. Compared to chemical synthesis, microbial transformation exhibits higher enantio- and regioselectivity in aqueous solutions, and it can be performed under milder conditions without protection and deprotection of functional groups [15,16].

To identify bioactive derivatives of **1** and investigate their structure-activity relationships (SAR), microbial transformation of **1** was conducted using the selected fungus *Mucor hiemalis*, which resulted in the isolation of nine previously undescribed metabolites (**2**, **3a**, **3b**, and **4**–**9**). The structures of all the compounds were characterized by spectroscopic methods, and their cytotoxic activities were evaluated against B16F1, B16F10, A375P, A549, MCF-7, and HT-29 cancer cell lines.

## 2. Results and Discussion

**Figure 1:**
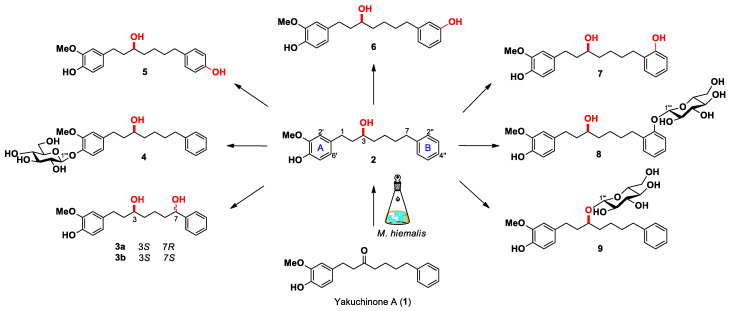
Compounds **2**, **3a**, **3b**, and **4**–**9** obtained from microbial transformation of **1** with *M. hiemalis*.

A total of 22 microbial strains were screened to identify microorganisms capable of metabolizing **1** (Supplementary Materials Table S1). TLC and HPLC analyses of the screening results revealed that *Mucor hiemalis* KCTC 26779 exhibited the highest transformation capability and thus selected for the scale-up studies. Subsequent preparative scale fermentation of **1** was successfully carried out with *M. hiemalis*, which led to the production of nine previously undescribed metabolites (**2**, **3a**, **3b**, and **4**–**9**, Figure 1).

### 2.1. Structure Elucidation

**Figure 2:**
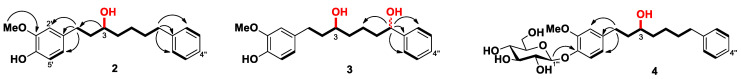
Selected HMBC (^1^H→^13^C) and COSY (^1^H-^1^H) correlations of compounds **2**, **3,** and **4**.

Compound **2**, obtained as a colorless oil, had a molecular formula of C_20_H_26_O_3_ which was established on the basis of its HRFDMS spectrum. The ^1^H NMR spectrum of **2** revealed two substituted benzene rings, in which the resonances at δ_H_ 6.75 (1H, d, *J* = 1.8 Hz, H-2′), 6.69 (1H, d, *J* = 8.0 Hz, H-5′), and 6.51 (1H, dd, *J* = 8.0, 1.8 Hz, H-6′) indicated a 1,3,4-trisubstituted benzene ring, and the resonances at δ_H_ 7.23 (2H, H-3″/H-5″), and 7.14 (3H, H-2″/H-4″/H-6″) suggested a monosubstituted benzene ring. Its ^13^C NMR data displayed twenty carbon signals, which were classified as one methoxy group (3′-OCH_3_), six methylenes, one oxygenated methine, and twelve aromatic carbons with the help of its HSQC spectrum. The NMR data of **2** were similar to those of parent compound **1**, except for the appearance of the proton signal at δ_H_ 3.51 corresponding to the carbon signal at δ_C_ 71.8 and absence of the carbonyl signal at δ_C_ 212.0 (C-3), indicating that a hydroxyl group was attached at C-3 position in **2**. In addition, the attachment of hydroxyl group at C-3 position was further confirmed by the correlations from H-1 to C-3/1′/2′/6′, H-2 to C-1′, and H-3 to C-1 in its HMBC spectrum (Figure 2). The locations of rings A and B were, respectively, assigned at C-1 and C-7 by the HMBC correlations from H-1 to C-1′/2′/6′, and from H-7 to C-1″/2″/6″. Detailed analysis of its 2D NMR data not only supported the above mentioned inferences but also verified the assignments of **2** (Supporting Figures S8–S10). The structure of **2** was identical to that of oxyphyllacinol whose absolute configuration was not assigned [17]. The absolute configuration of **2** at C-3 was determined using the advanced Mosher’s method by spectral analyses of its (*R*)- and (*S*)-MTPA ester derivatives [18,19,20]. The proton signals of the two esters were assigned based on the correlations shown in their ^1^H-^1^H COSY spectra, and the positive value of *Δ*δ_H_ (= δ*_S_*−δ*_R_*) at H-2 and the negative value of *Δ*δ_H_ at H-4 suggested the *S* configuration at C-3 (Supplementary Materials Figure S1). Finally, the structure of **2** was elucidated as (3*S*)-oxyphyllacinol. 

Compound **3** was obtained as a yellow oil. It showed a molecular ion [M]^+^ peak at *m/z* 330.1830 (calcd *m/z* 330.1826) in the HRFDMS spectrum, corresponding to a molecular formula of C_20_H_26_O_4_. Analysis of 1D and 2D NMR data indicated that **3** contained the same benzene ring systems as those of **2** (Supplementary Materials Figures S13–S18). In addition, its 1D NMR data showed one new proton signal at δ_H_ 4.48 corresponding to the new oxygenated methine carbon signal at δ_C_ 72.8, indicating that **3** was a hydroxylated derivative of **2**. The proton signal at δ_H_ 4.48 in **3** was significantly downfield-shifted from δ_H_ 2.60 (H-7) in **2**, suggesting that the hydroxyl group was located at C-7. Moreover, the location was further confirmed by the long-range correlations from H-7 (δ_H_ 4.48) to C-1″ (δ_C_ 146.5)/C-2″/6″ (125.8) in the HMBC spectrum of **3** (Figure 2). Complete assignment of the proton and carbon signals of **3** was accomplished through the full interpretation of 1D and 2D NMR spectra.

**Figure 3:**
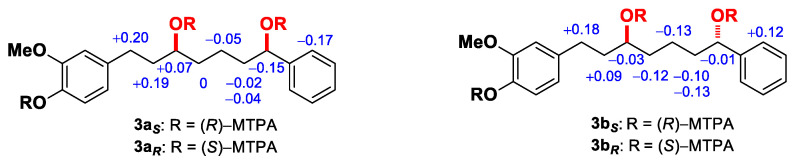
*Δδ*_H_ (= δ*_S_* − δ*_R_*) values for the Mosher ester derivatives of **3a** and **3b**.

Furthermore, compound **3** was identified as a diastereomeric mixture by HPLC using a YMC Amylose-SA chiral column (250 × 4.6 mm, 5 μm), which was effectively separated into **3a** and **3b**. The Mosher ester analysis was performed to determine the absolute configurations of the diol containing two secondary hydroxyl groups at C-3 and C-7. The ^1^H NMR data for bis-MTPA derivatives were assigned by analysis of COSY and NOE spectra and the calculated *Δ*δ values (δ_H_ = δ_S_−δ_R_, ppm) are shown in Figure 3. The absolute configurations at C-3 and C-7 in **3a** were assigned *S* and *R*, respectively, according to the *Δ*δ values, which are consistent with the *Δ*δ_SR_ sign distribution proposed by Riguera and colleagues for the acyclic 1,5-diol systems [21,22,23,24]. Similarly, the absolute configurations at C-3 and C-7 in **3b** were determined to be *S* and *S*, respectively. Consequently, **3a** was assigned (3*S,7R*)-7-hydroxyoxyphyllacinol, and **3b** (3*S,7S*)-7-hydroxyoxyphyllacinol.

The molecular formula of compound **4** was determined as C_26_H_36_O_8_ by the molecular ion [M]^+^ peak at *m/z* 476.2413 (calcd *m/z* 476.2405) based on the HRFDMS data, corresponding to the presence of an additional glucose unit (C_6_H_10_O_5_) compared with that of **2**. In the ^1^H NMR spectrum of **4**, seven new typical characteristic signals between δ_H_ 3.46 and 4.83 owing to s sugar moiety were observed. The ^13^C NMR spectrum of **4** exhibited six new carbon signals at δ_C_ 101.7, 76.7, 76.4, 73.5, 69.9, and 61.1, which are consistent with the characteristics of a glucose moiety [25,26,27]. Meanwhile, the anomeric proton signal at δ_H_ 4.83 (H-1‴) with a large coupling constant (*J* = 7.7 Hz) indicated that the glycosidic bond is in β-form. In addition, the aromatic proton signal of **4** at δ_H_ 7.08 (H-5′) was significantly downfield-shifted compared with that of **2** (Table 1), suggesting that the glucose moiety was located at C-4′ position through an ether linkage. The connection between the hydroxyl group at C-4′ and the glucose moiety was further confirmed by the HMBC correlation from H-1‴ to C-4′ (Figure 2). Ultimately, compound **4** was elucidated as (3*S*)-oxyphyllacinol-4′-*O*-β-d-glucopyranoside.

**Figure 4:**
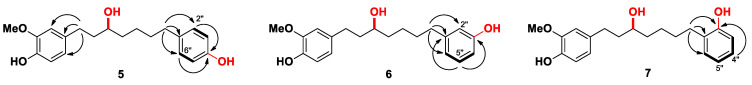
Selected HMBC (^1^H→^13^C) and COSY (^1^H-^1^H) correlations of compounds **5**, **6**, and **7**.

Compound **5** had the molecular formula of C_20_H_26_O_4_ as deduced by its HRFDMS data, which was 16 units higher than that of **2**, suggesting that **5** was a hydroxylated derivative of **2**. The ^1^H data of **5** closely resembled those of **2**, except for the presence of *p*-substituted benzene signals at δ_H_ 6.97 (2H, d, *J* = 8.1 Hz, H-2″/6″), and 6.67 (2H, d, *J* = 8.1 Hz, H-3″/5″) instead of the signals for the monosubstituted ring B in **2**, indicating that the hydroxyl group was substituted at C-4″ position. Additionally, the symmetric carbon signals of ring B provided further evidence for the *para*-substituted hydroxyl group that existed in **5**. The HMBC cross-peaks from H-2″/6″ (δ_H_ 6.97) to C-4″ (δ_C_ 156.4) further confirmed the hydroxyl group was located at C-4″ (Figure 4). The absolute configuration at C-3 in **5** was assigned *S* according to the *Δ*δ values calculated for the *S*- and *R*-MTPA esters (Supplementary Materials Figure S1). Accordingly, the structure of **5** was elucidated as (3*S*)-4″-hydroxyoxyphyllacinol.

Compound **6** was assigned the same molecular formula of C_20_H_26_O_4_ as **5** from its HRFDMS data at *m/z* 330.1832 (calcd for 330.1826). Analysis of its NMR spectra indicated that **6** was a regioisomer of **5**, with a hydroxyl group at different location in ring B (Table 2). The resonances at δ_H_ 7.05 (1H, t, *J* = 7.7 Hz, H-5″), and δ_H_ 6.63 (3H, H-2″/H-4″/H-6″) revealed that the hydroxyl group was located at C-3″ in **6**. The HMBC correlation from H-5″ (δ_H_ 7.05) to the hydroxylated aromatic carbon C-3″ (δ_C_ 158.4) further confirmed that the hydroxyl group was introduced at *meta* position of ring B in **6**. The absolute configuration of **6** was determined as 3*S* according to the *Δ*δ values calculated for the *S*- and *R*-MTPA esters (Supplementary Materials Figure S1). Hence, the structure of **6** was elucidated as (3*S*)-3″-hydroxyoxyphyllacinol.

Compound **7** displayed the same molecular formula of C_20_H_26_O_4_ as those of **5** and **6**, according to its HRFDMS data at *m/z* 330.1832 [M]^+^ (calcd *m/z* 330.1826). Interpretation of the ^1^H and ^13^C NMR data in combination with 2D NMR (COSY, HSQC, and HMBC) spectra indicated that **7** was also a regioisomer of **5**. The *ortho*-hydroxylated ring B system was identified by the sequential COSY correlations of H-3″, H-4″, H-5″ and H-6″. Its HMBC correlations from H-7 (δ_H_ 2.58) to C-2″ (δ_C_ 156.4)/ C-6″ (δ_C_ 131.2) provided further evidence for the *ortho*-hydroxylation in ring B. The absolute configuration of **7** was determined as 3*S* based on the Mosher’s method (Supplementary Materials Figure S1). Therefore, the entire structure of **7** was established as (3*S*)-2″-hydroxyoxyphyllacinol.

**Figure 5:**
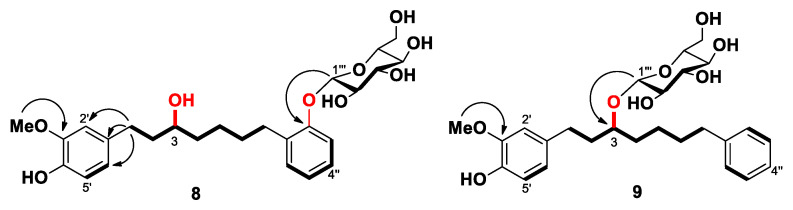
Selected HMBC (^1^H→^13^C) and COSY (^1^H-^1^H) correlations of compounds **8** and **9**.

Compound **8** was found to possess a molecular formula of C_26_H_36_O_9_ on the basis of its HRFDMS peak at *m/z* 492.2370 (calcd *m/z* 492.2354). The ^1^H and ^13^C NMR spectra of **8** showed seven and six additional resonance signals, respectively, which are characteristic of a glucose moiety. Appearance of the anomeric proton signal at δ_H_ 4.89 (H-1‴) with a larger coupling constant (7.5 Hz) suggested that the glucose moiety has a β-configuration. Additionally, the ^1^H NMR data of the aglycone of **8** were similar to those of **7**. However, the proton signal at δ_H_ 7.11 (2H, H-3″/H-5″) in **8** was significantly downfield-shifted compared with the signal at δ_H_ 6.71 (2H, H-3″/H-5″) in **7**, which indicated that the glucose moiety was connected to the hydroxyl group at C-2″. The correlation from H-1‴ to C-2″ (δ_C_ 157.1) in the HMBC spectrum of **8** further confirmed that the glucose moiety was located at C-2″ (Figure 5). Therefore, the structure of **8** was elucidated as (3*S*)-2″-hydroxyoxyphyllacinol-2″-*O*-β-d-glucopyranoside.

Compound **9** was obtained as a yellow oil. Its molecular formula of C_26_H_36_O_8_ was supported by the HRFDMS data, indicating that it was a glucosylated derivative of **2**. In the ^1^H and ^13^C NMR spectra of **9**, the proton signals at δ_H_ 4.30 and 3.19-3.69 (6H) and carbon signals at δ_C_ 104.0, 78.4, 77.8, 75.5, 71.9, and 63.0 also established the presence of a glucose moiety. The glucose was determined to be in β-configuration by the large coupling constant (7.8 Hz) of the anomeric proton signal at δ_H_ 4.30. In comparison of its ^1^H NMR data with those of **2**, the significantly downfield-shifted signals of H-3 (δ_H_ 3.69), and C-3 (δ_C_ 80.3) in **9** indicated that the sugar moiety was attached to the hydroxyl group at C-3 (Table 1 and Table 2). In addition, HMBC correlation of H-1‴ to C-3 confirmed that the glucose moiety was connected to the aglycone at C-3 in **9** (Figure 5). Based on the above analyses, the structure of **9** was elucidated as (3*S*)-oxyphyllacinol-3-*O*-β-d-glucopyranoside.

### 2.2. Cytotoxicity Evaluation

Cytotoxic activities of all the compounds (**1**-**2**, **3a**, **3b**, **4**–**9**) were examined against two murine cancer cell lines (B16F1 and B16F10), and four human cancer cell lines (A375P, A549, MCF-7, and HT-29) using the modified MTT assay, and their IC_50_ values are summarized in Table 3. Most of the compounds showed more potent cytotoxic activities against melanoma cell lines than against lung, breast, and colorectal cancer cell lines. Compound **9** exhibited the strongest selective cytotoxic activities against melanoma cell lines with IC_50_ values ranging from 6.09 to 9.74 μM.

### 2.3. Discussion

Biotransformation is currently recognized as an efficient and environmentally-friendly technology for asymmetric synthesis with the high enantioselectivity [28,29]. It has been reported that fungi can be considered as a promising source of new biocatalysts, especially for chiral reactions [30]. Additionally, *Mucor hiemalis* was found to exhibit good catalytic activity in the reduction of carbonyl compounds and show high stereoselectivity [31]. In this experiment, the ketone at C-3 was stereoselectively reduced into the corresponding (*S*)-configured alcohol by this fungus, while hydroxylation at C-7 position at the aliphatic fragment proceeded regioselectively.

In the transformation process, **2** was thought to be formed by reduction of the carbonyl group in **1**, and it is regarded as a precursor of the compounds **3a**, **3b**, and **4**–**9**, which were produced through hydroxylation and glucosylation reactions. The methylene group at C-7 position in **2** was directly hydroxylated to form the corresponding alcohol group in metabolite **3**. Similarly, the phenolic metabolites **5**, **6** and **7** were directly converted from **2** via aromatic hydroxylation at the *para* (C-4″), *meta* (C-3″), and *ortho* (C-2″) positions in ring B, respectively. Further *O*-glucosylation at C-2″ in **7** led to the formation of metabolite **8**, and similar modifications apparent at the C-4′ and C-3 positions in **2** led to the formation of **4** and **9**, respectively. Noteworthily, it has been reported that hydroxylation of a mono-alkylated benzene ring usually takes place at *para* or *ortho* position, while, the *meta*-hydroxylation rarely occurs in nature [32]. In the present study, the yield of *meta*-hydroxylated metabolite **6** was quite low compared with that of *para*- and *ortho*-hydroxylated metabolites (**5** and **7**), suggesting that microbial transformation can be used to mimic the natural biosynthetic pathways for production of secondary metabolites [33].

SAR analysis could lead us to comprehend the compound characteristics and design more potent therapeutic agents in the future [34]. In this study, it is clear that all the active metabolites showed higher cytotoxic activity against melanoma cell lines than the other three cancer cell lines. Compound **1** showed potent cytotoxic activities against all six cancer cell lines. However, compound **2** showed lower cytotoxic activities than **1**, indicating that the carbonyl group could be considered to play a significant role in biological activities of diarylheptanoids. In addition, the role of phenolic hydroxyl group in anti-melanoma activities seemed to be substantial due to the result that compounds **5**–**7** with an additional phenolic hydroxyl group exhibited stronger activity than compound **2**. In addition, this was supported by the results observed by Ali and colleagues [35]. Meanwhile, compound **5** bearing a *para*-hydroxyl group in ring B showed greater activities against melanoma cell lines than the other two regioisomers. Furthermore, loss of activity was observed for compound **3**, suggesting that the addition of a hydroxyl group on the alkyl chain could decrease its cytotoxic activity. On the other hand, among the three glucosylated metabolites (**4**, **8**, and **9**), compound **9** displayed the most potent and selective cytotoxic activities against melanoma cell lines, even stronger than its parent compounds **1** and **2**, suggesting that glucosylation on the long alkyl chain of diarylheptanoids could improve their bioactivities.

## 3. Materials and Methods

### 3.1. General Experimental Procedures

UV spectra were taken with a Jasco V-530 spectrophotometer (Jasco, Tokyo, Japan), and IR spectra were obtained on a PerkinElmer Spectrum 400 FT-IR/NIR spectrometer (Waltham, MA, USA). Optical rotations were measured on a PerkinElmer 343 Plus polarimeter (Waltham, MA, USA). HRFDMS were measured on a JMS-T200GC AccuTOF™ GCx-plus High Performance Gas Chromatograph-Time-of-Flight Mass Spectrometer (Jeol Ltd., Tokyo, Japan). NMR experiments were performed on a Varian Unity Inova 500 spectrometer (Varian Inc., Palo Alto, CA, USA) or a Bruker Avance III HD 400 spectrometer (Bruker, Billerica, MA, USA) using DMSO-*d_6_* and CD_3_OD as solvents and TMS as internal standard. Chemical shift values (δ) were reported in parts per million (ppm), and the coupling constants (*J*) were reported in hertz (Hz). Thin-layer chromatography (TLC) was carried out on precoated silica gel 60 F_254_ plates (Merck, Darmstadt, Germany). Reversed-phase HPLC was performed on a Waters 1525 Binary HPLC pump equipped with a 996 photodiode array (PDA) detector using a Phenomenex Luna C18 (250 × 10 mm, 5 μm) column or a Zorbax Rx C8 (150 × 4.6 mm, 5 μm) with MeOH-H_2_O at a flow rate of 2.0 mL/min or 1.0 mL/min, respectively. Separation of diastereomers was carried out on a YMC Amylose-SA chiral column (250 × 4.6 mm, 5 μm) with hexane-ethanol at a flow rate of 1.0 mL/min.

### 3.2. Chemicals and Ingredients

The fruits of *A. oxyphylla* were purchased from Sehwadang (Gwangju, Korea), which were identified by DaeHyo Pharmacy Co., Ltd. (Suwon, Korea), and a voucher specimen (SHD-B-230-161101) has been deposited at the College of Pharmacy, Chonnam National University. The powdered *A. oxyphylla* was extracted with methanol (9 L × 3) under ultrasonication (40 kHz), the suspension was filtered and then concentrated by rotary evaporation to obtain crude extract. The resulting residue was dissolved in water and partitioned with *n*-hexane, CH_2_Cl_2_, EtOAc, and BuOH. The CH_2_Cl_2_ extract was subjected to silica gel column, eluting with a gradient solvent system of CH_2_Cl_2_-MeOH (100:0→0:100) to give nine fractions. Briefly, compound **1** was isolated from Fr.2 and was identified as yakuchinone A by comparison of its NMR data (Supplementary Materials Figures S2–S6) with previously reported data [36]. Ingredients for microbial media including potato dextrose broth, D-glucose, peptone, malt extract, and yeast extract were purchased from Becton, Dickinson and Company (Sparks, MD, USA). 5-Fluorouracil (5-FU) and demethylzeylasteral (DZ) used as controls in the bioassay were purchased from Tokyo Chemical Industry Co., Ltd. (Tokyo, Japan) and Biopurify Phytochemicals Ltd. (Chengdu, China), respectively. Phosphate buffered saline (PBS) tablets were purchased from Takara Korea Biomedical Inc. (Seoul, Korea), and fetal bovine serum (FBS) was from Welgene Inc. (Gyeongsan-si, Korea). Antibiotic-Antimycotic and Dulbecco’s Modified Eagle Medium (DMEM) were purchased from Gibco (Invitrogen, Carlsbad, CA, USA). Thiazolyl blue tetrazolium bromide (MTT) was purchased from Thermo Fisher Scientific (Waltham, MA, USA).

### 3.3. Fungal Strains and Culture Media

All the microorganisms used in the preliminary screening were obtained from the Korean Collection for Type Cultures (KCTC, Jeongeup, Korea) and the Korean Culture Center of Microorganisms (KCCM, Seoul, Korea). Twenty-two cultures were used for screening as follows: *Absidia coerulea* KCTC 6936, *Alternaria alternata* 6005, *Aspergillus fumigatus* 6145, *Cunninghamella elegans* var. *elegans* 6992, *Filobasidium* *neoformans* 7902, *Fusarium merismoides* 6153, *Gliocladium deliquescens* 6173, *Glomerella cingulata* 6075, *Hormoconis resinae* 6966, *Kluyveromyces marxianus* 7155, *Mortierella ramanniana* var. *angulispora* 6137, *Monascus rubber* 6122, *Mucor hiemalis* 26779, *Penicillium chrysogenum* 6933, *Saccharomycodes ludwigii* 7126, *Torulaspora delbrueckii* 7116*, Trichoderma koningii* 6042, *Tremella mesenterica* 7131, *Aspergillus niger* KCCM 60332, *Aspergillus oryzae* 60345, *Mucor plumbeus* 60265, and *Rhizopus oryzae* 60556. All the microorganisms were stored with 20% glycerol at −60 °C.

Three types of media were used including potato dextrose medium (24 g/L), malt medium (malt extract 20 g/L, dextrose 20 g/L, peptone 1 g/L), and yeast-malt medium (D-glucose 10 g/L, peptone 5 g/L, malt extract 3 g/L, and yeast extract 3 g/L).

### 3.4. Microbial Screening Procedure

All preliminary screening experiments were performed by a standard two-stage procedure [37,38]. The actively growing microbial cultures were inoculated in 100 mL flasks containing 20 mL of media and incubated for 24 h in a temperature-controlled shaking incubator operating at 200 rpm and 25 °C. Yakuchinone A (**1**) (2 mg) was dissolved in ethanol (100 μL) and added to each Erlenmeyer flask. During the fermentation period, general sampling and TLC monitoring were performed at an interval of 24 h in order to determine the capability of the microorganisms to transform **1**. Culture controls consisted of fermentation cultures in which the microorganisms were grown without the addition of substrates under identical conditions.

### 3.5. Microbial Transformation of Yakuchinone A

Preparative scale fermentation was carried out with the fungus *M*. *hiemalis* in 500 mL flasks with 150 mL of malt medium under the same conditions. The culture flasks each containing 6 mg of **1**, were incubated for 15 h, then extracted with an equal amount of EtOAc three times, and the combined organic layer was concentrated using a rotary evaporator. The EtOAc extracts (210 mg) were subjected to reversed-phase HPLC and eluted with a gradient solvent system of MeOH-H_2_O (55:45→68:32) at a flow rate of 1 mL/min to obtain **3** (23 mg, t_R_ = 12.6 min), and **9** (2.35 mg, t_R_ = 22.0 min), together with fractions A1 (2.8 mg, t_R_ = 11.5 min), A2 (22.43 mg, t_R_ = 17.9 min), and A3 (15.16 mg, t_R_ = 19.4 min). Fraction A1 was further purified using HPLC eluted with a gradient solvent system of MeOH-H_2_O (65:35→75:25) at a flow rate of 2 mL/min to yield **8** (2.02 mg, t_R_ = 14.9 min). Fraction A2 was further separated using a gradient solvent system of MeOH-H_2_O (65:35→75:25) at a flow rate of 2 mL/min to yield **5** (15.9 mg, t_R_ = 17.2 min) and **6** (2.63 mg, t_R_ = 17.9 min). Fraction A3 was further purified using a gradient solvent system of MeOH-H_2_O (55:45→65:35) at a flow rate of 1 mL/min to yield **4** (5.3 mg, t_R_ = 14.0 min) and **7** (7.3 mg, t_R_ = 14.9 min). To obtain compound **2**, the scale-up procedure was further performed and the cultures were harvested within 8 h. The EtOAc extract was subjected to HPLC and eluted with a gradient solvent system of MeOH-H_2_O (65:35→85:15) at a flow rate of 2 mL/min to afford **2** (4.92 mg, t_R_ = 33.3 min).

The metabolite **3** was effectively separated by HPLC using a chiral column eluted with an isocratic solvent system of hexane-EtOH (88:12) at a flow rate of 1 mL/min to obtain **3a** (9.19 mg, t_R_ = 25.0 min), and **3b** (8.77 mg, t_R_ = 27.8 min). Additionally, other metabolites were checked using the same method.

(3*S*)-Oxyphyllacinol (**2**): colorless oil; ${\left[\alpha \right]}_{D}^{20}$ –8.33° [*c* 0.60, MeOH]; UV (*c* 0.1, MeOH) λ_max_ (log ε) 212, 280 nm; IR ν_max_ 2920, 2856, 1602, 1515, 1270 cm^−1^; ^1^H (500 MHz) and ^13^C (125 MHz) NMR data, see Table 1; HRFDMS *m/z* 314.1878 [M]^+^ (calcd for C_20_H_26_O_3_, 314.1877).

(3*S,7R*)-7-Hydroxyoxyphyllacinol (**3a**): colorless oil; ${\left[\alpha \right]}_{D}^{20}$ –7.00° [*c* 0.73, MeOH]; UV (*c* 0.1, MeOH) λ_max_ (log ε) 212, 280 nm; ^1^H (500 MHz) and ^13^C (125 MHz) NMR data, see Table 1; HRFDMS *m/z* 330.1824 [M]^+^ (calcd for C_20_H_26_O_4_, 330.1826).

(3*S,7S*)-7-Hydroxyoxyphyllacinol (**3b**): colorless oil; ${\left[\alpha \right]}_{D}^{20}$ –11.74° [*c* 0.86, MeOH]; UV (*c* 0.1, MeOH) λ_max_ (log ε) 212, 280 nm; ^1^H (500 MHz) and ^13^C (125 MHz) NMR data, see Table 1; HRFDMS *m/z* 330.1823 [M]^+^ (calcd for C_20_H_26_O_4_, 330.1826).

(3*S*)-Oxyphyllacinol-4′-*O*-β-d-glucopyranoside (**4**): yellow oil; ${\left[\alpha \right]}_{D}^{20}$ –29.35° [*c* 0.55, MeOH]; UV (*c* 0.1, MeOH) λ_max_ (log ε) 216, 275 nm; IR ν_max_ 2930, 1585, 1512, 1273 cm^−1^; ^1^H (500 MHz) and ^13^C (125 MHz) NMR data, see Table 1; HRFDMS *m/z* 476.2413 [M]^+^ (calcd for C_26_H_36_O_8_, 476.2405).

(3*S*)-4″-Hydroxyoxyphyllacinol (**5**): colorless oil; ${\left[\alpha \right]}_{D}^{20}$ –12.12° [*c* 0.66, MeOH]; UV (*c* 0.1, MeOH) λ_max_ (log ε) 225, 279 nm; IR ν_max_ 3306, 1637 cm^−1^; ^1^H (500 MHz) and ^13^C (125 MHz) NMR data, see Table 2; HRFDMS *m/z* 330.1810 [M]^+^ (calcd for C_20_H_26_O_4_, 330.1826).

(3*S*)-3″-Hydroxyoxyphyllacinol (**6**): colorless oil; ${\left[\alpha \right]}_{D}^{20}$ –11.16° [*c* 0.36, MeOH]; UV (*c* 0.1, MeOH) λ_max_ (log ε) 205, 278 nm; IR ν_max_ 3307, 1637 cm^−1^; ^1^H (500 MHz) and ^13^C (125 MHz) NMR data, see Table 2; HRFDMS *m/z* 330.1821 [M]^+^ (calcd for C_20_H_26_O_4_, 330.1826).

(3*S*)-2″-Hydroxyoxyphyllacinol (**7**): colorless oil; ${\left[\alpha \right]}_{D}^{20}$ +10.23° [*c* 0.43, MeOH]; UV (*c* 0.1, MeOH) λ_max_ (log ε) 219, 279 nm; IR ν_max_ 3321, 2929, 1643 cm^−1^; ^1^H (500 MHz) and ^13^C (125 MHz) NMR data, see Table 2; HRFDMS *m/z* 330.1832 [M]^+^ (calcd for C_20_H_26_O_4_, 330.1826).

(3*S*)-2″Hydroxyoxyphyllacinol-2″-*O*-β-d-glucopyranoside (**8**): yellow oil; ${\left[\alpha \right]}_{D}^{20}$–15.00° [*c* 0.32, MeOH]; UV (*c* 0.1, MeOH) λ_max_ (log ε) 205, 275 nm; IR ν_max_ 3321, 2946, 2934, 1019 cm^−1^; ^1^H (500 MHz) and ^13^C (125 MHz) NMR data, see Table 2; HRFDMS *m/z* 492.2370 [M]^+^ (calcd for C_26_H_36_O_9_, 492.2354).

(3*S*)-Oxyphyllacinol-3-*O*-β-d-glucopyranoside (**9**): yellow oil; ${\left[\alpha \right]}_{D}^{20}$ –23.57° [*c* 0.42, MeOH]; UV (*c* 0.1, MeOH) λ_max_ (log ε) 206, 281 nm; IR ν_max_ 3321, 2946, 2935 cm^−1^; ^1^H (500 MHz) and ^13^C (125 MHz) NMR data, see Table 2; HRFDMS *m/z* 476.2387 [M]^+^ (calcd for C_26_H_36_O_8_, 476.2405).

### 3.6. Preparation of Mosher’s Esters

(*R*)- and (*S*)-MTPA ester derivatives of compounds (**2**, **3a**, **3b**, **5**, **6**, and **7**) were prepared using the Mosher ester procedure [18]. 1 mg of each compound was completely dried under vacuum, resuspended in 0.5 mL pyridine-*d_5_*, and transferred into a clean NMR tube. 6 μL of (*R*)-(−)-α-methoxy-α-(trifluoromethyl)phenylacetyl chloride (MTPA-chloride) was added into the NMR tube immediately under vacuum and the tube was shaken up carefully. Then the mixture was kept at room temperature for 48 h and afforded the (*S*)-MTPA ester derivatives (**2*_S_***, **3a*_S_***, **3b*_S_***, **5*_S_***, **6*_S_***, and **7*_S_***). The (*R*)-MTPA ester derivatives (**2*_R_***, **3a*_R_***, **3b*_R_***, **5*_R_***, **6*_R_***, and **7*_R_***) were prepared from (*S*)-(+)-MTPA-chloride following the same procedures.

^1^H NMR data (pyridine-*d*_5_, 500 MHz) for **2*_S_***: δ 2.73 (2H, m, H-1), 2.03 (2H, m, H-2), 5.31 (1H, m, H-3), 1.66 (2H, m, H-4), 1.31 (2H, m, H-5), 1.51 (2H, m, H-6), 2.50 (2H, m, H-7). **2*_R_***: δ 2.54 (2H, m, H-1), 1.95 (2H, m, H-2), 5.29 (1H, m, H-3), 1.71 (2H, m, H-4), 1.39 (2H, m, H-5), 1.59 (2H, m, H-6), 2.57 (2H, m, H-7).

^1^H NMR data (pyridine-*d*_5_, 500 MHz) for **3a*_S_***: δ 2.71 (2H, m, H-1), 2.05 (2H, m, H-2), 5.30 (1H, m, H-3), 1.70 (2H, m, H-4), 1.29 (2H, m, H-5), 1.99 (1H, m, H-6a), 1.86 (1H, m, H-6b), 6.07 (1H, t, *J* = 7.0 Hz, H-7), 7.37 (2H, m, H-2″/H-6″). **3a*_R_***: δ 2.51 (2H, m, H-1), 1.86 (2H, m, H-2), 5.23 (1H, m, H-3), 1.60 (2H, m, H-4), 1.34 (2H, m, H-5), 2.01 (1H, m, H-6a), 1.90 (1H, m, H-6b), 6.22 (1H, dd, *J* = 6.1, 6.2 Hz, H-7), 7.54 (2H, m, H-2″/H-6″). **3b*_S_***: δ 2.71 (2H, m, H-1), 2.01 (2H, m, H-2), 5.26 (1H, m, H-3), 1.61 (2H, m, H-4), 1.25 (2H, m, H-5), 2.01 (1H, m, H-6a), 1.74 (1H, m, H-6b), 6.14 (1H, dd, *J* = 6.0, 6.1 Hz, H-7), 7.50 (2H, m, H-2″/H-6″). **3b*_R_***: δ 2.53 (2H, m, H-1), 1.92 (2H, m, H-2), 5.29 (1H, m, H-3), 1.73 (2H, m, H-4), 1.48 (2H, m, H-5), 2.11 (1H, m, H-6a), 1.87 (1H, m, H-6b), 6.15 (1H, t, *J* = 7.0 Hz, H-7), 7.38 (2H, m, H-2″/H-6″).

^1^H NMR data (pyridine-*d*_5_, 500 MHz) for **5*_S_***: δ 2.75 (2H, m, H-1), 2.08 (2H, m, H-2), 5.34 (1H, m, H-3), 1.64 (2H, m, H-4), 1.29 (2H, m, H-5), 1.50 (2H, m, H-6), 2.50 (2H, m, H-7). **5*_R_***: δ 2.58 (2H, m, H-1), 1.95 (2H, m, H-2), 5.33 (1H, m, H-3), 1.71 (2H, m, H-4), 1.39 (2H, m, H-5), 1.59 (2H, m, H-6), 2.55 (2H, m, H-7).

^1^H NMR data (pyridine-*d*_5_, 500 MHz) for **6*_S_***: δ 2.73 (2H, m, H-1), 2.03 (2H, m, H-2), 5.31 (1H, m, H-3), 1.64 (2H, m, H-4), 1.26 (2H, m, H-5), 1.50 (2H, m, H-6), 2.52 (2H, m, H-7). **6*_R_***: δ 2.55 (2H, m, H-1), 1.93 (2H, m, H-2), 5.30 (1H, m, H-3), 1.69 (2H, m, H-4), 1.37 (2H, m, H-5), 1.55 (2H, m, H-6), 2.56 (2H, m, H-7).

^1^H NMR data (pyridine-*d*_5_, 500 MHz) for **7*_S_***: δ 2.53 (2H, m, H-1), 1.92 (2H, m, H-2), 5.31 (1H, m, H-3), 1.63 (2H, m, H-4), 1.28 (2H, m, H-5), 1.43 (2H, m, H-6), 2.47 (2H, m, H-7). **7*_R_***: δ 2.53 (2H, m, H-1), 1.92 (2H, m, H-2), 5.31 (1H, m, H-3), 1.72 (2H, m, H-4), 1.40 (2H, m, H-5), 1.51 (2H, m, H-6), 2.51 (2H, m, H-7).

### 3.7. Hydrolysis of Compounds ***4***, ***8***, and ***9***

Compound **4**, **8**, or **9** (1 mg) dissolved in 1 N HCl was heated for 2 h. After cooling, each reaction mixture was neutralized and extracted with EtOAc [38]. The aqueous layer was concentrated and the sugar unit was detected by means of TLC with authentic D-glucose. The aglycones of **4**, **9**, and **8** in the EtOAc extract were determined as the corresponding metabolites **2** and **7** by HPLC analyses.

### 3.8. Cytotoxicity Assay

Cytotoxic activity against murine melanoma (B16F1 and B16F10), human melanoma (A375P), human lung carcinoma (A549), human breast adenocarcinoma (MCF-7), and human colorectal adenocarcinoma (HT-29) cell lines, which were obtained from the Korean Cell Line Bank (Seoul, Korea), was evaluated using the modified 3-(4,5-dimethylthiazolyl-2)-2,5-diphenyltetrazolium bromide (MTT) assay [38]. Briefly, the cells were cultured in DMEM medium containing penicillin (100 units/mL)-streptomycin (100 µg/mL) and 5% heat-inactivated fetal bovine serum (FBS) in a humidified atmosphere with 5% CO_2_ at 37 °C. Cancer cells were seeded (5 × 10^3^ cells/well) in 96-well plates and incubated for 24 h. Then the old medium was replaced with compound solutions at different concentrations (5, 10, 20, 40, and 80 μM), and further incubated for 24 h (B16, A375, MCF-7, HT-29) or 48 h (A549). After incubation, the medium was aspirated and the MTT solution was added to each well. The MTT solution was discarded after 4 h and DMSO was added to each well to dissolve the formed formazan. The absorbance was measured using a microplate reader at 490 nm. 5-Fluorouracil (5-FU) and demethylzeylasteral (DZ) were used as the positive control, and 0.2% DMSO was used as a negative control. The IC_50_ values were calculated by inhibiting 50% proliferation of the cancer cell population. The IC_50_ values of all tested compounds were calculated and shown in Table 3.

## 4. Conclusions

In summary, microbial transformation of yakuchinone A (**1**) by the fungus *M*. *hiemalis* provided nine new metabolites (**2**, **3a**, **3b**, and **4**–**9**) through reduction, hydroxylation, and glucosylation reactions. Notably, the metabolites were determined to have a 3*S* configuration using the modified Mosher’s method. Subsequently, the absolute configurations at C-7 position for compounds **3a** and **3b** were elucidated as *R* and *S*, respectively. Cytotoxicity evaluation of substrate **1** and its metabolites (**2**, **3a**, **3b**, and **4**–**9**) has been conducted to investigate the structure-activity relationship towards six different cancer cell lines. In contrast with compound **1**, which displayed potent cytotoxic activities against all cancer cell lines, the metabolites **4**–**9** selectively inhibited the proliferation of melanoma cell lines (A375P and B16). In particular, compound **9** with a glucose moiety on the alkyl chain exhibited the most selective cytotoxic activities with the IC_50_ values ranging from 6.09 to 9.74 μM.

## Figures and Tables

**Table 1:** ^1^H and ^13^C NMR data for **2**, **3a**, **3b**, and **4**.

C No.	2		3a		3b		4	
	δ_H_^a^ (*J*/Hz)	δ_C_^a^	δ_H_^b^ (*J*/Hz)	δ_C_^b^	δ_H_^b^ (*J*/Hz)	δ_C_^b^	δ_H_^a^ (*J*/Hz)	δ_C_^a^
1	2.55 m	32.8	2.43 m	31.1	2.43 m	31.1	2.56 m	31.2
	2.67 m		2.56 m		2.56 m		2.70 m	
2	1.66 m	40.7	1.54 m	39.4	1.54 m	39.4	1.69 m	39.0
3	3.51 m	71.8	3.34	69.0	3.34	69.1	3.51 m	70.2
4	1.47 m	38.4	1.32 m	37.1	1.32 m	37.1	1.47 m	36.8
5	1.41 m	26.5	1.33 m	21.7	1.33 m	21.7	1.37 m	25.0
6	1.61 m	32.9	1.57 m	39.7	1.57 m	39.7	1.61 m	31.4
7	2.60 t (7.6)	37.0	4.48 m	72.4	4.48 m	72.3	2.60 m	35.5
1′	-	135.5	-	133.3	-	133.3	-	137.5
2′	6.75 d (1.8)	113.3	6.70 d (1.8)	112.4	6.70 d (1.8)	112.4	6.84 d (1.8)	112.6
3′	-	148.9	-	147.3	-	147.3	-	149.3
4′	-	144.0	-	144.3	-	144.3	-	144.6
5′	6.69 d (8.0)	116.2	6.65 d (8.1)	115.2	6.64 d (8.1)	115.2	7.08 d (8.3)	116.9
6′	6.61 dd	121.9	6.54 dd	120.2	6.54 dd	120.2	6.74 dd	120.5
	(8.0, 1.8)		(8.1, 1.8)		(8.1, 1.8)		(8.3, 1.8)	
1″	-	145.6	-	146.5	-	146.5	-	142.4
2″	7.14	129.5	7.30 d (4.4)	125.8	7.29 d (4.6)	125.8	7.15	128.0
3″	7.23	129.4	7.30 d (4.4)	127.9	7.29 d (4.6)	127.9	7.23	127.8
4″	7.12	126.8	7.20 m	126.5	7.20 m	126.5	7.13	125.3
5″	7.23	129.4	7.30 d (4.4)	127.9	7.29 d (4.6)	127.9	7.23	127.8
6″	7.14	129.5	7.30 d (4.4)	125.8	7.29 d (4.6)	125.8	7.15	128.0
1‴	-	-	-	-	-	-	4.83 d (7.7)	101.7
2‴	-	-	-	-	-	-	3.48 m	73.5
3‴	-	-	-	-	-	-	3.46 m	76.4
4‴	-	-	-	-	-	-	3.39 m	69.9
5‴	-	-	-	-	-	-	3.38 m	76.7
6‴	-	-	-	-	-	-	3.69 m	61.1
							3.87 m	
3-OH	-	-	4.32 d (4.4)	-	4.32 d (4.4)	-	-	-
7-OH	-	-	5.07 d (4.4)	-	5.07 d (4.4)	-	-	-
4′-OH	-	-	8.60 s	-	8.60 s	-	-	-
3′-OCH_3_	3.81 s	56.5	3.73 s	55.5	3.72 s	55.5	3.82 s	55.3

^a^ Data were measured in CD_3_OD. ^b^ Data were measured in DMSO-*d*_6_.

**Table 2:** ^1^H and ^13^C NMR data for **5**–**9**.

C No.	5		6		7		8		9	
	δ_H_ (*J*/Hz)	δ_C_	δ_H_ (*J*/Hz)	δ_C_	δ_H_ (*J*/Hz)	δ_C_	δ_H_ (*J*/Hz)	δ_C_	δ_H_ (*J*/Hz)	δ_C_
1	2.52 m	32.7	2.55 m	32.8	2.53 m	32.8	2.54 m	32.9	2.62 m	31.9
	2.65 m		2.66 m		2.67 m		2.68 m			
2	1.66 m	40.7	1.63 m	40.8	1.64 m	40.8	1.69 m	40.8	1.79 m	37.5
3	3.51 m	71.8	3.52 m	71.8	3.58, m	71.9	3.51 m	71.9	3.69 m	80.3
4	1.46 m	38.4	1.48 m	38.4	1.46 m	38.6	1.47 m	38.4	1.59 m	37.0
5	1.44 m	26.4	1.35m	26.5	1.37 m	26.8	1.37 m	26.7	1.42 m	26.0
6	1.56 m	33.2	1.59 m	32.8	1.58 m	31.3	1.61 m	31.5	1.63 m	32.9
7	2.50 t	36.1	2.53 t	37.0	2.58 t	31.3	2.64 m	31.2	2.62 m	36.0
	(7.4)		(7.3)		(7.7)		2.74 m			
1′	-	135.5	-	135.5	-	135.5	-	135.5	-	135.6
2′	6.75 d	113.3	6.75 d	113.3	6.75 d	113.3	6.76 d	113.3	6.79 d	113.4
	(1.4)		(1.6)		(1.7)		(1.8)		(1.8)	
3′	-	148.9	-	148.9	-	148.9	-	148.9	-	148.9
4′	-	145.5	-	145.6	-	145.5	-	145.5	-	145.5
5′	6.68 d	116.2	6.69 d	116.2	6.68 d	116.2	6.69 d	116.2	6.69 d	116.2
	(7.8)		(8.0)		(8.0)		(8.1)		(7.9)	
6′	6.62 dd	121.9	6.60	121.9	6.62 dd	121.9	6.62 dd	121.9	6.62 dd	121.9
	(7.8, 1.4)				(8.0, 1.7)		(8.1, 1.8)		(7.9, 1.8)	
1″	-	134.9	-	145.6	-	130.3	-	133.6	-	144.2
2″	6.97 d (8.1)	130.4	6.60	116.4	-	156.4	-	157.1	7.15	129.4
3″	6.67 d (8.1)	116.2	-	158.4	6.71	116.0	7.11	116.4	7.23	129.6
4″	-	156.4	6.57 dd	113.7	6.96 td	120.6	6.91 m	123.3	7.11	126.7
			(7.9, 2.1)		(7.7, 1.7)					
5″	6.67 d (8.1)	116.2	7.05 t (7.7)	130.3	6.71	127.8	7.11	128.1	7.23	129.6
6″	6.97 d	130.4	6.63	121.9	7.02 dd	131.2	7.10	131.1	7.15	129.4
	(8.1)				(7.7, 1.7)					
1‴	-	-	-	-	-	-	4.89 d	102.7	4.30 d	104.0
							(7.5)		(7.8)	
2‴	-	-	-	-	-	-	3.48 m	75.2	3.19 m	75.5
3‴	-	-	-	-	-	-	3.40 m	78.2	3.18 m	77.8
4‴	-	-	-	-	-	-	3.40 m	71.6	3.32 m	71.9
5‴	-	-	-	-	-	-	3.46 m	78.5	3.34 m	78.4
6‴	-	-	-	-	-	-	3.69 m	62.7	3.67 m	63.0
							3.89 m		3.89 m	
3′-OCH_3_	3.82 s	56.5	3.82 s	56.5	3.82 s	56.5	3.82 s	56.5	3.82	56.6

All the data were measured in CD_3_OD.

**Table 3:** IC_50_ of all compounds against six cancer cell lines ^a^.

Compound	IC_50_ ± SD (μM)					
	A375P	B16F1	B16F10	A549	MCF-7	HT-29
**1**	14.75 ± 1.00	31.73 ± 4.46	21.71 ± 3.65	26.07 ± 2.08	11.50 ± 0.71	11.96 ± 0.74
**2**	45.52 ± 1.09	56.03 ± 0.74	59.31 ± 0.42	62.92 ± 3.07	42.95 ± 0.80	54.88 ± 4.16
**3**	>80	>80	>80	>80	>80	>80
**3a**	>80	>80	>80	>80	>80	>80
**3b**	>80	>80	>80	>80	>80	>80
**4**	45.62 ± 0.41	22.97 ± 1.94	46.13 ± 3.79	>80	60.04 ± 4.97	>80
**5**	29.58 ± 0.45	17.84 ± 0.76	33.38 ± 2.76	>80	53.66 ± 2.53	72.75 ± 2.50
**6**	47.91 ± 0.54	33.73 ± 2.71	46.55 ± 2.15	>80	51.46 ± 1.66	>80
**7**	38.79 ± 1.47	29.28 ± 0.76	33.58 ± 2.20	>80	34.28 ± 1.76	>80
**8**	75.23 ± 2.89	38.37 ± 0.30	64.58 ± 3.52	>80	76.34 ± 1.35	>80
**9**	8.36 ± 0.05	6.09 ± 0.26	9.74 ± 0.20	>80	37.83 ± 1.50	35.36 ± 1.17
**5-FU ^b^**	4.92 ± 0.28	5.18 ± 0.78	11.22 ± 0.85	-	-	-
**DZ ^b^**	-	-	-	19.98 ± 0.30	8.28 ± 0.13	14.98 ± 0.51

^a^ Results are expressed as the mean values of three experiments ± SD; ^b^ 5-FU: 5-Fluorouracil; DZ: Demethylzeylasteral.

## Data Availability

Not applicable.

## Supplementary Materials

The following are available online at https://www.mdpi.com/article/10.3390/ijms23073992/s1.
