# Supplementary material for: Microbial Transformation of Yakuchinone A and Cytotoxicity Evaluation of Its Metabolites
Source: Int J Mol Sci. 2022 Apr 3;23(7):3992. doi: 10.3390/ijms23073992 (PMC9000044; doi:10.3390/ijms23073992)
Supplement: Supplementary file 1 [file ijms-23-03992-s001.zip › ijms-1664827-SI.pdf]

Supplementary Material

Microbial Transformation of Yakuchinone A and Cytotoxicity Evaluation of Its Metabolites

Chen Huo [1,†], Fubo Han [1,†], Yina Xiao [1], Hyun Jung Kim [2,*] and Ik-Soo Lee [1,*]

[1] College of Pharmacy, Chonnam National University, Gwangju 61186, Korea; huochen_0213@163.com (C.H.); hanfubo0306@gmail.com (F.H.); yogurtxiao@163.com (Y.X.)
[2] College of Pharmacy and Natural Medicine Research Institute, Mokpo National University, Muan, Jeonnam 58554, Korea
* Correspondence: hyunkim@mokpo.ac.kr (H.J.K.); islee@chonnam.ac.kr (I.-S.L.); Tel.: +82-62-530-2932
† These authors contributed equally to this work.

Table S1. Twenty-two microorganisms were incubated for screening of yakuchinone A. 5
Figure S1. $\Delta\delta_H$ ($=\delta_S - \delta_R$) values for the Mosher ester derivatives of **2**, **5**, **6**, and **7**................ 5
Figure S2. ^{1}H-NMR (CD_3OD, 400 MHz) spectrum of yakuchinone A (**1**)........................... 6
Figure S3. ^{13}C-NMR (CD_3OD, 100 MHz) spectrum of yakuchinone A (**1**)........................... 6
Figure S4. COSY spectrum of yakuchinone A (**1**). 7
Figure S5. HSQC spectrum of yakuchinone A (**1**). 7
Figure S6. HMBC spectrum of yakuchinone A (**1**). 8
Figure S7. ^{1}H-NMR (CD_3OD, 500 MHz) spectrum of **2**... 8
Figure S8. ^{13}C-NMR (CD_3OD, 125 MHz) spectrum of **2**... 9
Figure S9. COSY spectrum of **2**. 9
Figure S10. HSQC spectrum of **2**. 10
Figure S11. HMBC spectrum of **2**. 10
Figure S12. HRFDMS spectrum of **2**... 11
Figure S13. ^{1}H-NMR (DMSO-d_6, 400 MHz) spectrum of **3**. 12
Figure S14. ^{13}C-NMR (DMSO-d_6, 100 MHz) spectrum of **3**. 12
Figure S15. COSY spectrum of **3**. 13
Figure S16. HSQC spectrum of **3**. 13
Figure S17. HMBC spectrum of **3**. 14
Figure S18. HRFDMS spectrum of **3**... 15
Figure S19. ^{1}H-NMR (DMSO-d_6, 500 MHz) spectrum of **3a**. 16
Figure S20. ^{13}C-NMR (DMSO-d_6, 125 MHz) spectrum of **3a**. 16
Figure S21. HRFDMS spectrum of **3a**... 17
Figure S22. ^{1}H-NMR (DMSO-d_6, 500 MHz) spectrum of **3b**. 18
Figure S23. ^{13}C-NMR (DMSO-d_6, 125 MHz) spectrum of **3b**. 18
Figure S24. COSY spectrum of **3b**. 19
Figure S25. HSQC spectrum of **3b**. 19
Figure S26. HMBC spectrum of **3b**. 20
Figure S27. HRFDMS spectrum of **3b**. 21
Figure S28. ^{1}H-NMR (CD_3OD, 500 MHz) spectrum of **4**.. 22
Figure S29. ^{13}C-NMR (CD_3OD, 125 MHz) spectrum of **4**.. 22
Figure S30. COSY spectrum of **4**. 23
Figure S31. HSQC spectrum of **4**. 23
Figure S32. HMBC spectrum of **4**. 24

Figure S33. HRFDMS spectrum of **4**. 25
Figure S34. ^{1}H-NMR (CD_3OD, 500 MHz) spectrum of **5**. 26
Figure S35. ^{13}C-NMR (CD_3OD, 125 MHz) spectrum of **5**. 26
Figure S36. COSY spectrum of **5**. 27
Figure S37. HSQC spectrum of **5**. 27
Figure S38. HMBC spectrum of **5**. 28
Figure S39. HRFDMS spectrum of **5**. 29
Figure S40. ^{1}H-NMR (CD_3OD, 500 MHz) spectrum of **6**. 30
Figure S41. ^{13}C-NMR (CD_3OD, 125 MHz) spectrum of **6**. 30
Figure S42. COSY spectrum of **6**. 31
Figure S43. HSQC spectrum of **6**. 31
Figure S44. HMBC spectrum of **6**. 32
Figure S45. HRFDMS spectrum of **6**. 33
Figure S46. ^{1}H-NMR (CD_3OD, 500 MHz) spectrum of **7**. 34
Figure S47. ^{13}C-NMR (CD_3OD, 125 MHz) spectrum of **7**. 34
Figure S48. COSY spectrum of **7**. 35
Figure S49. HSQC spectrum of **7**. 35
Figure S50. HMBC spectrum of **7**. 36
Figure S51. HRFDMS spectrum of **7**. 37
Figure S52. ^{1}H-NMR (CD_3OD, 500 MHz) spectrum of **8**. 38
Figure S53. ^{13}C-NMR (CD_3OD, 125 MHz) spectrum of **8**. 38
Figure S54. COSY spectrum of **8**. 39
Figure S55. HSQC spectrum of **8**. 39
Figure S56. HMBC spectrum of **8**. 40
Figure S57. HRFDMS spectrum of **8**. 41
Figure S58. ^{1}H-NMR (CD_3OD, 500 MHz) spectrum of **9**. 42
Figure S59. ^{13}C-NMR (CD_3OD, 125 MHz) spectrum of **9**. 42
Figure S60. COSY spectrum of **9**. 43
Figure S61. HSQC spectrum of **9**. 43
Figure S62. HMBC spectrum of **9**. 44
Figure S63. HRFDMS spectrum of **9**. 45
Figure S64. ^{1}H-NMR (pyridine-d_5, 500 MHz) spectrum of (*S*)-MTPA ester of **2**. 46
Figure S65. COSY spectrum of (*S*)-MTPA ester of **2**. 46

Figure S66. ^{1}H-NMR (pyridine-d_5, 500 MHz) spectrum of (*R*)-MTPA ester of **2**. 47
Figure S67. COSY spectrum of (*R*)-MTPA ester of **2**. 47
Figure S68. ^{1}H-NMR (pyridine-d_5, 500 MHz) spectrum of (*S*)-MTPA ester of **3a**. 48
Figure S69. NOE spectrum of (*S*)-MTPA ester of **3a**. 48
Figure S70. COSY spectrum of (*S*)-MTPA ester of **3a**. 49
Figure S71. ^{1}H-NMR (pyridine-d_5, 500 MHz) spectrum of (*R*)-MTPA ester of **3a**. 49
Figure S72. NOE spectrum of (*R*)-MTPA ester of **3a**. 50
Figure S73. COSY spectrum of (*R*)-MTPA ester of **3a**. 50
Figure S74. ^{1}H-NMR (pyridine-d_5, 500 MHz) spectrum of (*S*)-MTPA ester of **3b**. 51
Figure S75. NOE spectrum of (*S*)-MTPA ester of **3b**. 51
Figure S76. COSY spectrum of (*S*)-MTPA ester of **3b**. 52
Figure S77. ^{1}H-NMR (pyridine-d_5, 500 MHz) spectrum of (*R*)-MTPA ester of **3b**. 52
Figure S78. NOE spectrum of (*R*)-MTPA ester of **3b**. 53
Figure S79. COSY spectrum of (*R*)-MTPA ester of **3b**. 53
Figure S80. ^{1}H-NMR (pyridine-d_5, 500 MHz) spectrum of (*S*)-MTPA ester of **5**. 54
Figure S81. COSY spectrum of (*S*)-MTPA ester of **5**. 54
Figure S82. ^{1}H-NMR (pyridine-d_5, 500 MHz) spectrum of (*R*)-MTPA ester of **5**. 55
Figure S83. COSY spectrum of (*R*)-MTPA ester of **5**. 55
Figure S84. ^{1}H-NMR (pyridine-d_5, 500 MHz) spectrum of (*S*)-MTPA ester of **6**. 56
Figure S85. COSY spectrum of (*S*)-MTPA ester of **6**. 56
Figure S86. ^{1}H-NMR (pyridine-d_5, 500 MHz) spectrum of (*R*)-MTPA ester of **6**. 57
Figure S87. COSY spectrum of (*R*)-MTPA ester of **6**. 57
Figure S88. ^{1}H-NMR (pyridine-d_5, 500 MHz) spectrum of (*S*)-MTPA ester of **7**. 58
Figure S89. COSY spectrum of (*S*)-MTPA ester of **7**. 58
Figure S90. ^{1}H-NMR (pyridine-d_5, 500 MHz) spectrum of (*R*)-MTPA ester of **7**. 59
Figure S91. COSY spectrum of (*S*)-MTPA ester of **7**. 59

Table S1. Twenty-two microorganisms were incubated for screening of yakuchinone A.

Microorganisms	Metabolite production [a]
Absidia coerulea KCTC 6936	(+)
Alternaria alternata KCTC 6005	(-)
Aspergillus fumigatus KCTC 6145	(-)
Aspergillus niger KCCM 60332	(-)
Aspergillus oryzae KCCM 60345	(-)
Cunninghamella elegans var. *elegans* KCTC 6992	(+)
Filobasidium neoformans KCTC 7902	(-)
Fusarium merismoides KCTC 6153	(-)
Gliocladium deliquescens KCTC 6173	(-)
Glomerella cingulata KCTC 6075	(-)
Hormoconis resinae KCTC 6966	(+)
Kluyveromyces marxianus KCTC 7155	(-)
Mortierella ramanniana var. *angulispora* KCTC 6137	(-)
Monascus rubber KCTC 6122	(-)
Mucor hiemalis KCTC 26779	(+++)
Mucor plumbeus KCCM 60265	(++)
Penicillium chrysogenum KCTC 6933	(-)
Rhizopus oryzae KCCM 60556	(+)
Saccharomycodes ludwigii KCTC 7126	(-)
Torulaspora delbrueckii KCTC 7116	(-)
Trichoderma koningii KCTC 6042	(-)
Tremella mesenterica KCTC 7131	(-)

[a] Metabolite production denoted by (+) indicted that metabolites were produced as shown by TLC analysis.

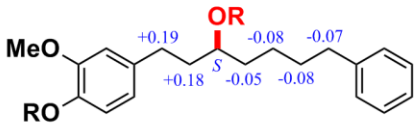

$\mathbf{2}_S$: R = (*S*)-MTPA
$\mathbf{2}_R$: R = (*R*)-MTPA

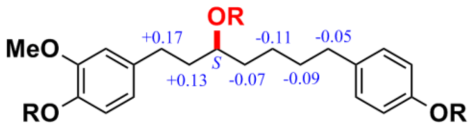

$\mathbf{5}_S$: R = (*S*)-MTPA
$\mathbf{5}_R$: R = (*R*)-MTPA

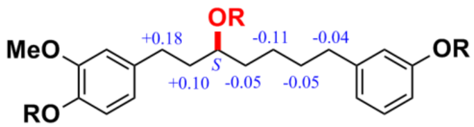

$\mathbf{6}_S$: R = (*S*)-MTPA
$\mathbf{6}_R$: R = (*R*)-MTPA

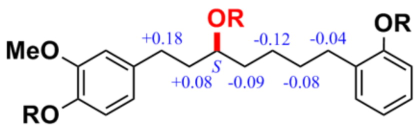

$\mathbf{7}_S$: R = (*S*)-MTPA
$\mathbf{7}_R$: R = (*R*)-MTPA

Figure S1. $\Delta\delta_H$ ($=\delta_S-\delta_R$) values for the Mosher ester derivatives of **2**, **5**, **6**, and **7**.

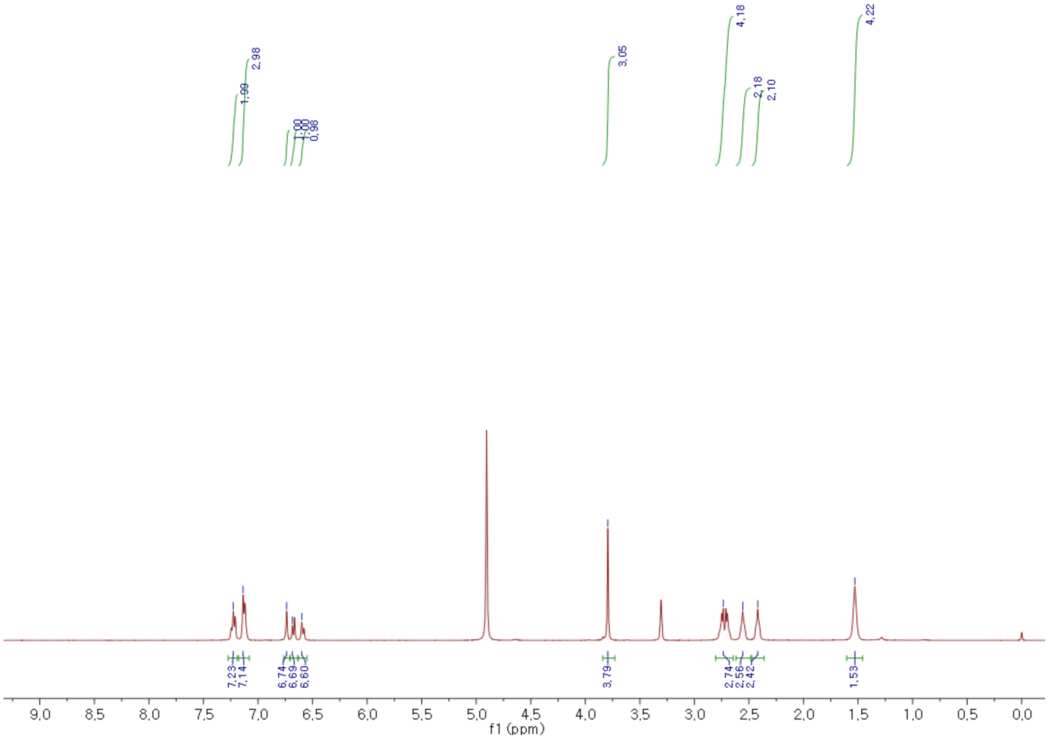

Figure S2. ^{1}H-NMR (CD_3OD, 400 MHz) spectrum of yakuchinone A (**1**).

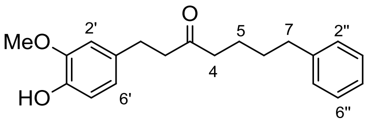

Yakuchinone A (**1**)

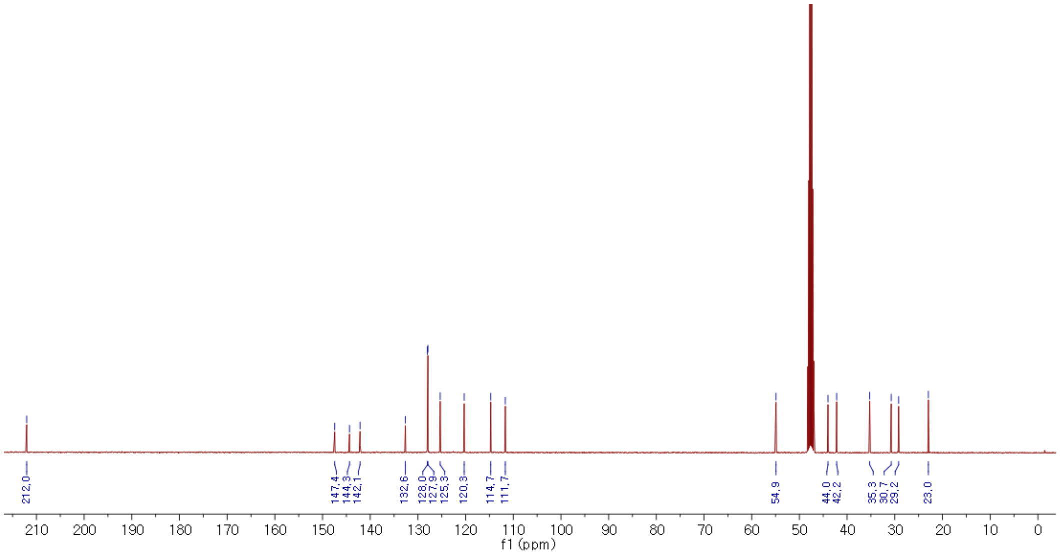

Figure S3. ^{13}C-NMR (CD_3OD, 100 MHz) spectrum of yakuchinone A (**1**).

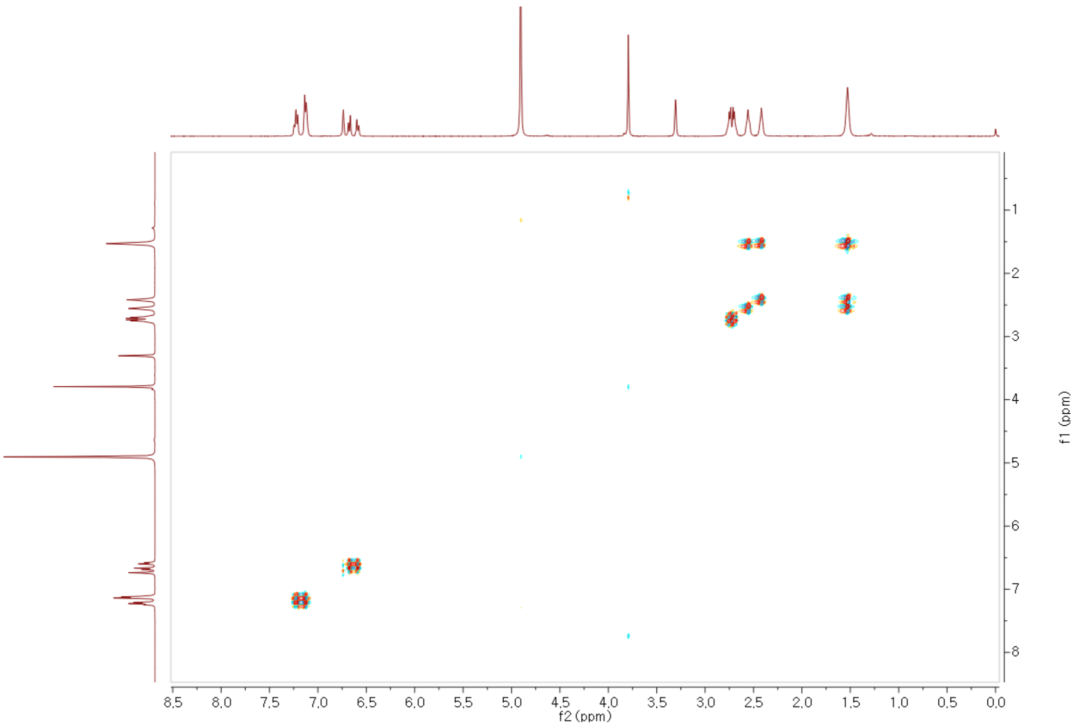

Figure S4. COSY spectrum of yakuchinone A (**1**).

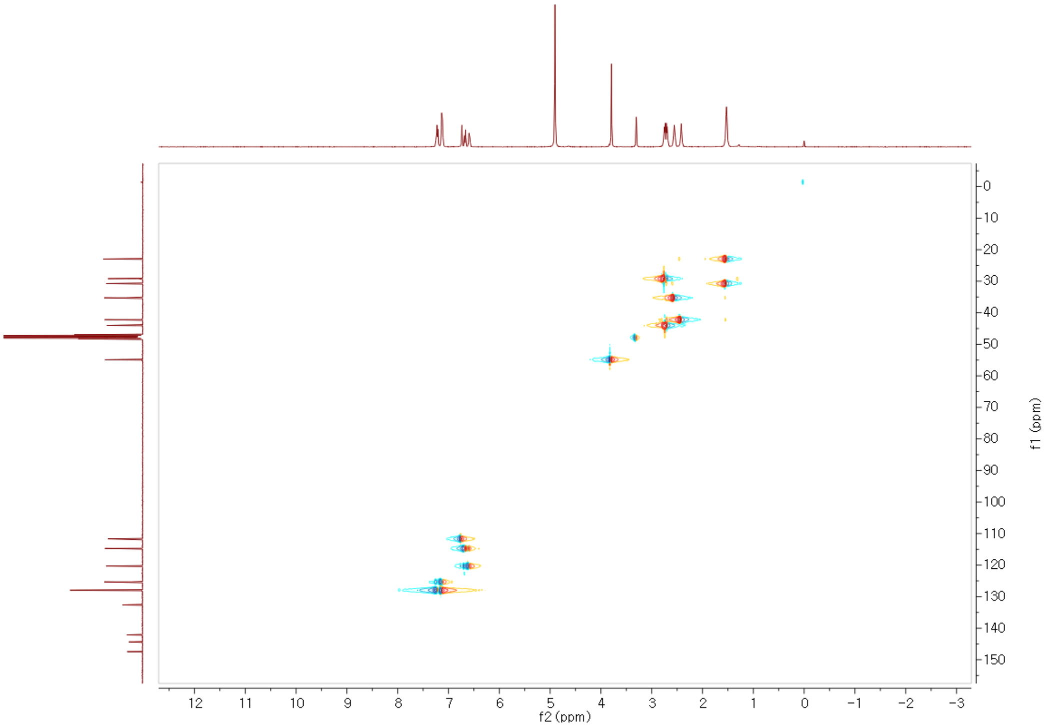

Figure S5. HSQC spectrum of yakuchinone A (**1**).

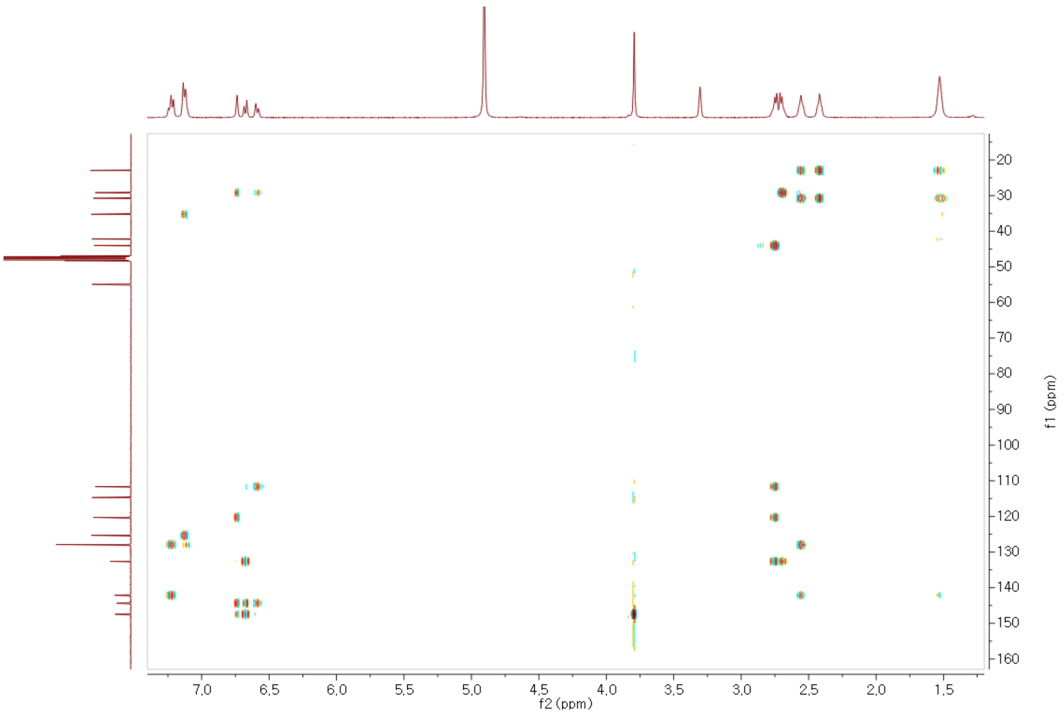

Figure S6. HMBC spectrum of yakuchinone A (**1**).

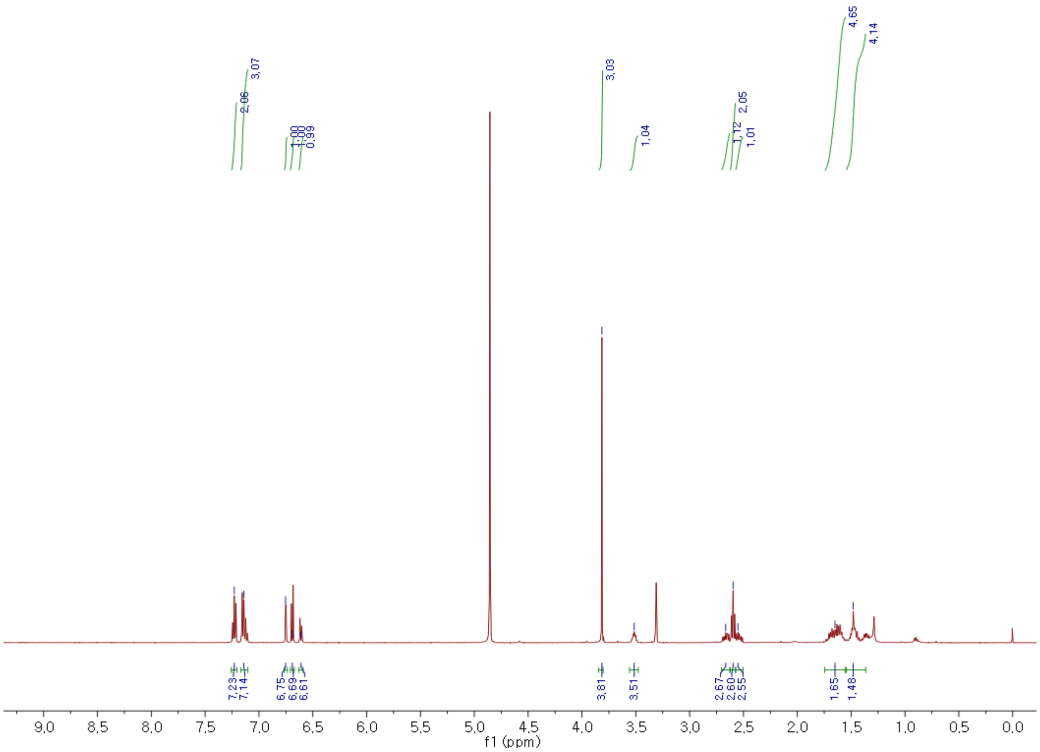

Figure S7. ^{1}H-NMR (CD_3OD, 500 MHz) spectrum of **2**.

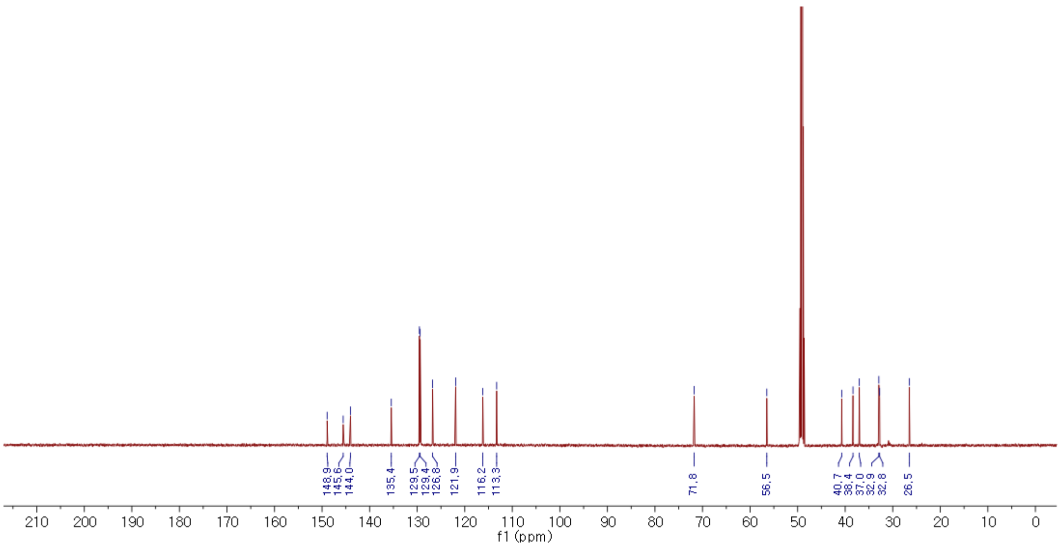

Figure S8. ^{13}C-NMR (CD_3OD, 125 MHz) spectrum of **2**.

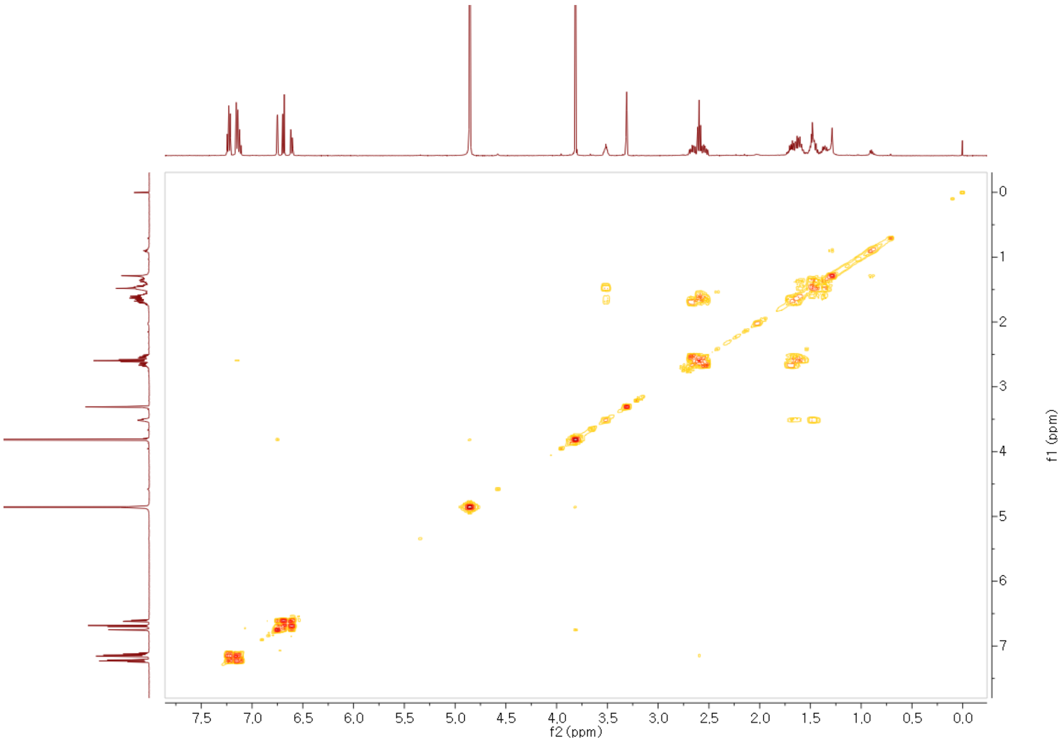

Figure S9. COSY spectrum of **2**.

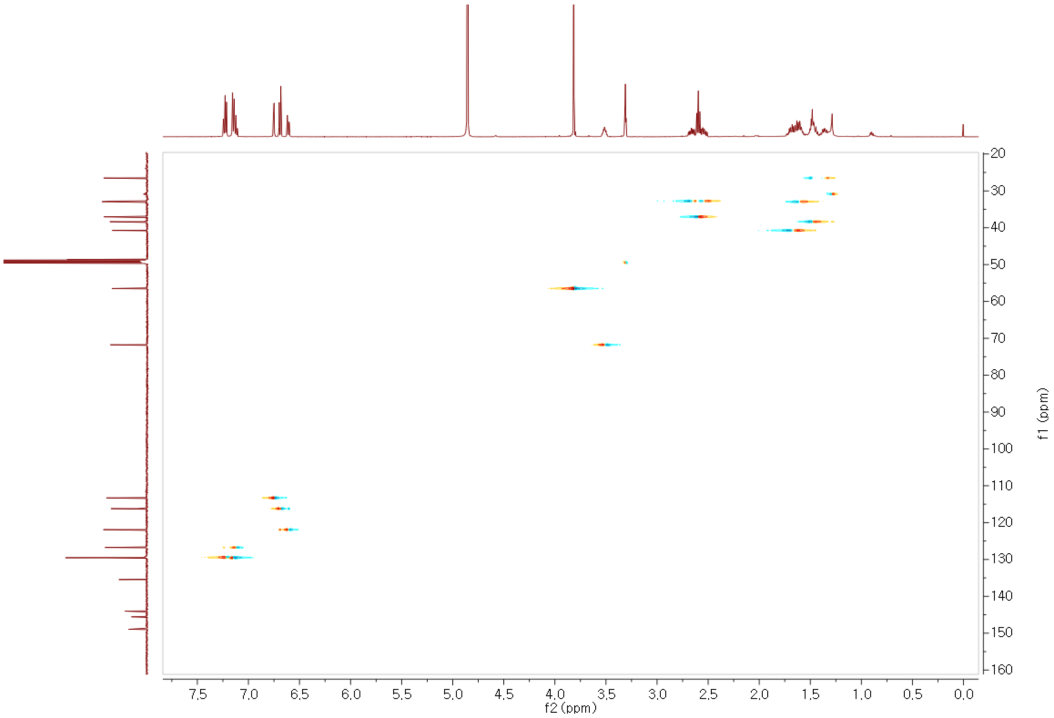

Figure S10. HSQC spectrum of **2**.

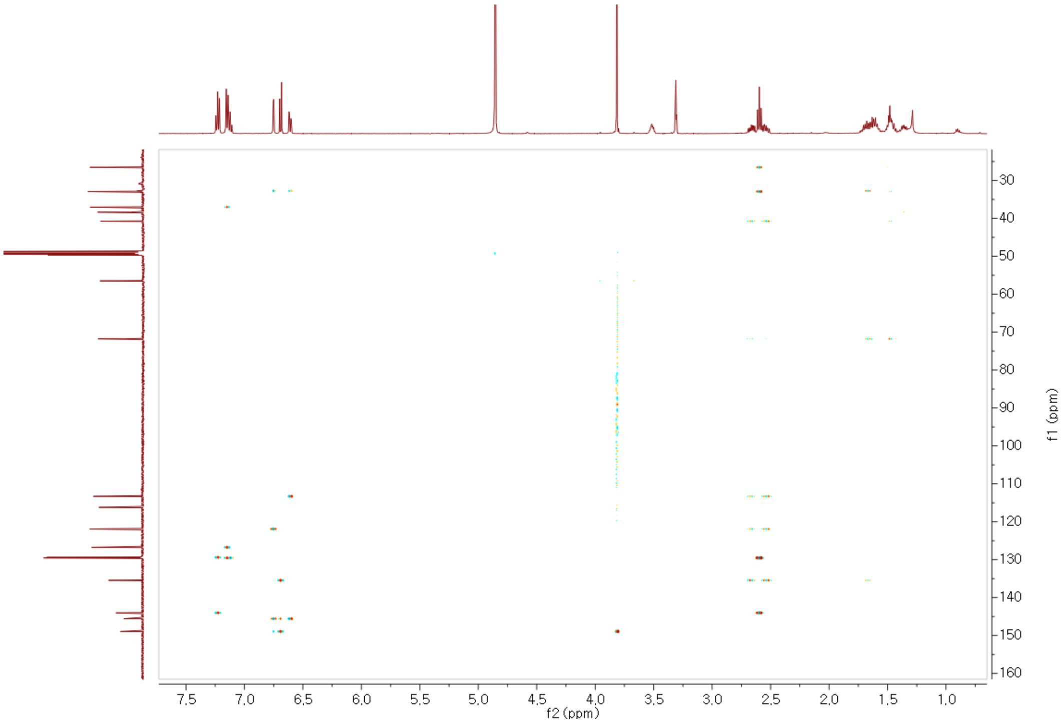

Figure S11. HMBC spectrum of **2**.

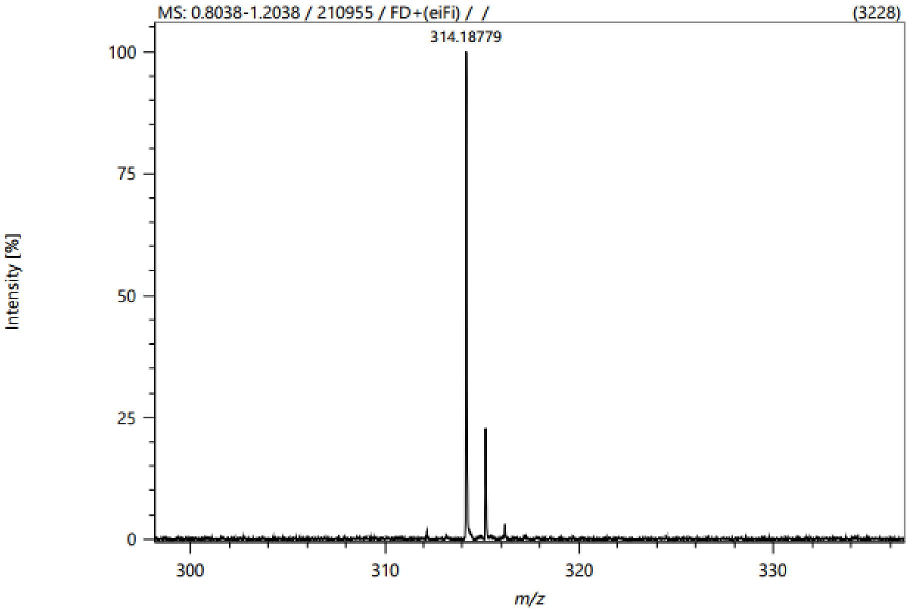

Elemental Composition

Parameters

Tolerance:	30.00 mDa
Electron:	Odd/Even
Charge:	+1
DBE:	-90.0 - 90.0

Elements Set 1:

Symbol	C	H	O
Min	1	5	1
Max	20	26	3

Results

Mass	Intensity	Formula	Calculated Mass	Mass Difference [mDa]	Mass Difference [ppm]	DBE
314.18779	3228.10	C20 H26 O3	314.18765	0.15	0.46	8.0

Figure S12. HRFDMS spectrum of **2**.

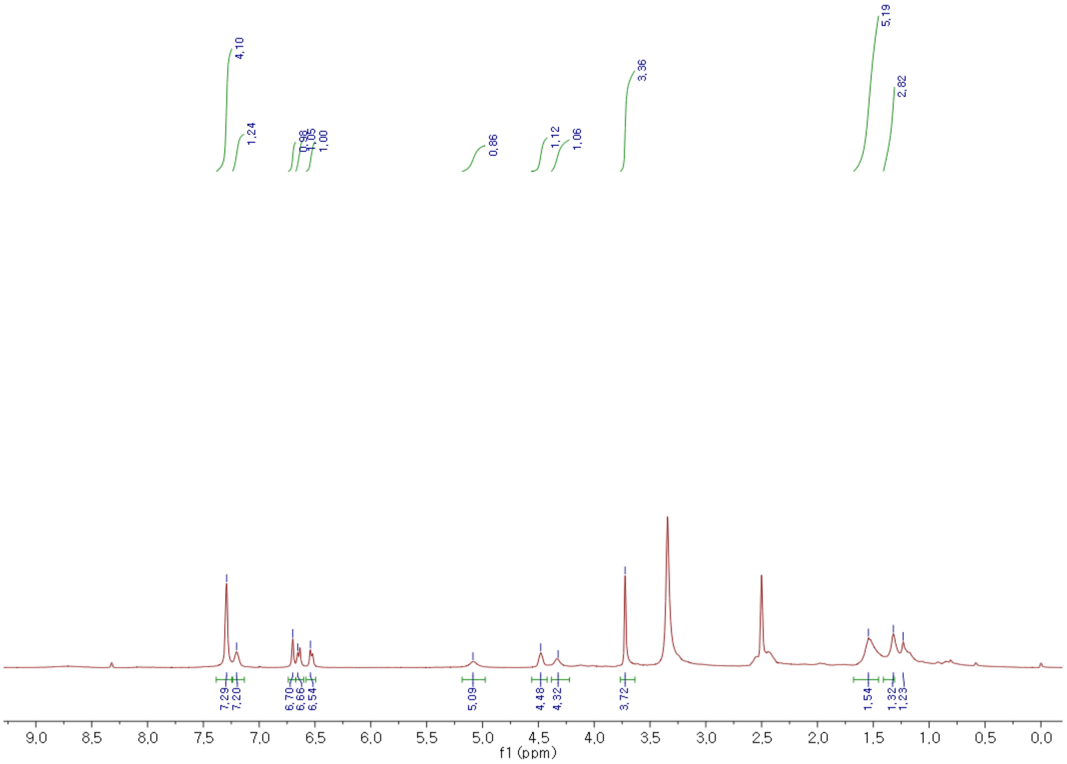

Figure S13. ^{1}H-NMR (DMSO-d_6, 400 MHz) spectrum of **3**.

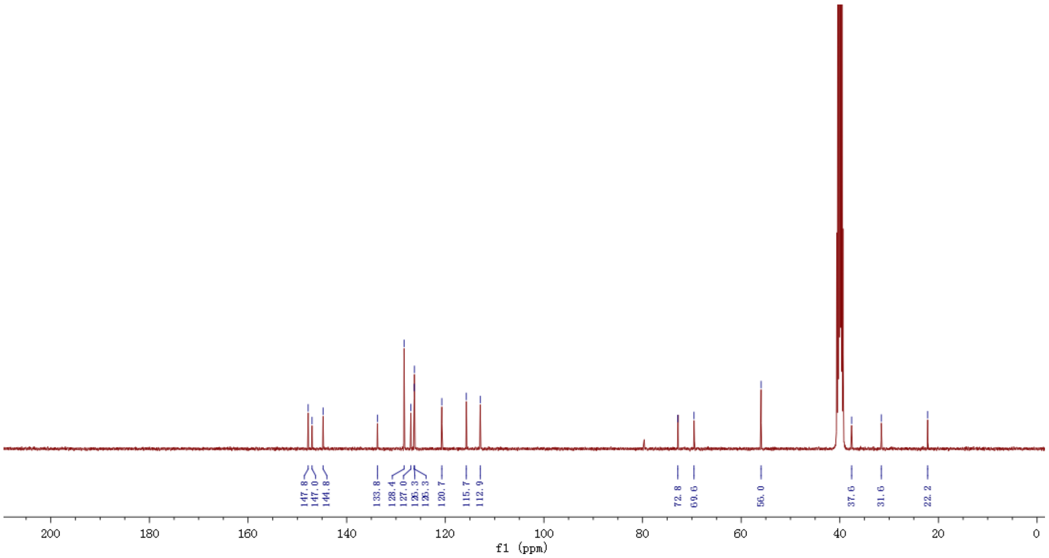

Figure S14. ^{13}C-NMR (DMSO-d_6, 100 MHz) spectrum of **3**.

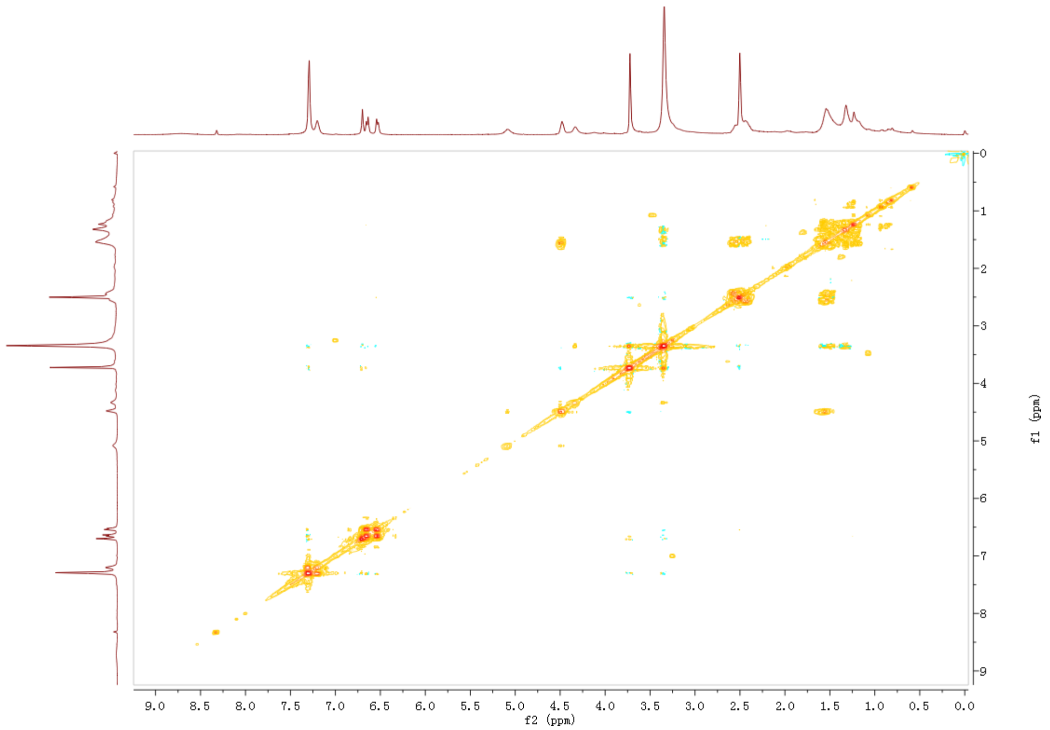

Figure S15. COSY spectrum of **3**.

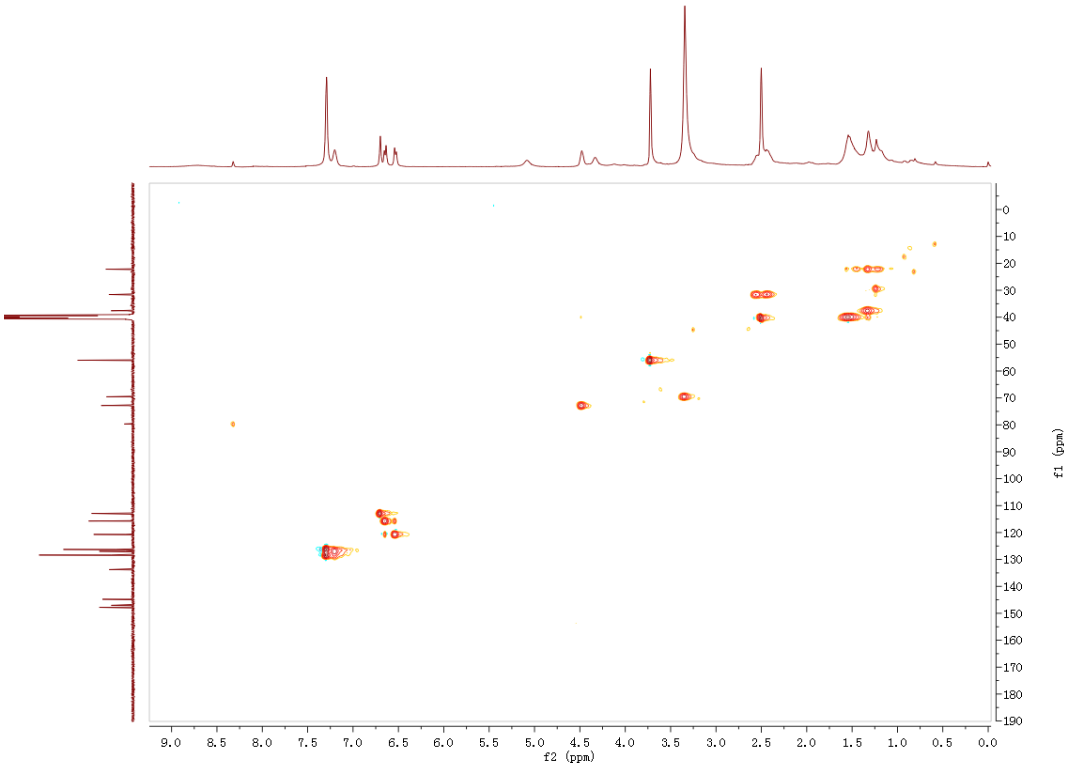

Figure S16. HSQC spectrum of **3**.

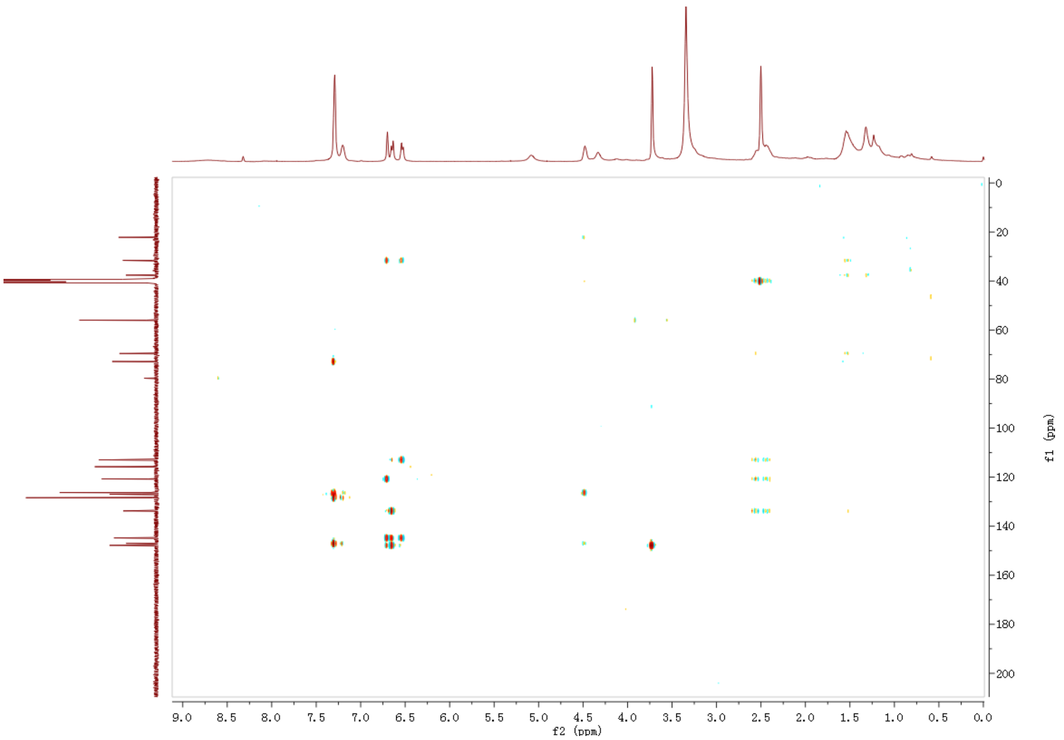

Figure S17. HMBC spectrum of **3**.

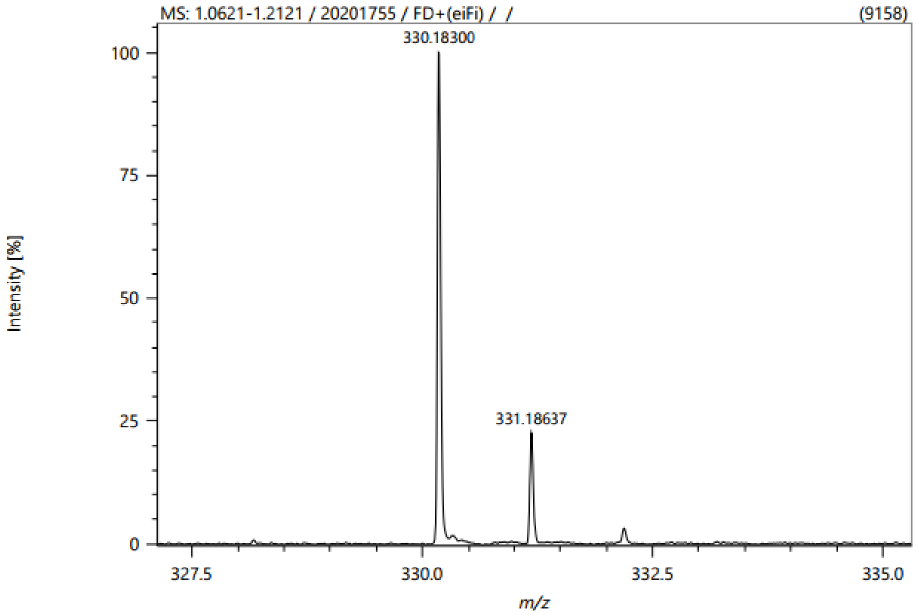

Elemental Composition

Parameters		Elements Set 1:			
Tolerance:	30.00 mDa	Symbol	C	H	O
Electron:	Odd/Even	Min	5	5	1
Charge:	+1	Max	20	26	4
DBE:	-90.0 - 90.0				

Results

Mass	Intensity	Formula	Calculated Mass	Mass Difference [mDa]	Mass Difference [ppm]	DBE
330.18300	9158.13	C20 H26 O4	330.18256	0.44	1.35	8.0

Figure S18. HRFDMS spectrum of **3**.

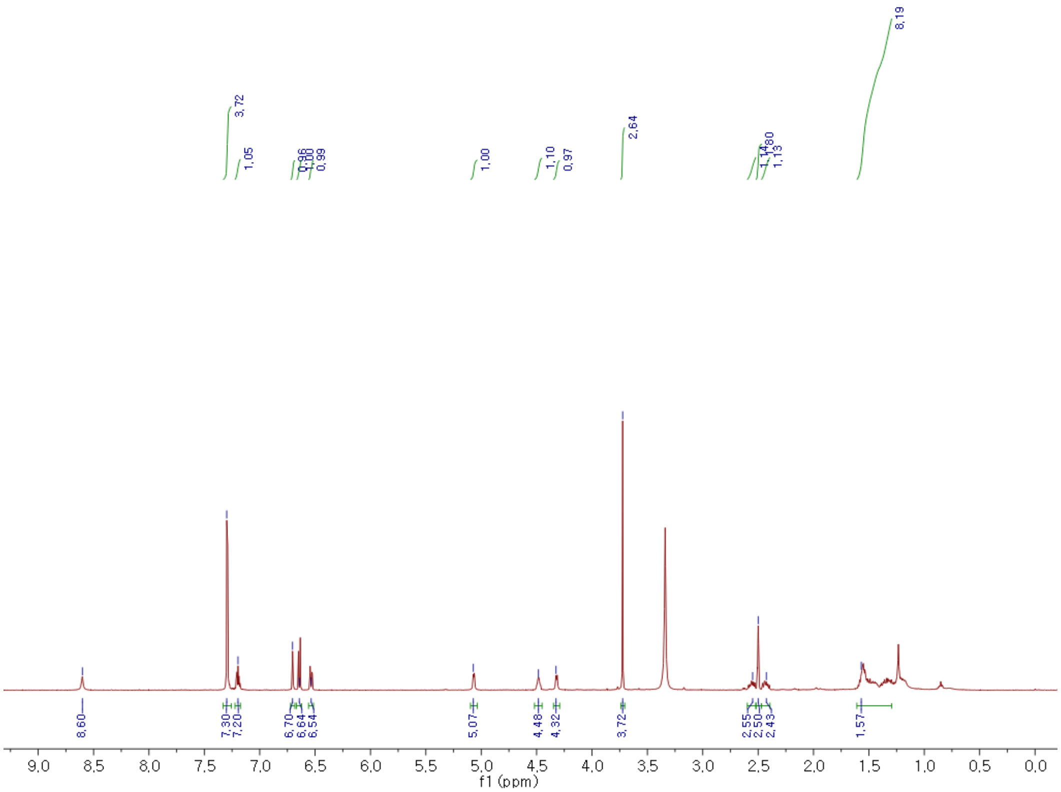

Figure S19. ^{1}H-NMR (DMSO-d_6, 500 MHz) spectrum of **3a**.

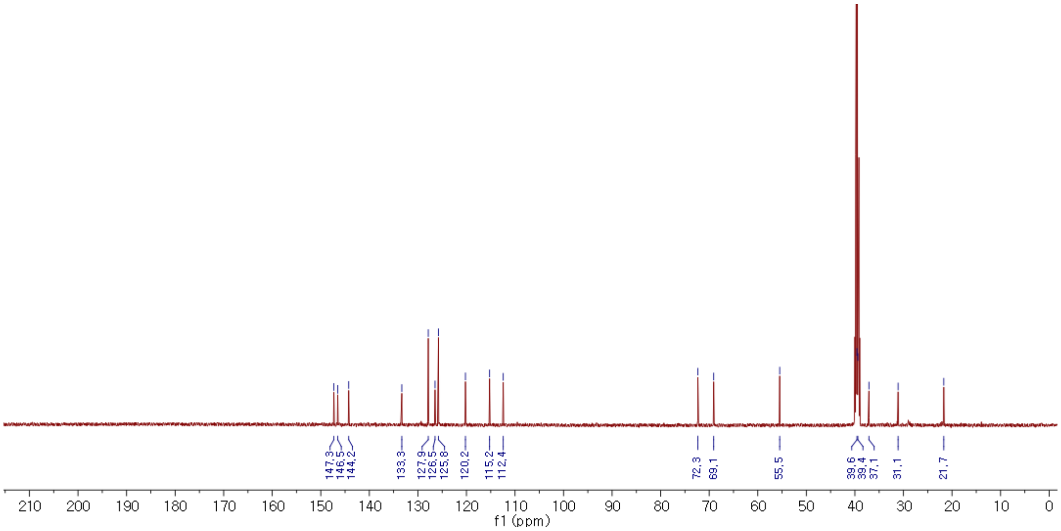

Figure S20. ^{13}C-NMR (DMSO-d_6, 125 MHz) spectrum of **3a**.

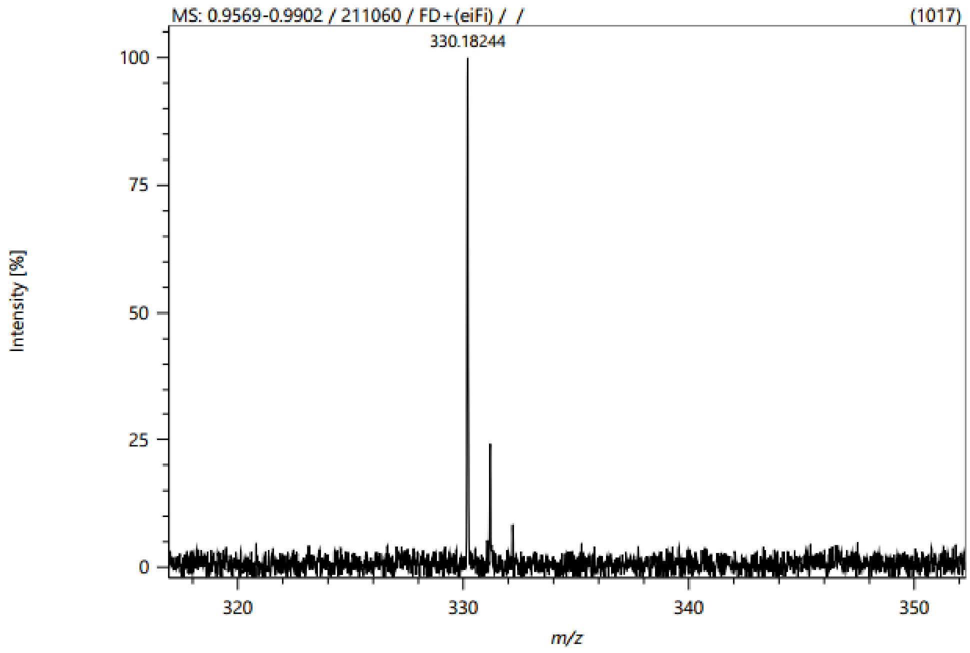

Elemental Composition

Parameters	
Tolerance:	30.00 mDa
Electron:	Odd/Even
Charge:	+1
DBE:	-90.0 - 90.0

Elements Set 1:

Symbol	C	H	O
Min	1	5	1
Max	20	26	4

Results

Mass	Intensity	Formula	Calculated Mass	Mass Difference [mDa]	Mass Difference [ppm]	DBE
330.18244	1016.67	C20 H26 O4	330.18256	-0.13	-0.38	8.0

Figure S21. HRFDMS spectrum of **3a**.

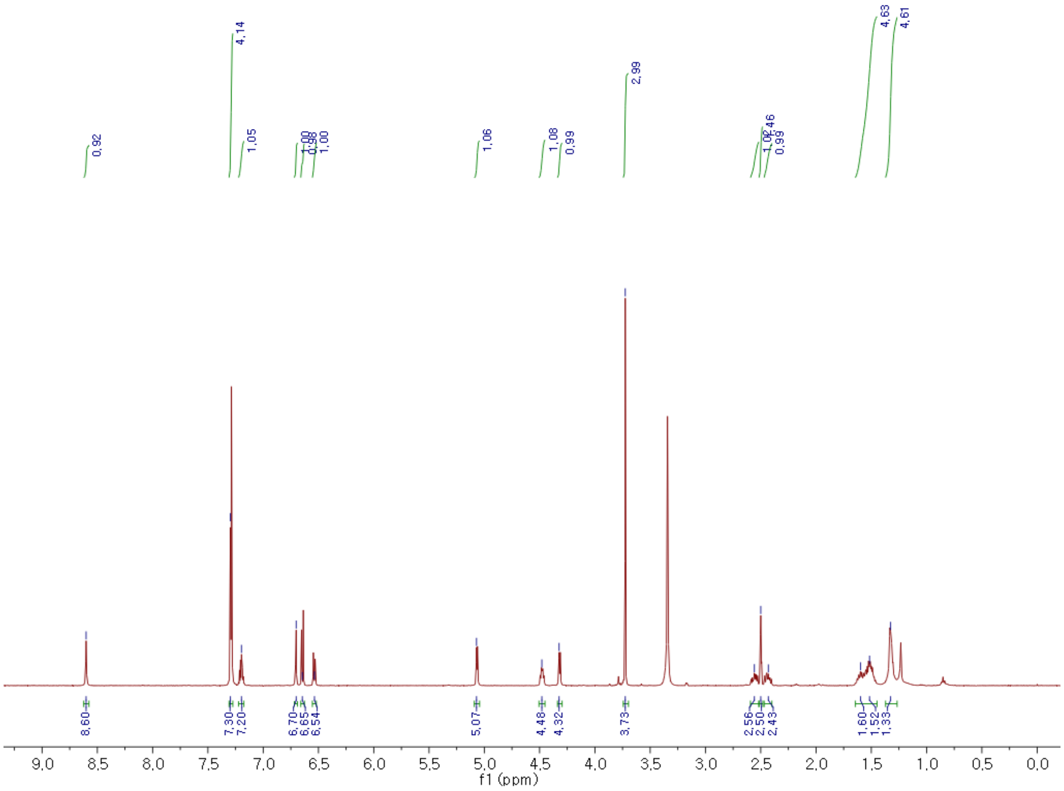

Figure S22. ^{1}H-NMR (DMSO-d_6, 500 MHz) spectrum of **3b**.

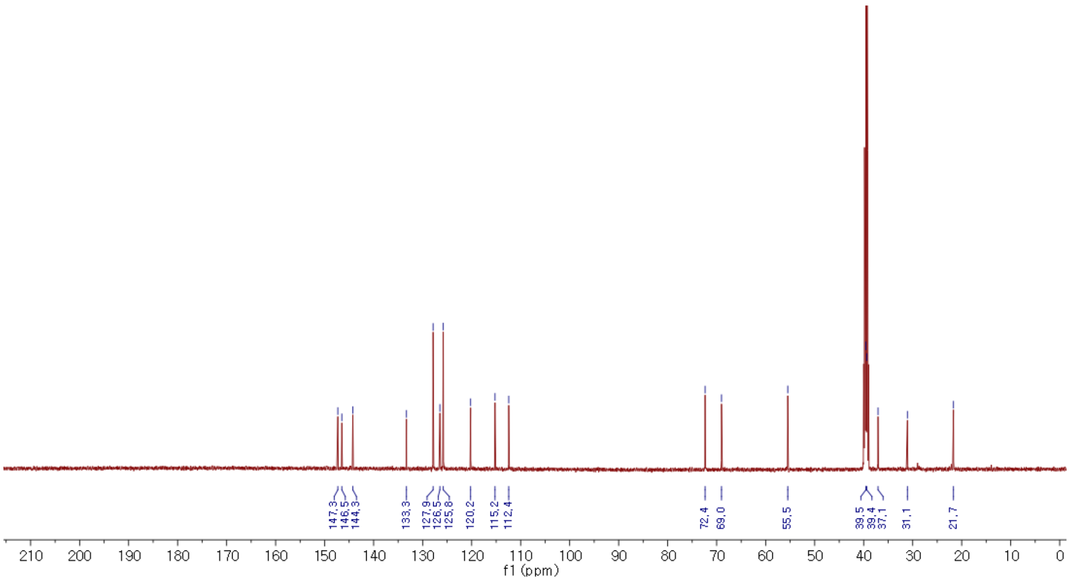

Figure S23. ^{13}C-NMR (DMSO-d_6, 125 MHz) spectrum of **3b**.

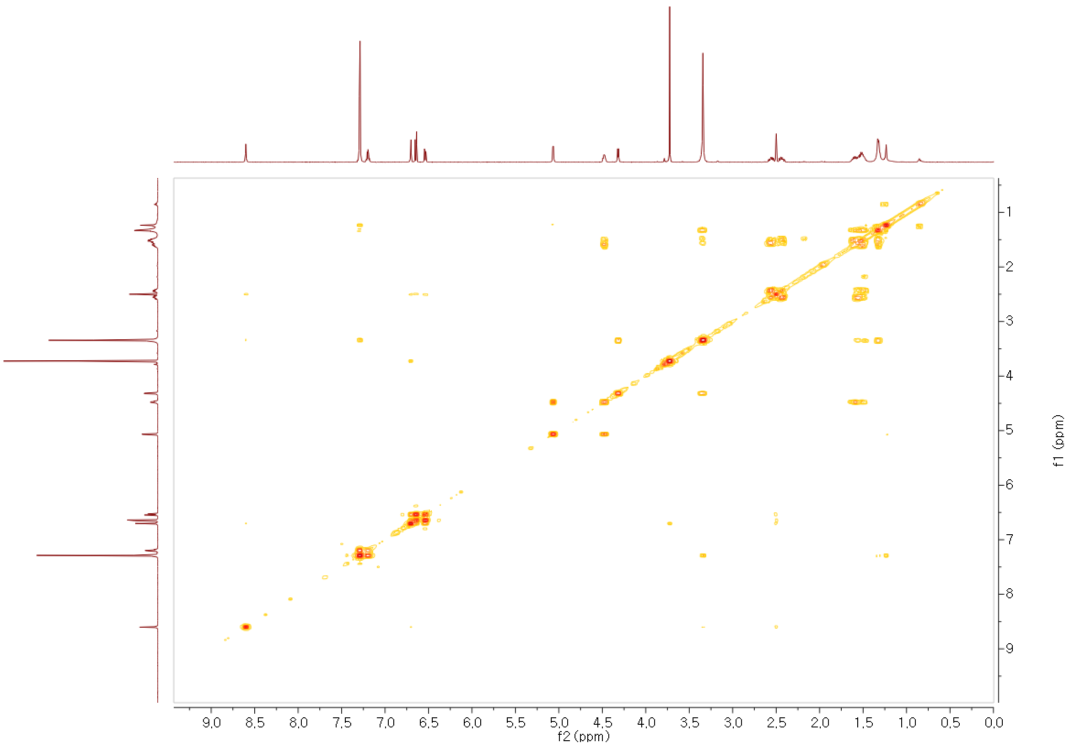

Figure S24. COSY spectrum of **3b**.

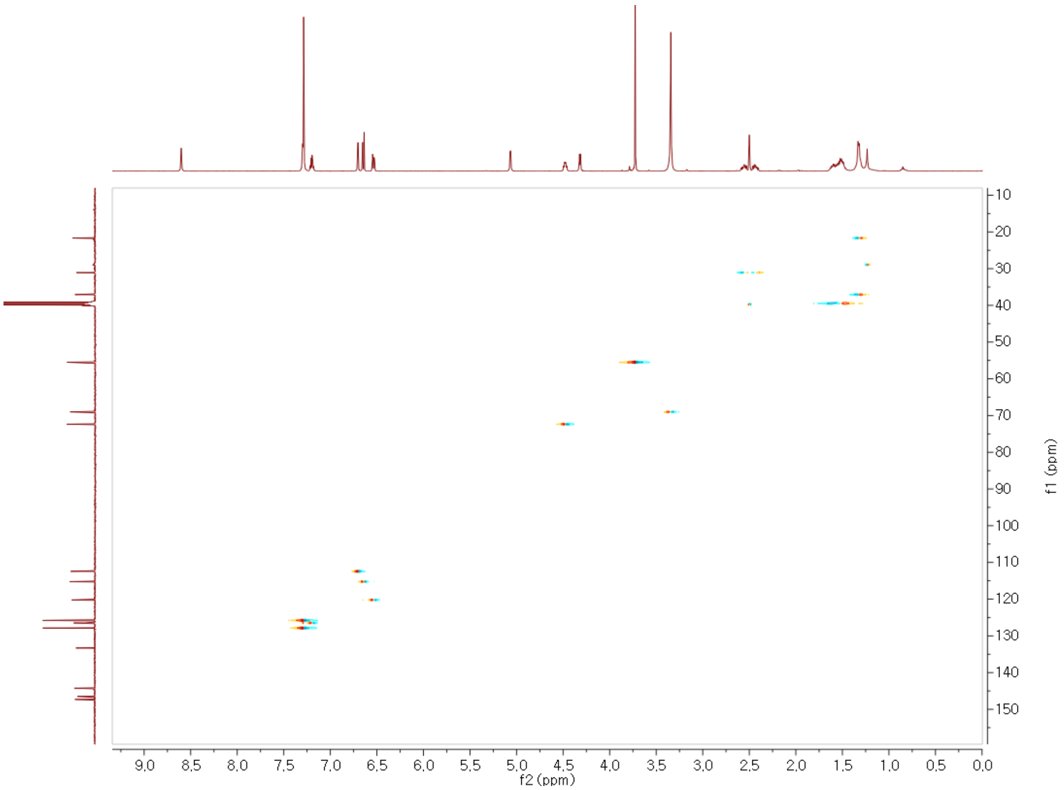

Figure S25. HSQC spectrum of **3b**.

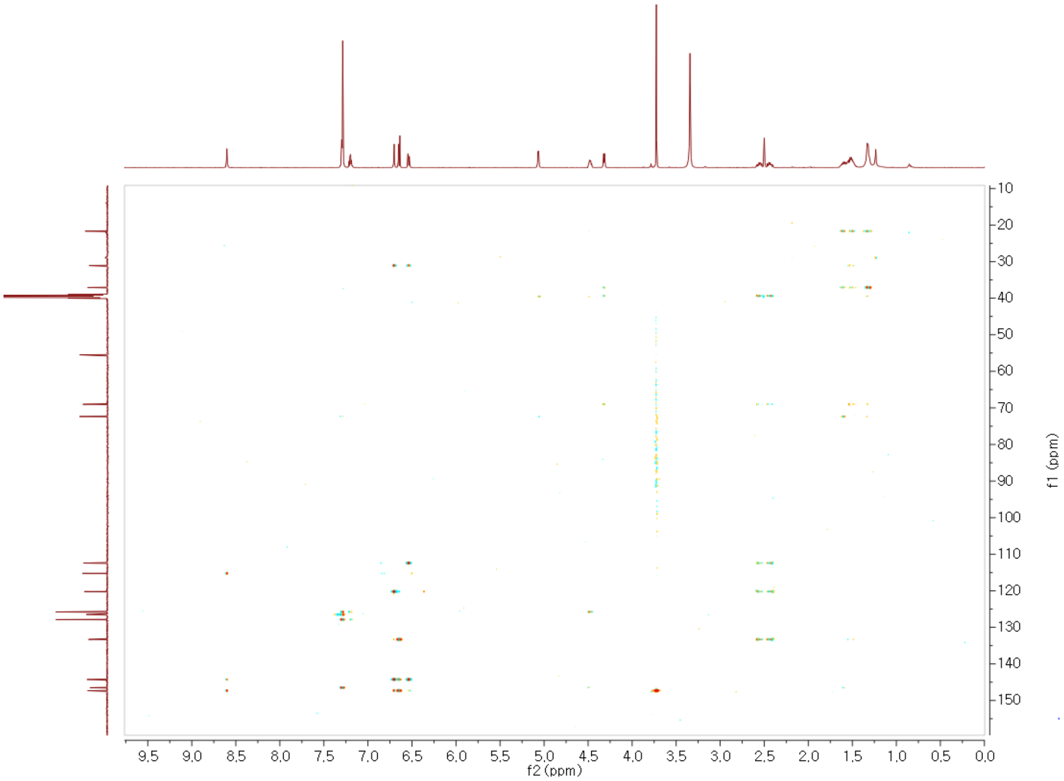

Figure S26. HMBC spectrum of **3b**.

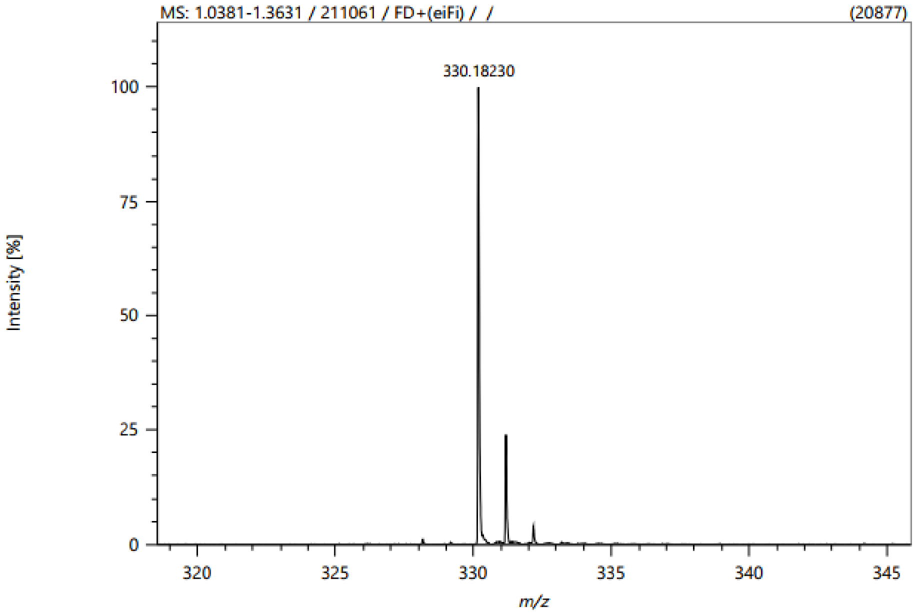

Elemental Composition

Parameters

Tolerance:	30.00 mDa
Electron:	Odd/Even
Charge:	+1
DBE:	-90.0 - 90.0

Elements Set 1:

Symbol	C	H	O
Min	1	5	1
Max	20	26	4

Results

Mass	Intensity	Formula	Calculated Mass	Mass Difference [mDa]	Mass Difference [ppm]	DBE
330.18230	20876.99	C20 H26 O4	330.18256	-0.26	-0.80	8.0

Figure S27. HRFDMS spectrum of **3b**.

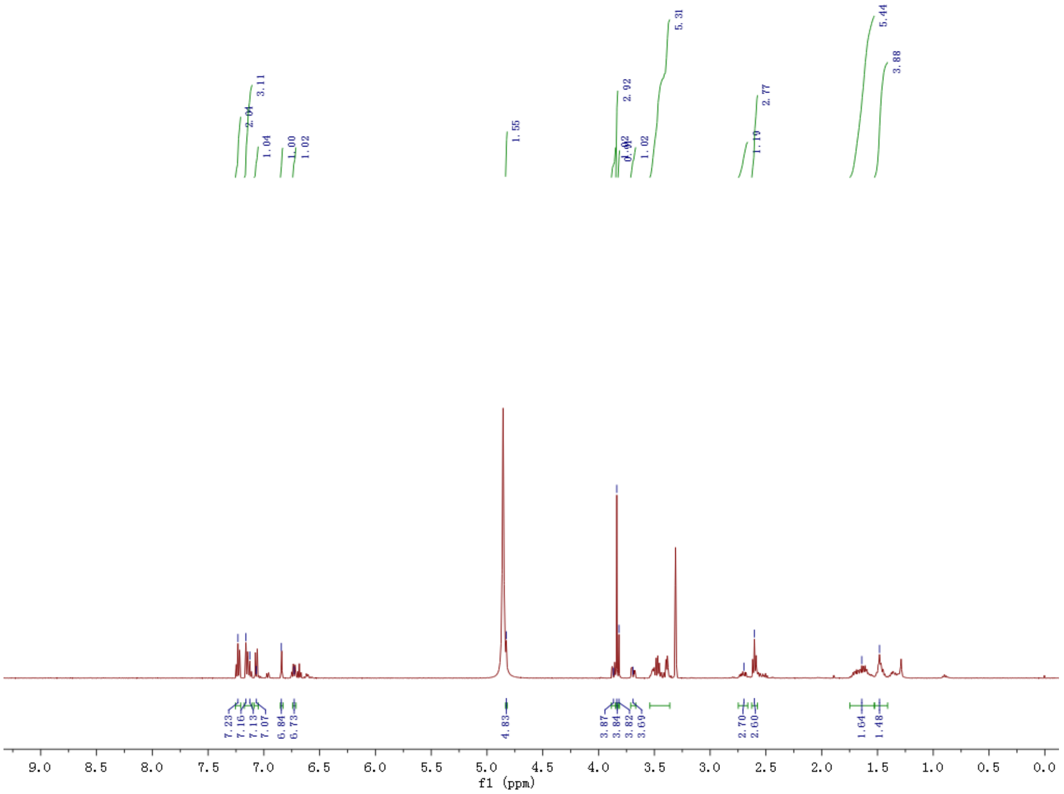

Figure S28. ^{1}H-NMR (CD_3OD, 500 MHz) spectrum of **4**.

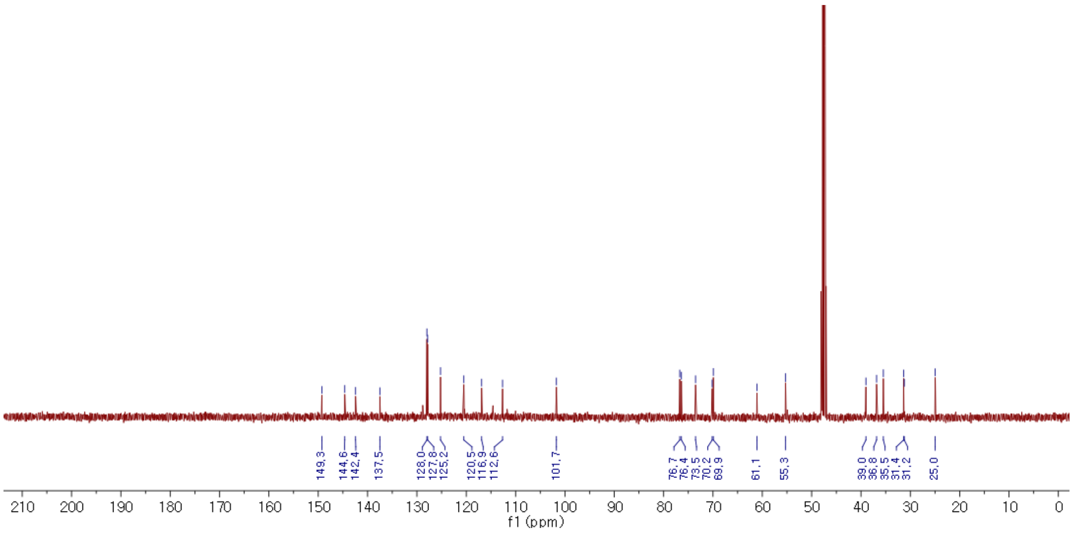

Figure S29. ^{13}C-NMR (CD_3OD, 125 MHz) spectrum of **4**.

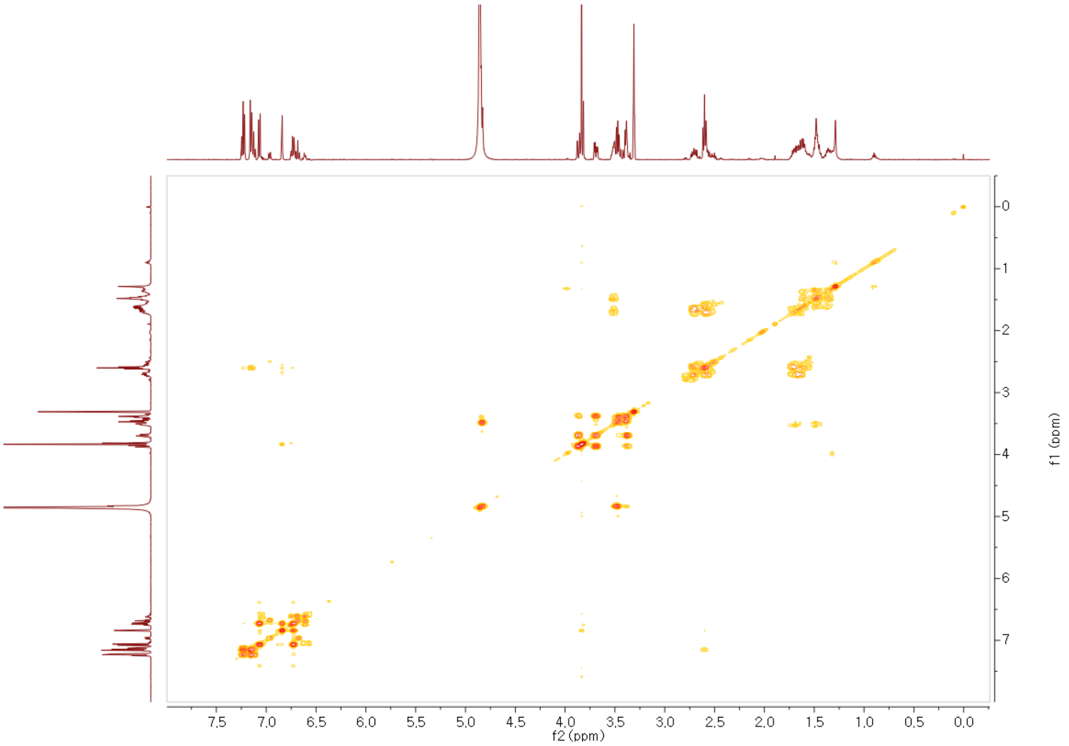

Figure S30. COSY spectrum of **4**.

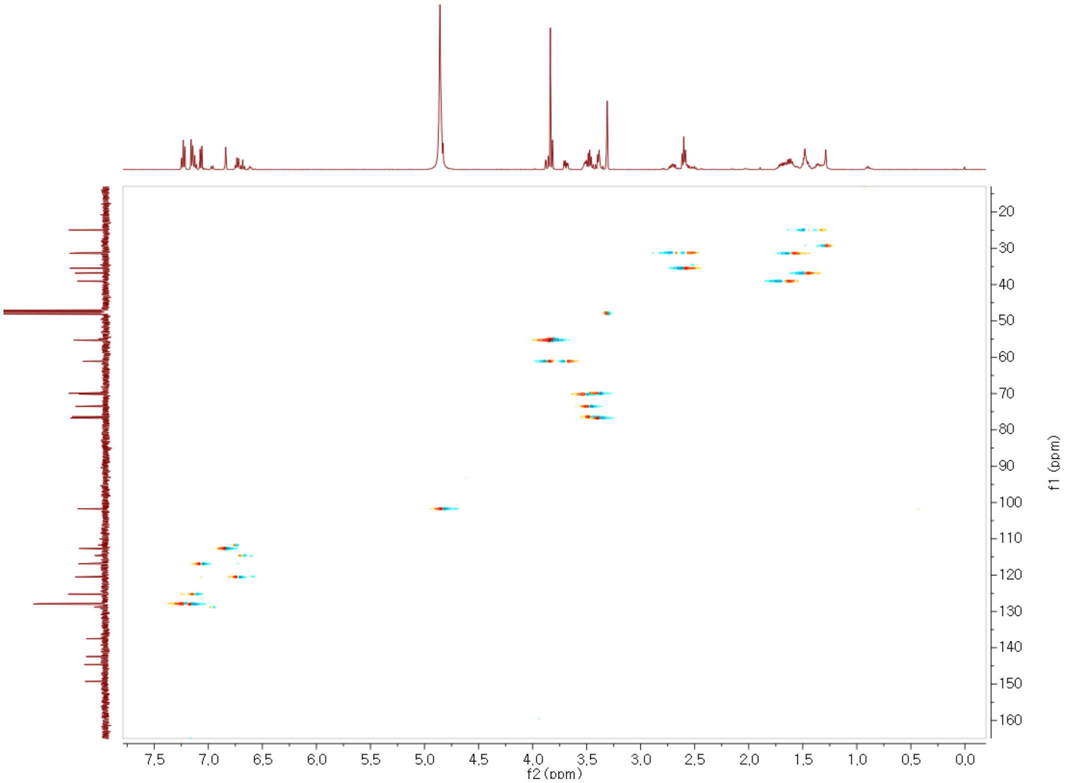

Figure S31. HSQC spectrum of **4**.

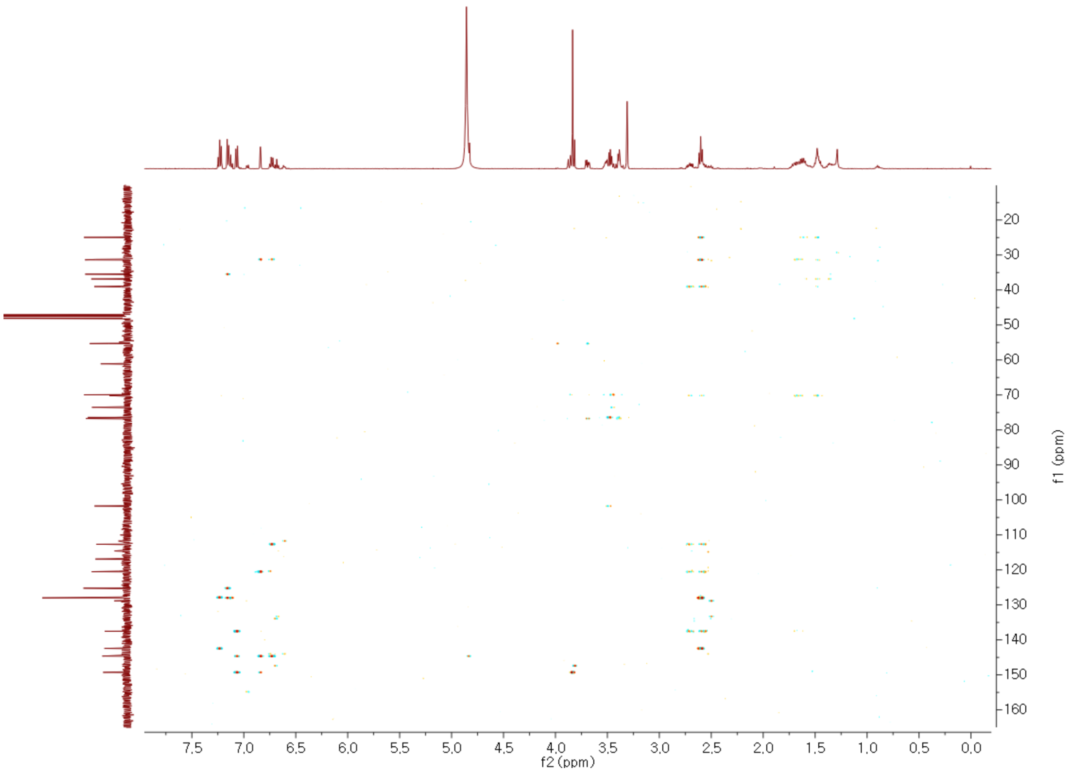

Figure S32. HMBC spectrum of **4**.

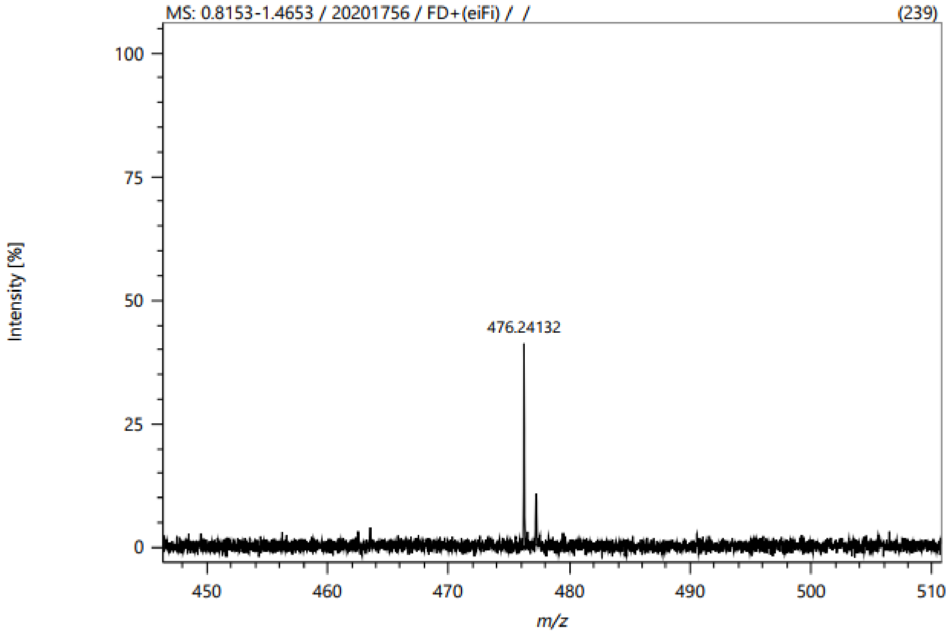

Elemental Composition

Parameters		Elements Set 1:			
		Symbol	C	H	O
Tolerance:	30.00 mDa	Min	5	5	1
Electron:	Odd/Even	Max	26	36	8
Charge:	+1				
DBE:	-90.0 - 90.0				

Results

Mass	Intensity	Formula	Calculated Mass	Mass Difference [mDa]	Mass Difference [ppm]	DBE
476.24132	238.71	C26 H36 O8	476.24047	0.85	1.78	9.0

Figure S33. HRFDMS spectrum of **4**.

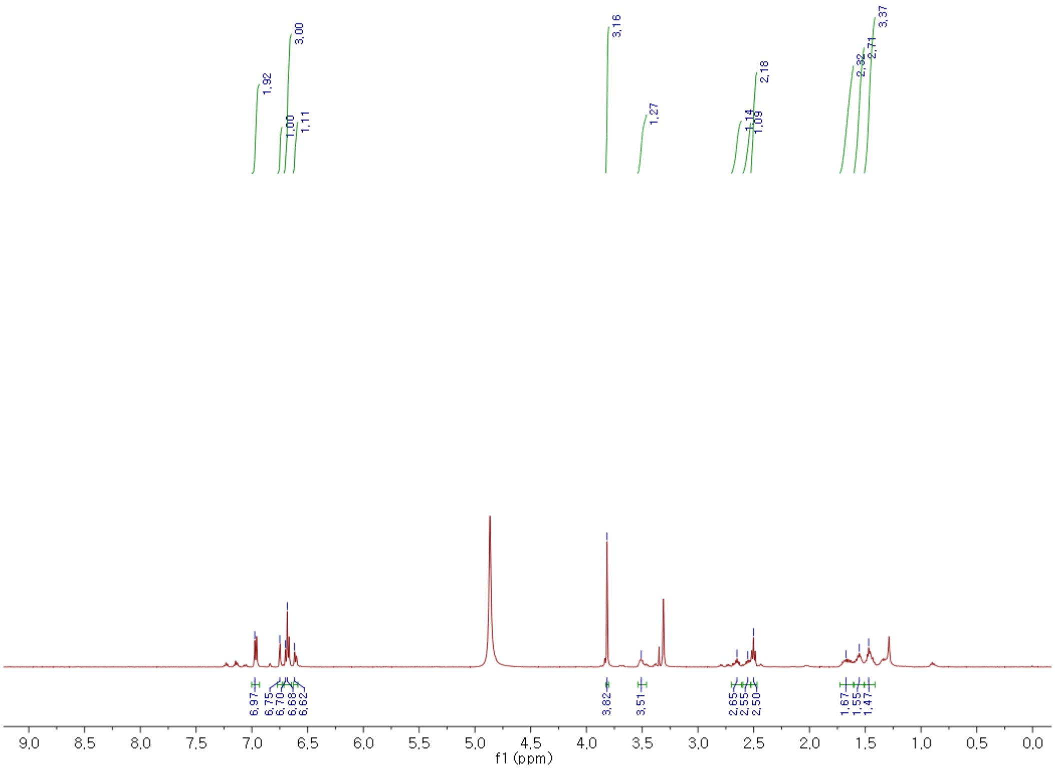

Figure S34. ^{1}H-NMR (CD_3OD, 500 MHz) spectrum of **5**.

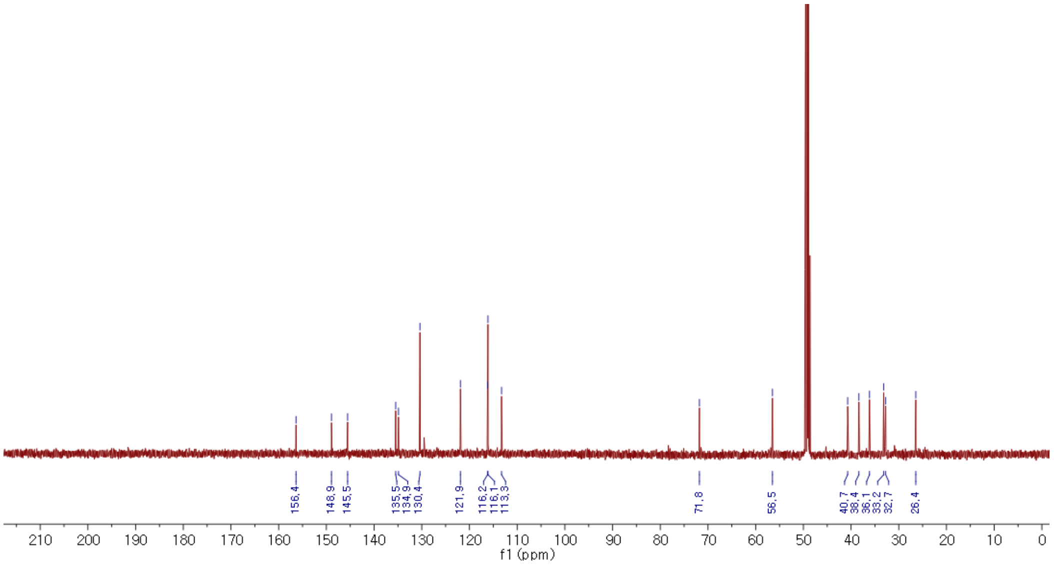

Figure S35. ^{13}C-NMR (CD_3OD, 125 MHz) spectrum of **5**.

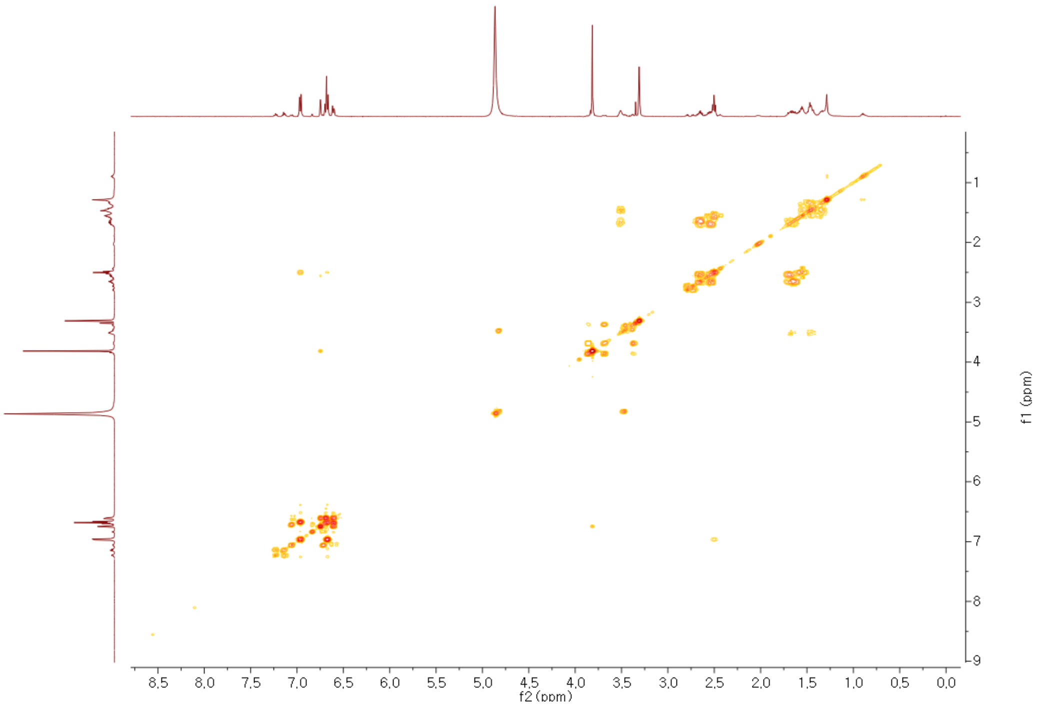

Figure S36. COSY spectrum of **5**.

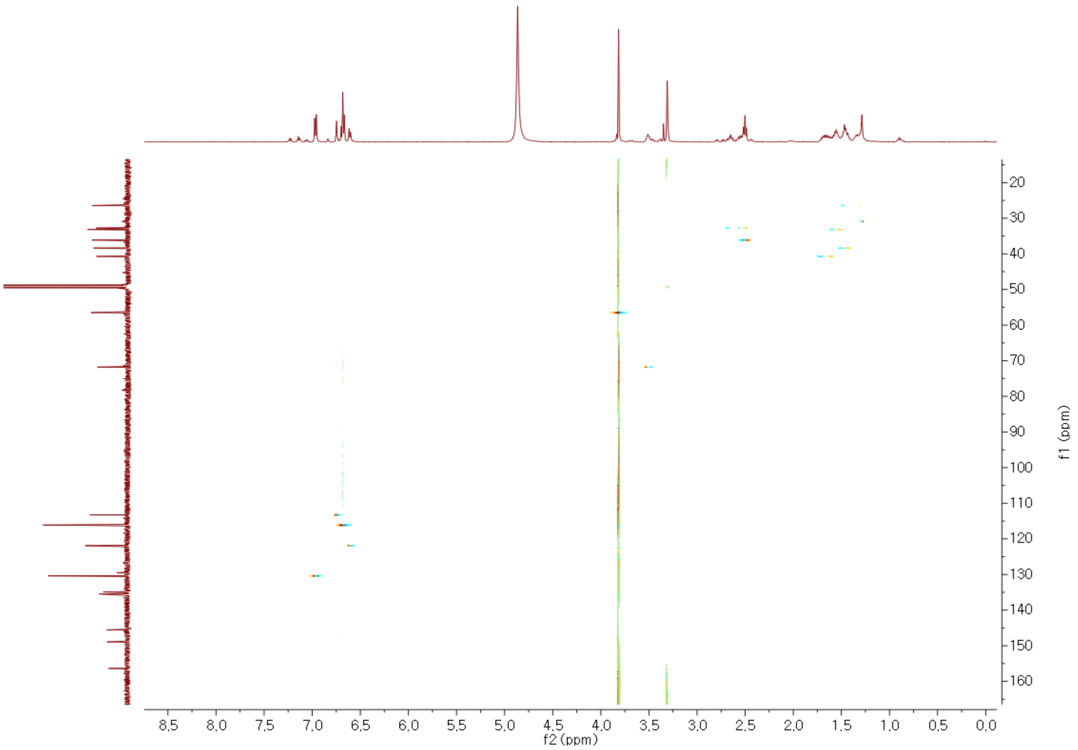

Figure S37. HSQC spectrum of **5**.

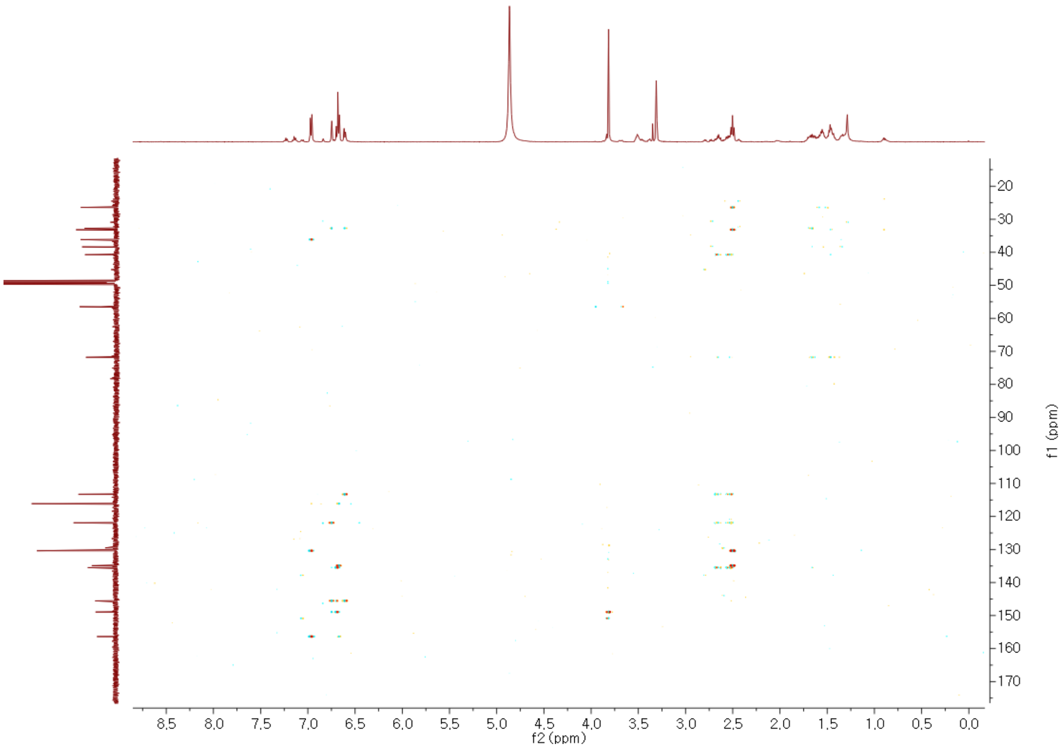

Figure S38. HMBC spectrum of **5**.

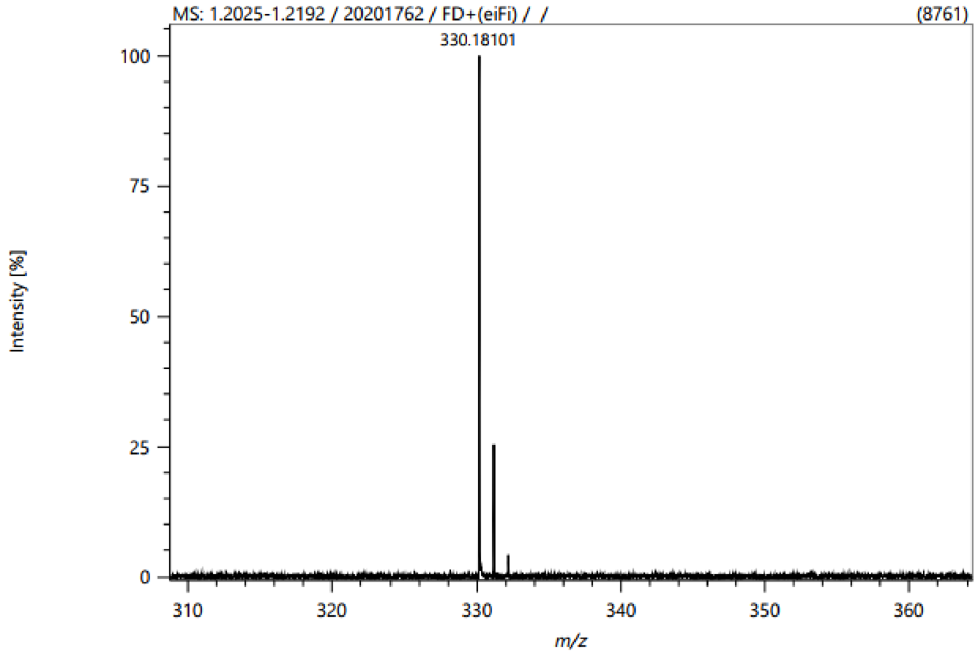

Elemental Composition

Parameters	
Tolerance:	30.00 mDa
Electron:	Odd/Even
Charge:	+1
DBE:	-90.0 - 90.0

Elements Set 1:

Symbol	C	H	O
Min	5	5	1
Max	20	26	4

Results

Mass	Intensity	Formula	Calculated Mass	Mass Difference [mDa]	Mass Difference [ppm]	DBE
330.18101	8760.60	C20 H26 O4	330.18256	-1.55	-4.69	8.0

Figure S39. HRFDMS spectrum of **5**.

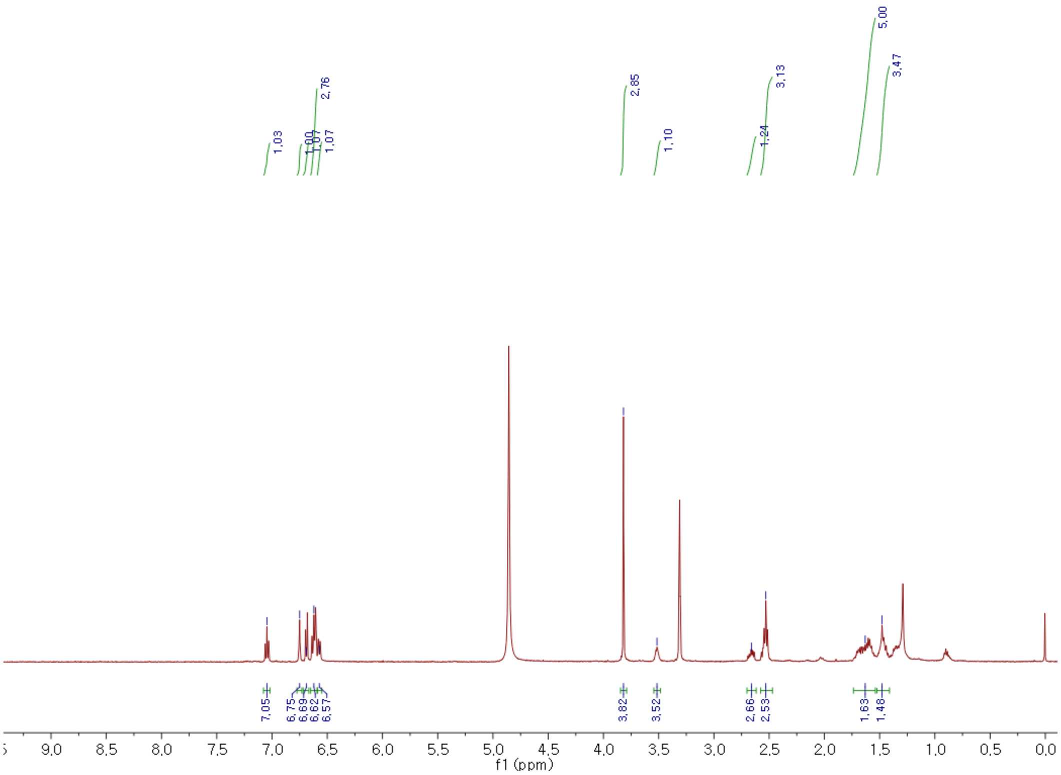

Figure S40. ^{1}H-NMR (CD_3OD, 500 MHz) spectrum of **6**.

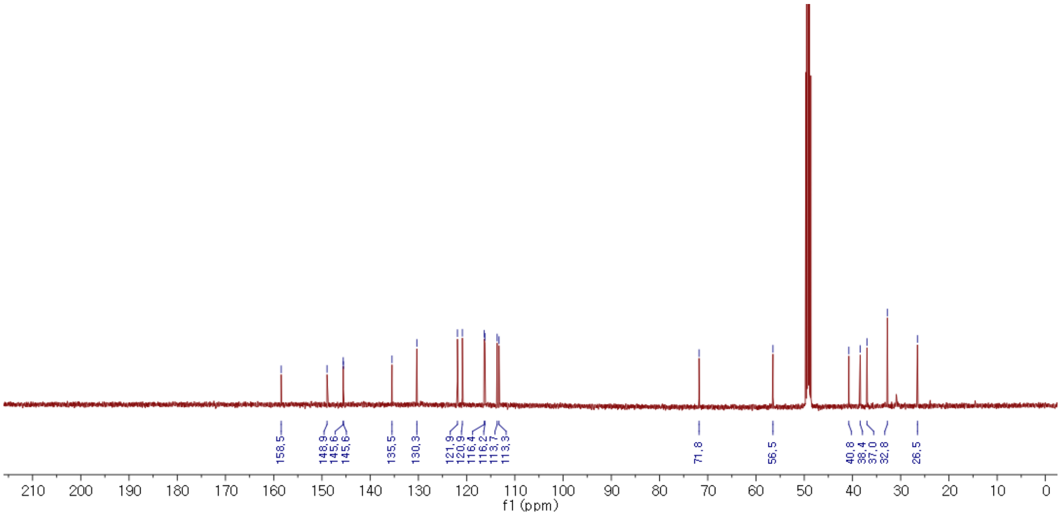

Figure S41. ^{13}C-NMR (CD_3OD, 125 MHz) spectrum of **6**.

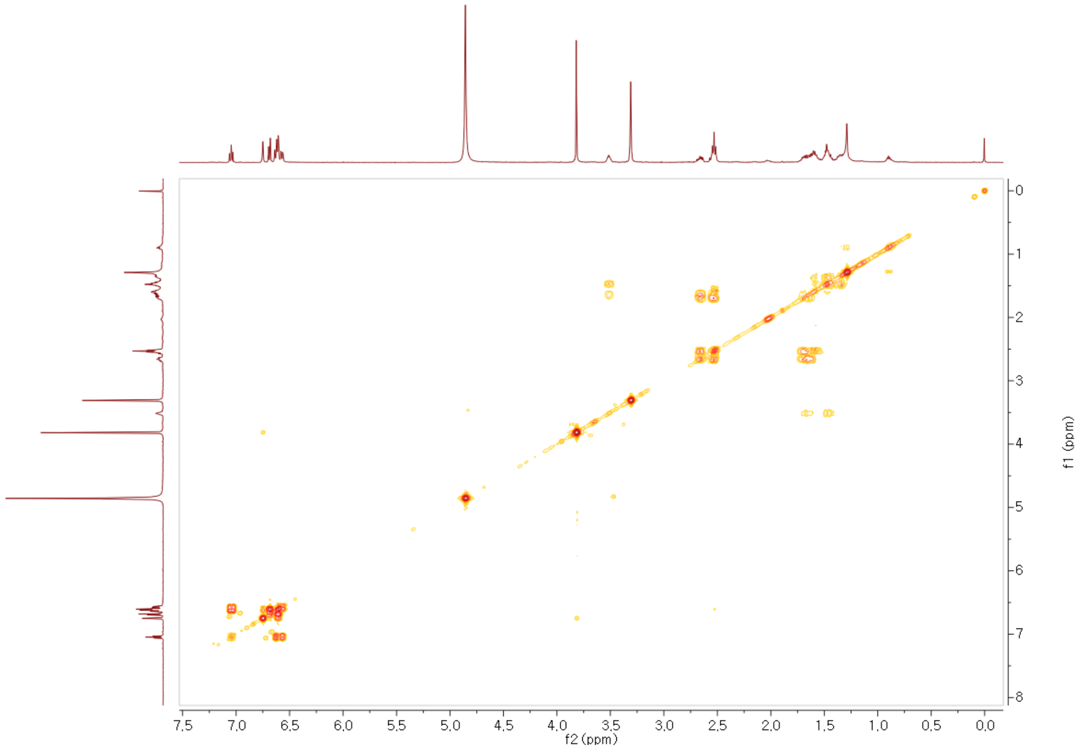

Figure S42. COSY spectrum of **6**.

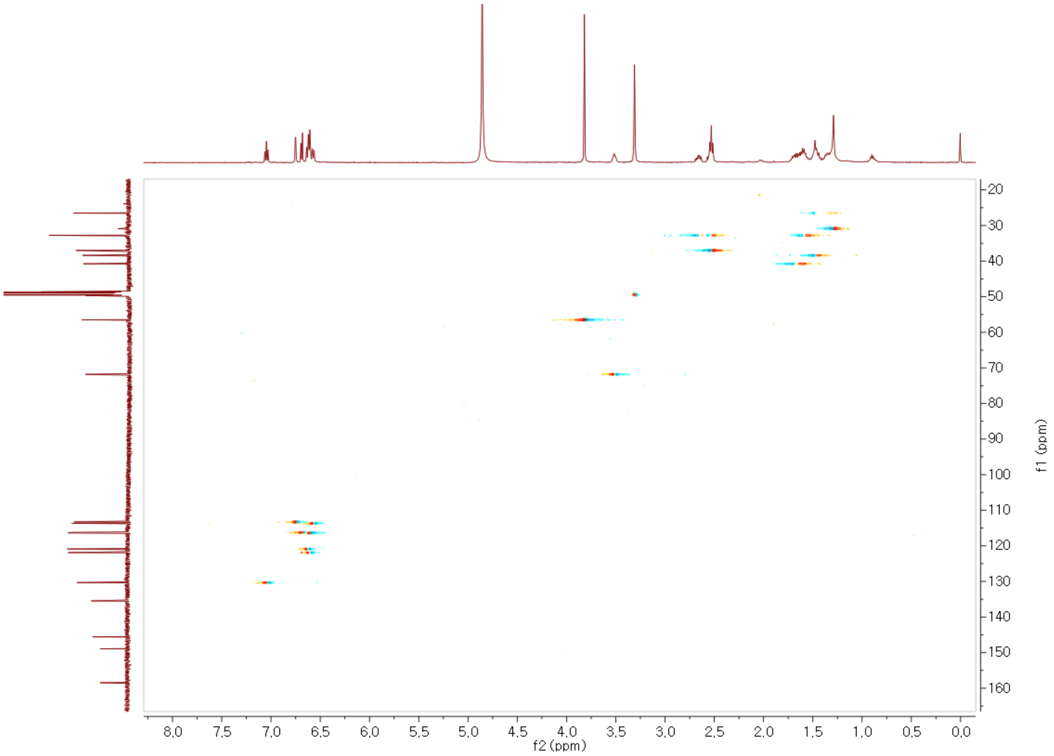

Figure S43. HSQC spectrum of **6**.

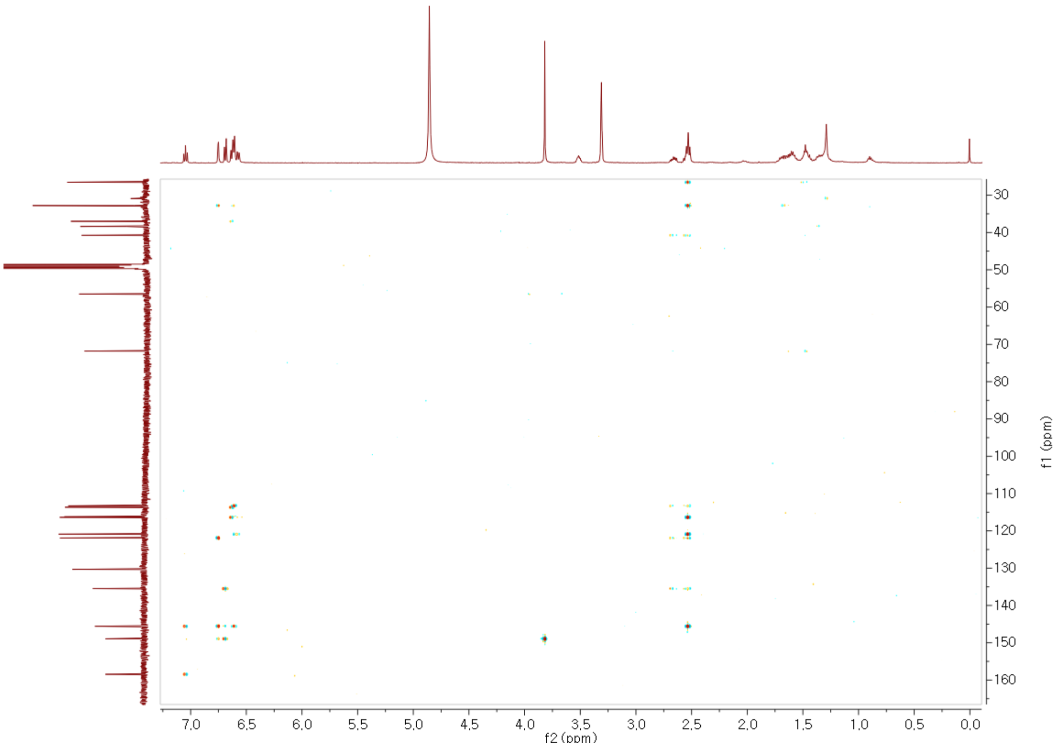

Figure S44. HMBC spectrum of **6**.

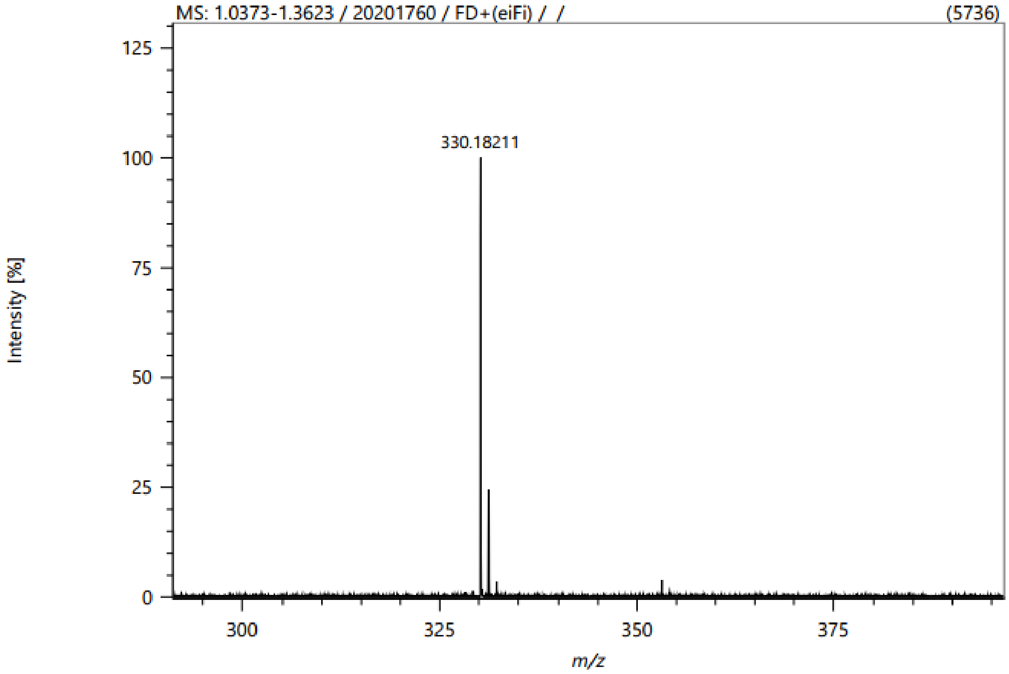

Elemental Composition

Parameters

Tolerance:	30.00 mDa
Electron:	Odd/Even
Charge:	+1
DBE:	-90.0 - 90.0

Elements Set 1:

Symbol	C	H	O
Min	5	5	1
Max	20	26	4

Results

Mass	Intensity	Formula	Calculated Mass	Mass Difference [mDa]	Mass Difference [ppm]	DBE
330.18211	5736.45	C20 H26 O4	330.18256	-0.45	-1.36	8.0

Figure S45. HRFDMS spectrum of **6**.

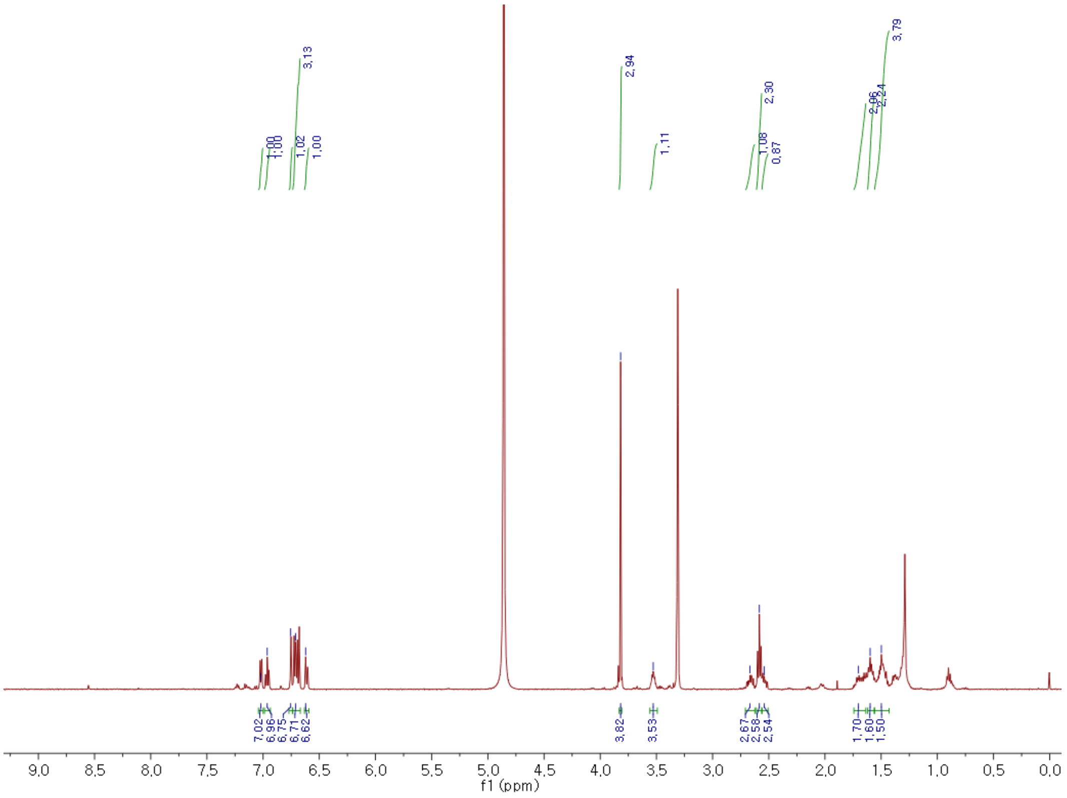

Figure S46. ^{1}H-NMR (CD_3OD, 500 MHz) spectrum of **7**.

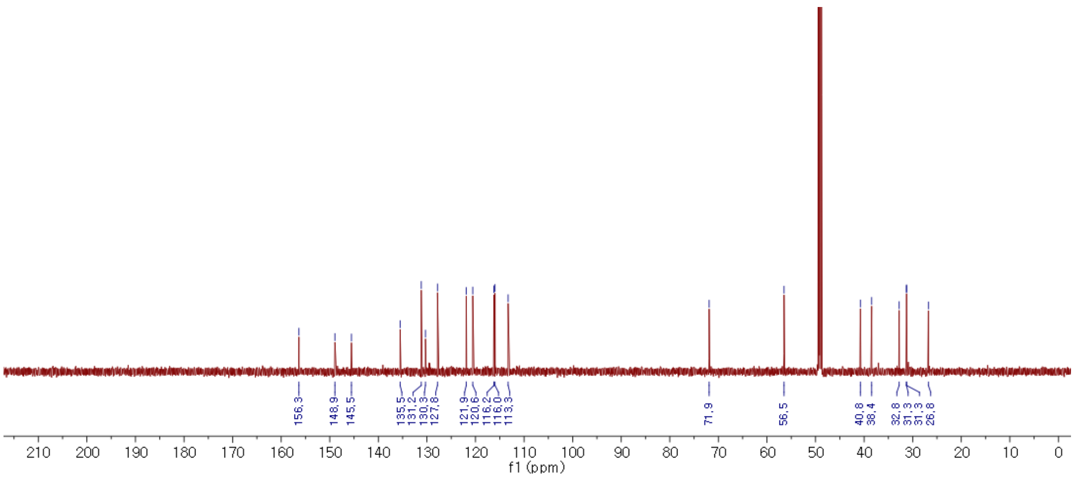

Figure S47. ^{13}C-NMR (CD_3OD, 125 MHz) spectrum of **7**.

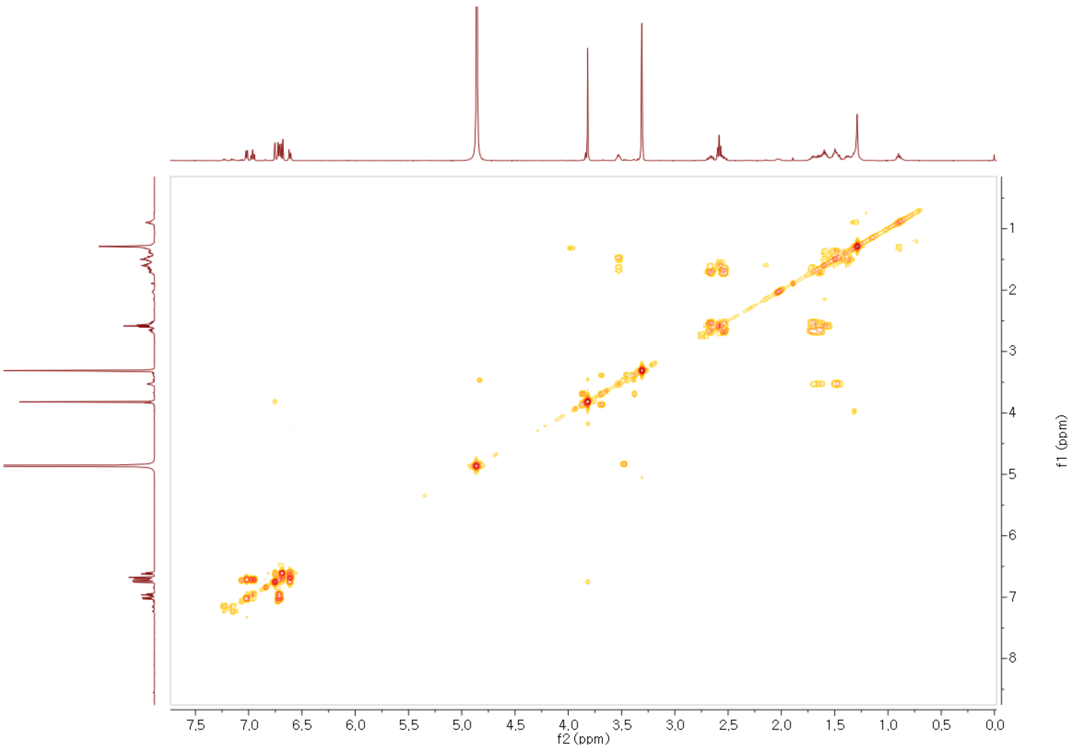

Figure S48. COSY spectrum of **7**.

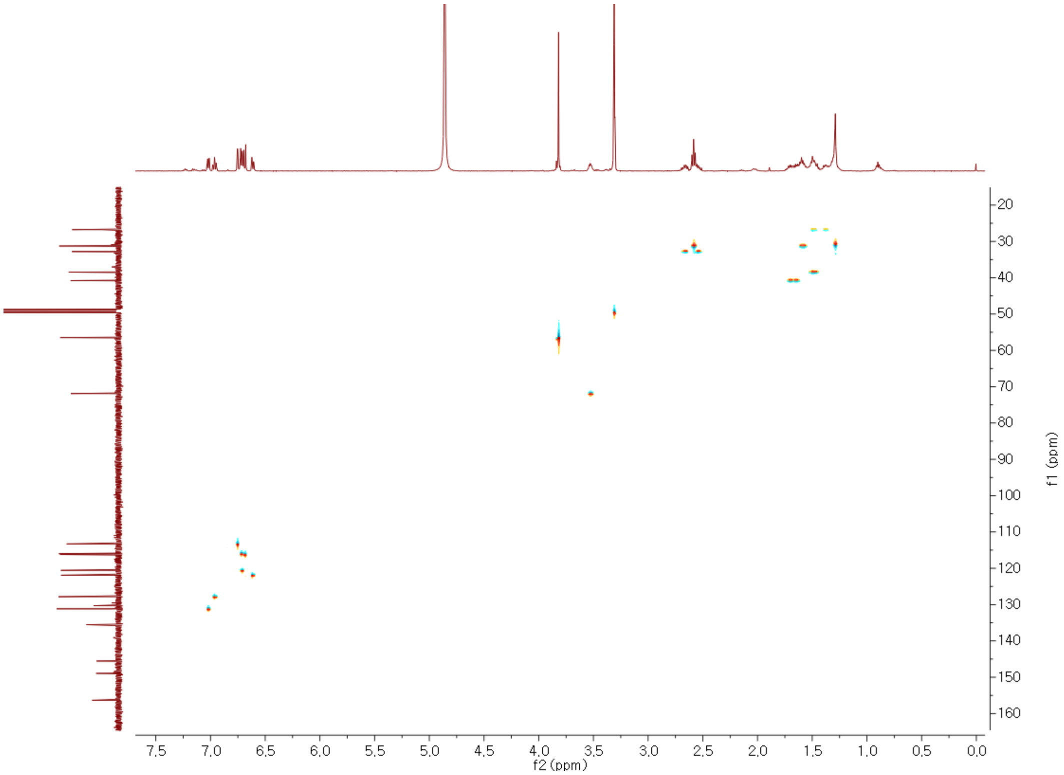

Figure S49. HSQC spectrum of **7**.

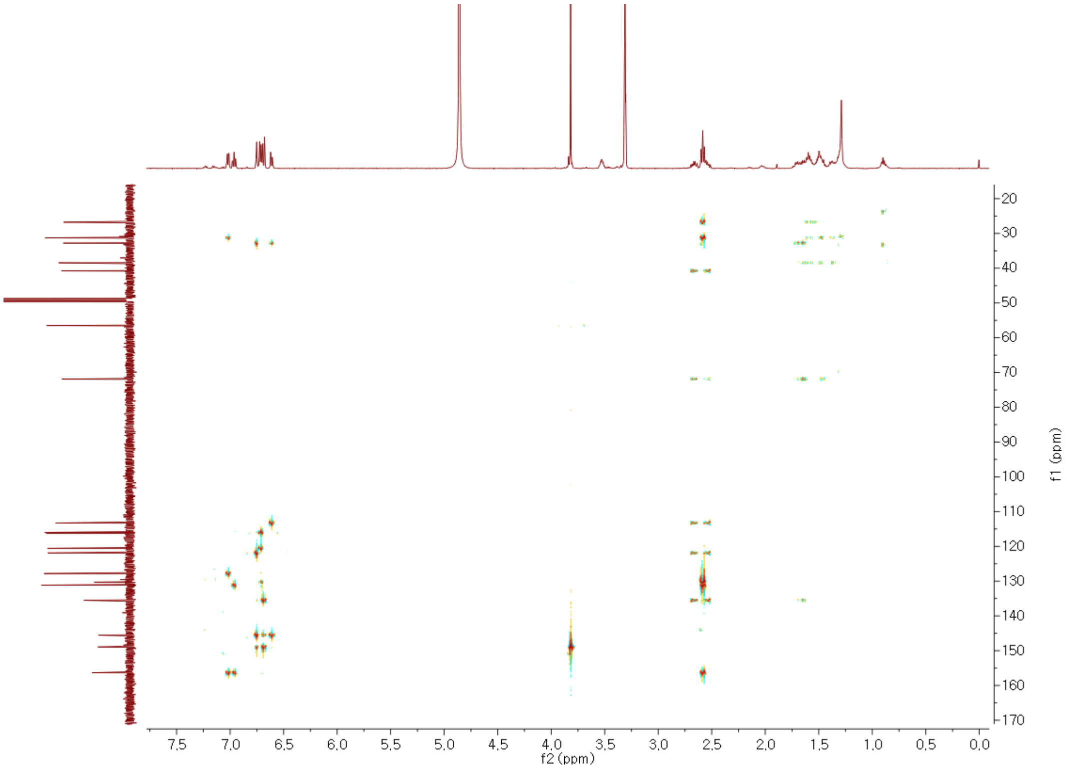

Figure S50. HMBC spectrum of **7**.

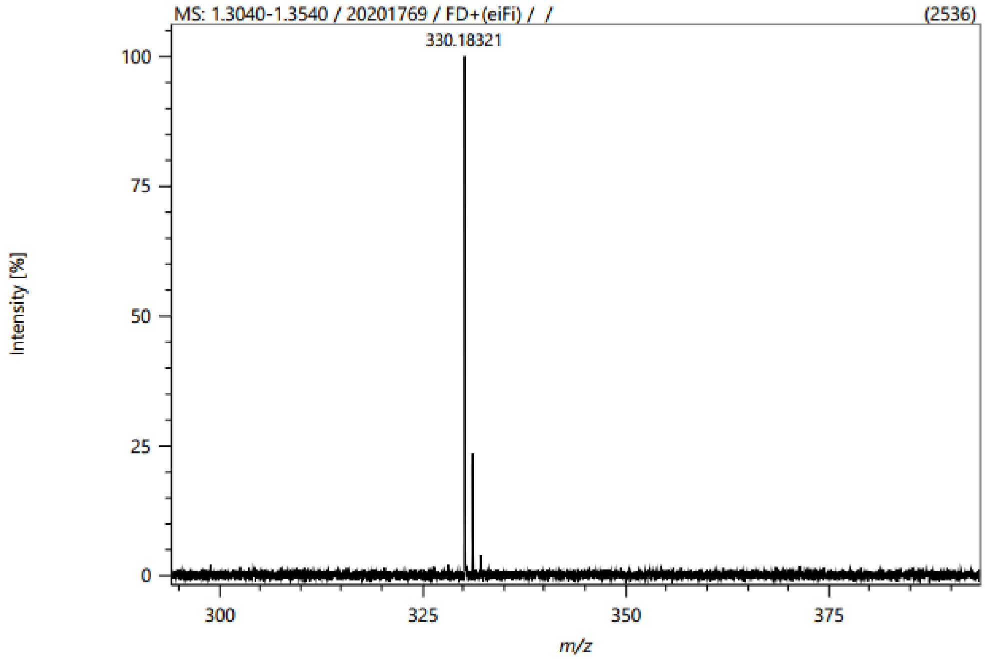

Elemental Composition

Parameters

Tolerance:	30.00 mDa
Electron:	Odd/Even
Charge:	+1
DBE:	-90.0 - 90.0

Elements Set 1:

Symbol	C	H	O
Min	5	5	1
Max	20	26	4

Results

Mass	Intensity	Formula	Calculated Mass	Mass Difference [mDa]	Mass Difference [ppm]	DBE
330.18321	2535.95	C20 H26 O4	330.18256	0.65	1.97	8.0

Figure S51. HRFDMS spectrum of **7**.

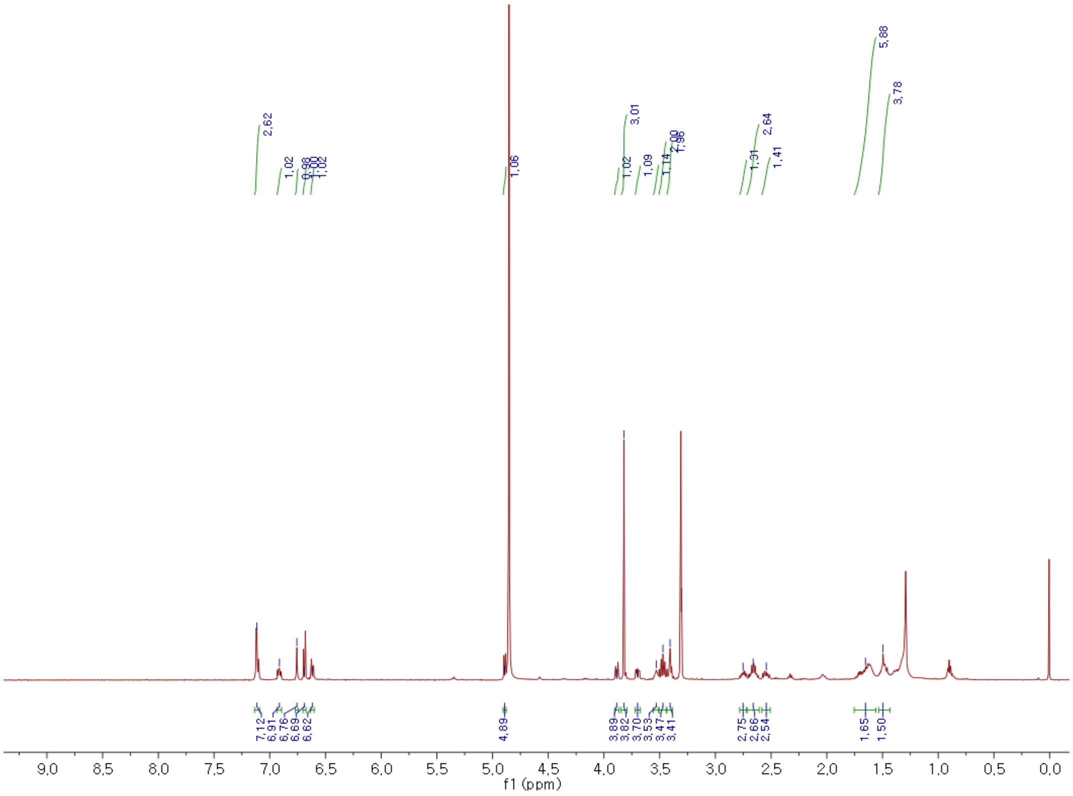

Figure S52. ^{1}H-NMR (CD_3OD, 500 MHz) spectrum of **8**.

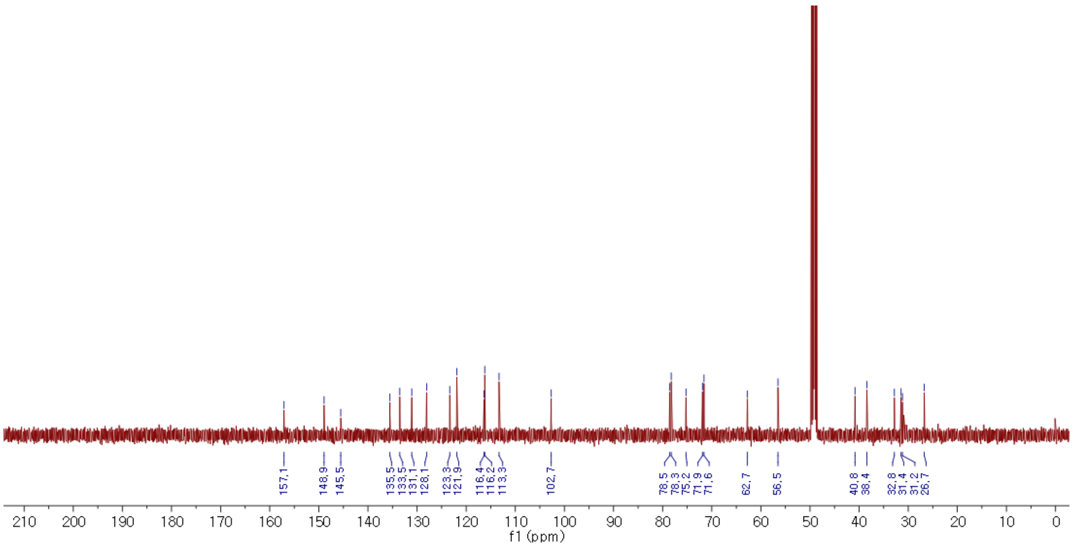

Figure S53. ^{13}C-NMR (CD_3OD, 125 MHz) spectrum of **8**.

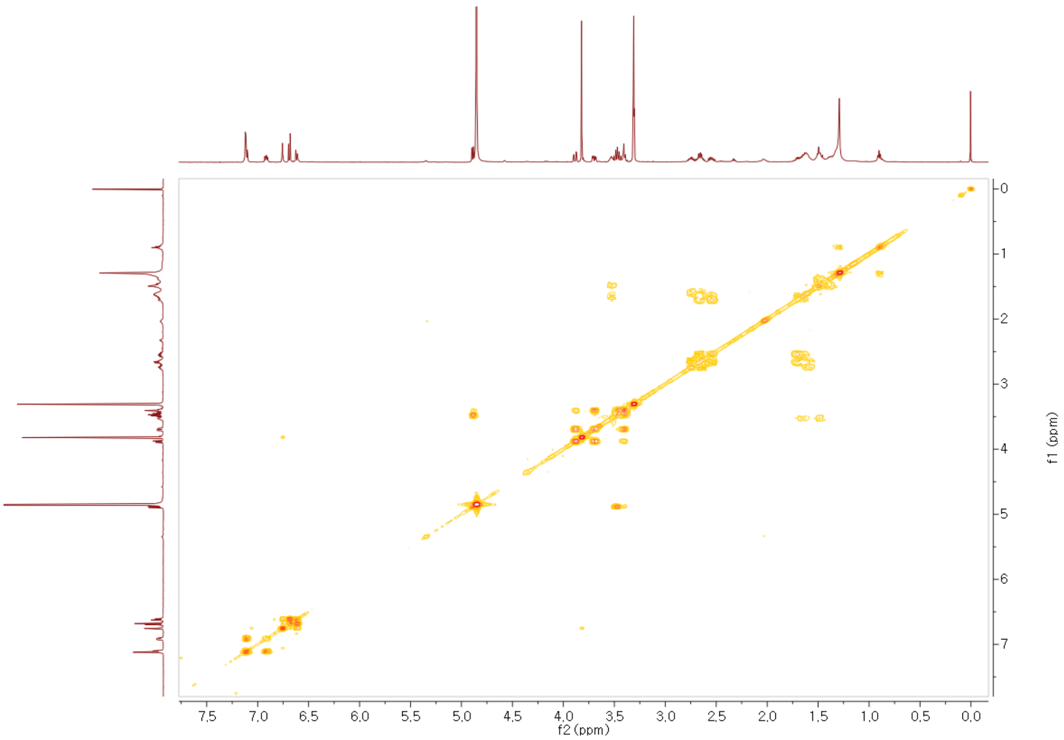

Figure S54. COSY spectrum of **8**.

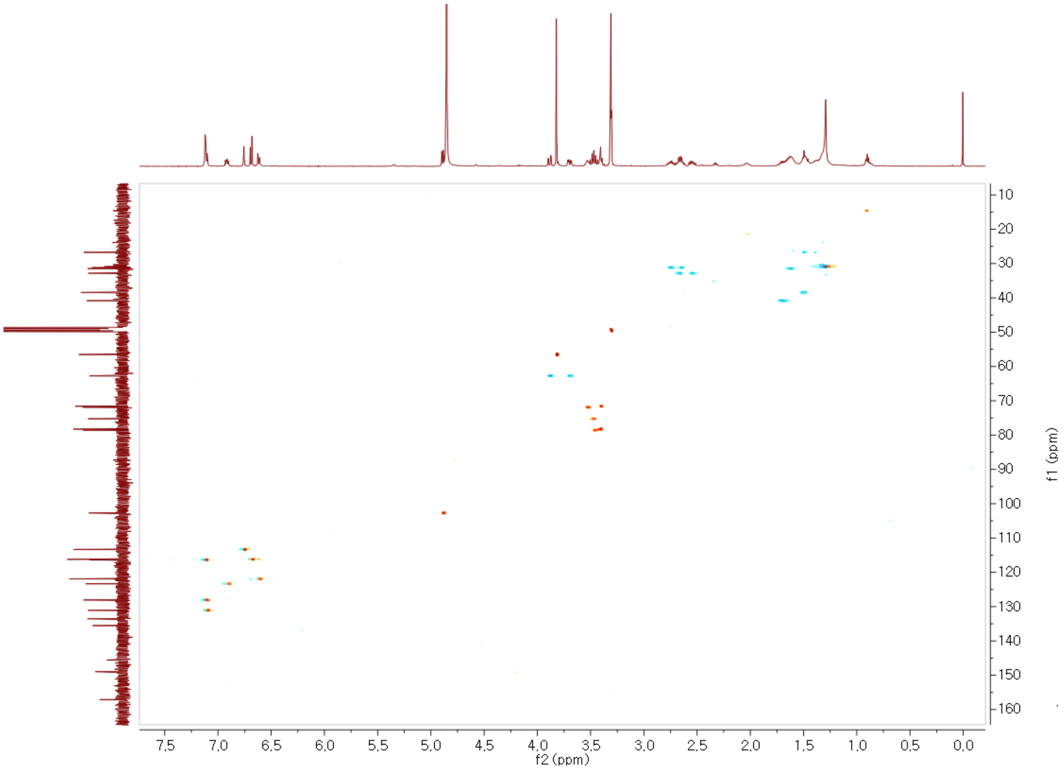

Figure S55. HSQC spectrum of **8**.

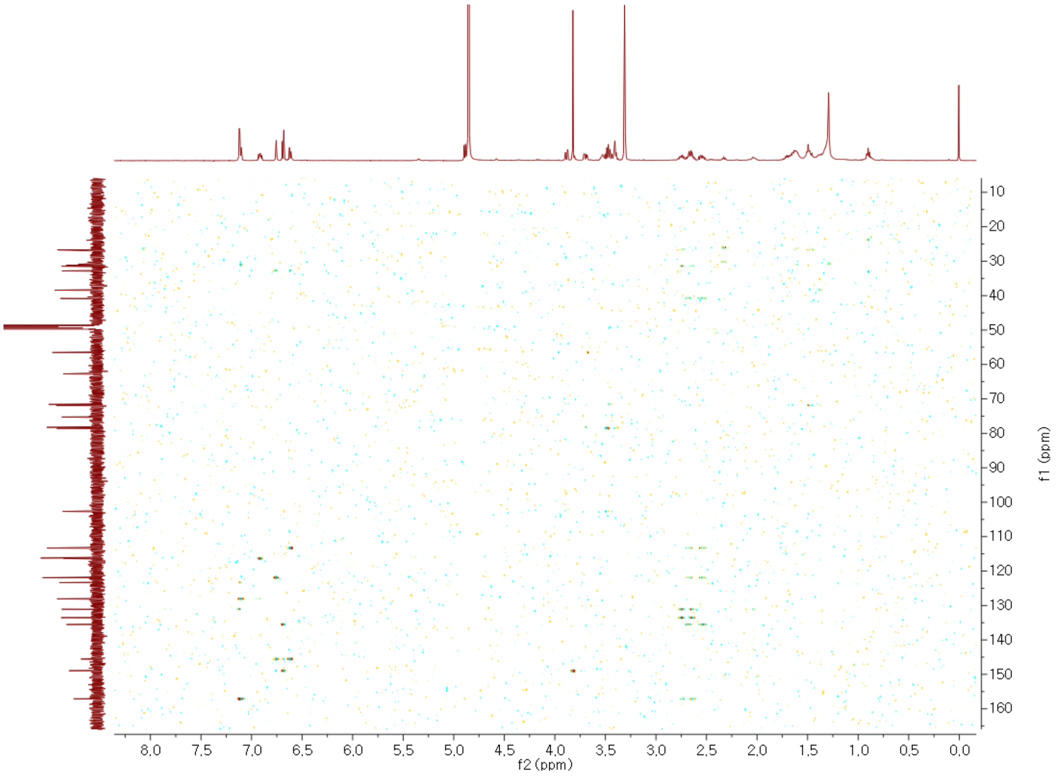

Figure S56. HMBC spectrum of **8**.

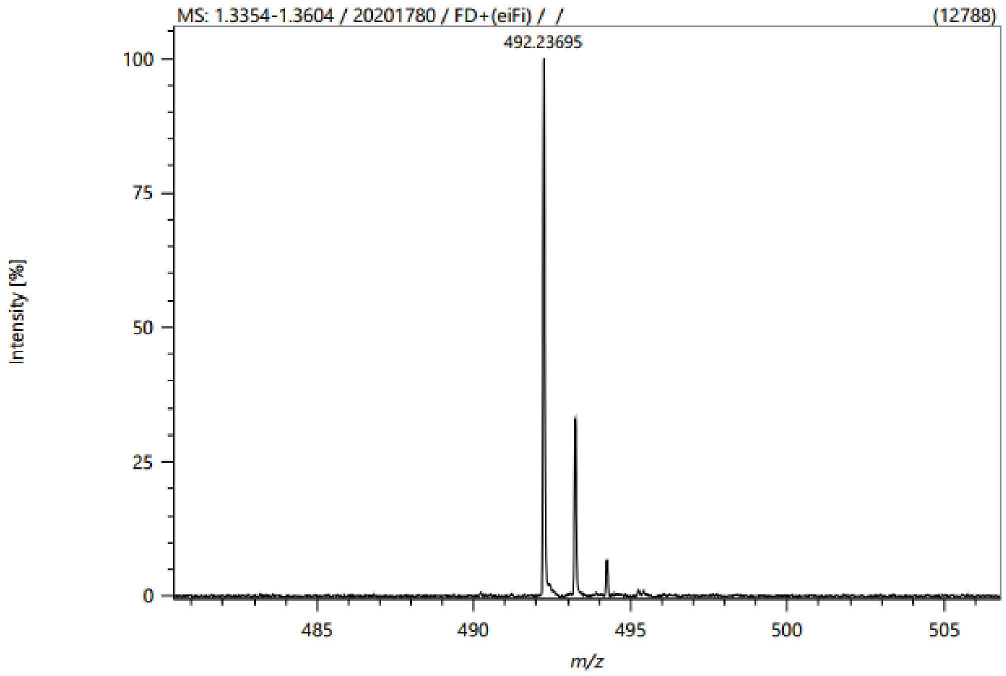

Elemental Composition

Parameters	
Tolerance:	30.00 mDa
Electron:	Odd/Even
Charge:	+1
DBE:	-90.0 - 90.0

Elements Set 1:

Symbol	C	H	O
Min	5	5	1
Max	26	36	9

Results

Mass	Intensity	Formula	Calculated Mass	Mass Difference [mDa]	Mass Difference [ppm]	DBE
492.23695	12787.86	C26 H36 O9	492.23538	1.56	3.17	9.0

Figure S57. HRFDMS spectrum of **8**.

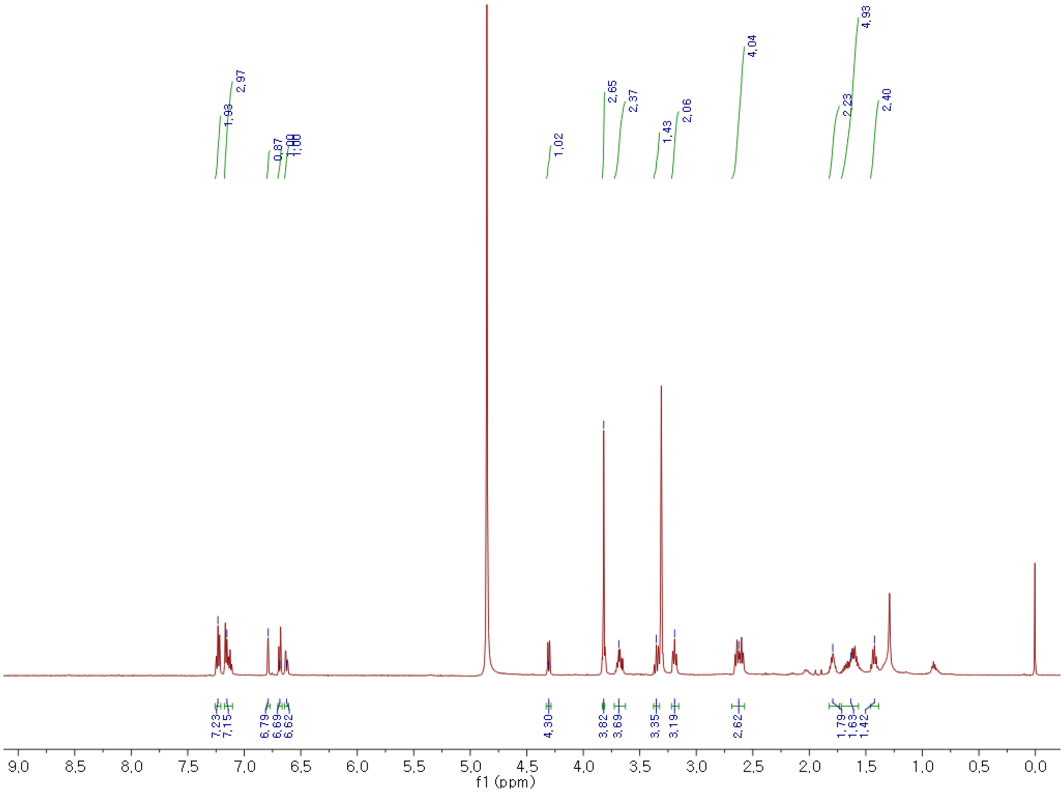

Figure S58. ^{1}H-NMR (CD_3OD, 500 MHz) spectrum of **9**.

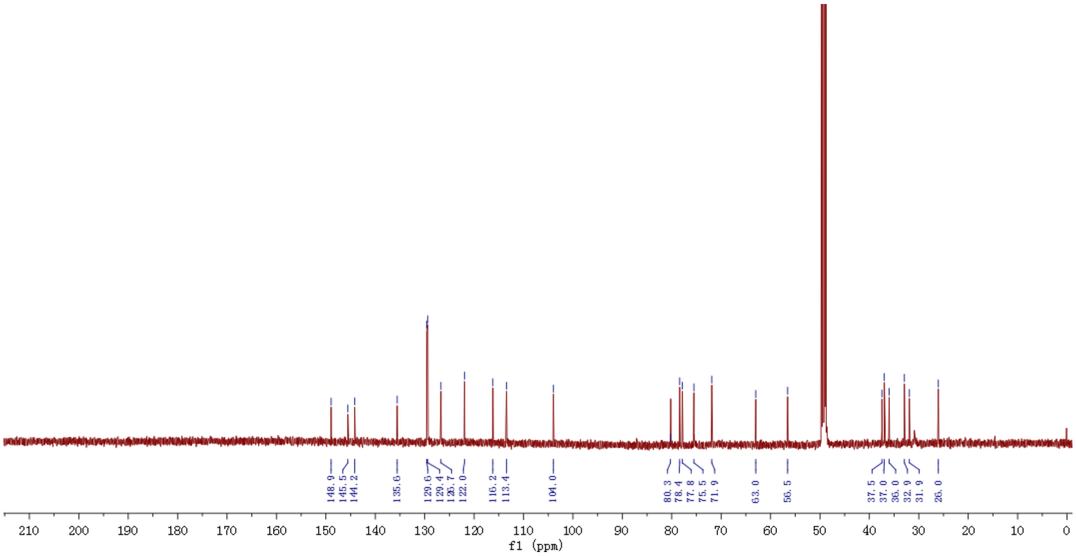

Figure S59. ^{13}C-NMR (CD_3OD, 125 MHz) spectrum of **9**.

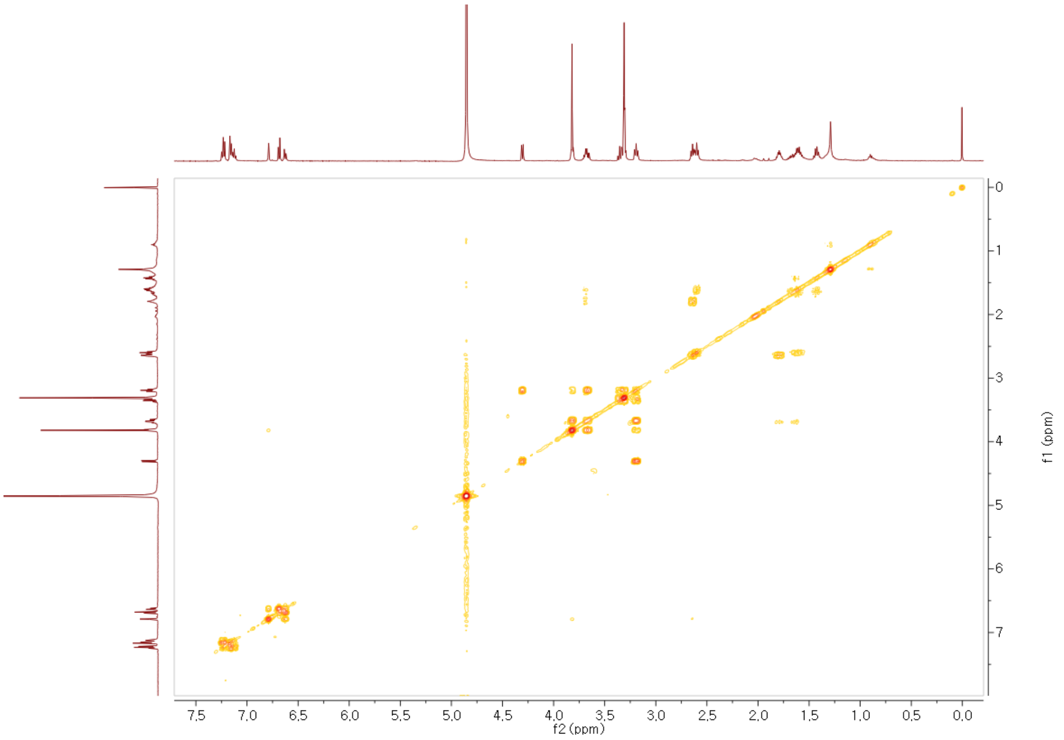

Figure S60. COSY spectrum of **9**.

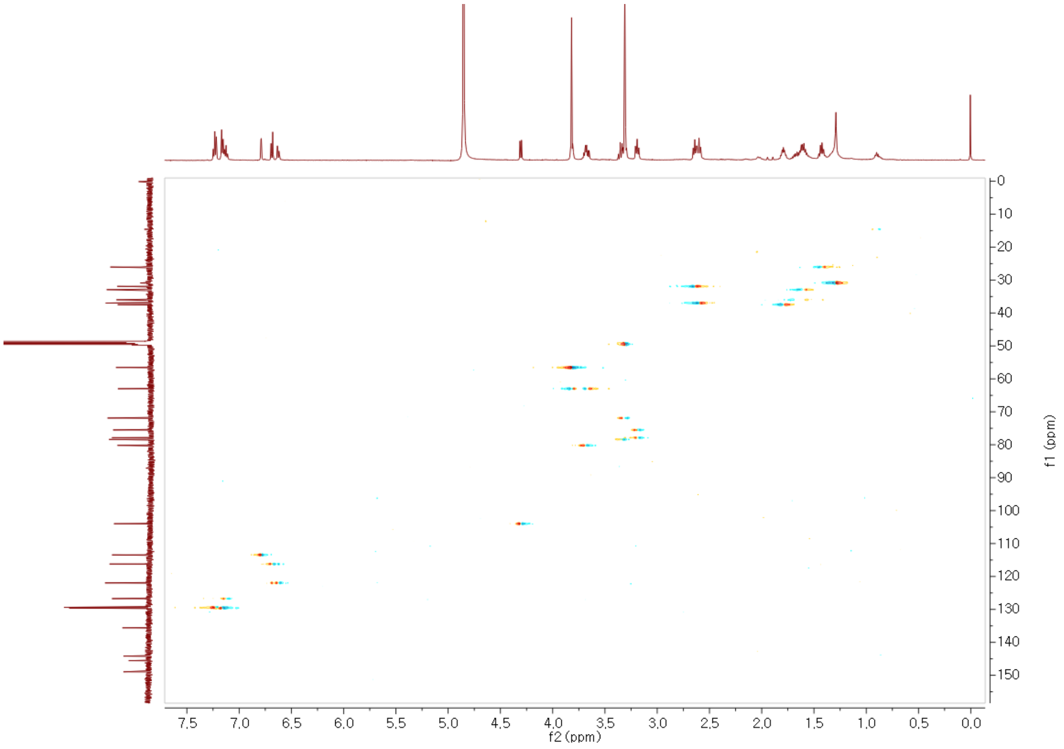

Figure S61. HSQC spectrum of **9**.

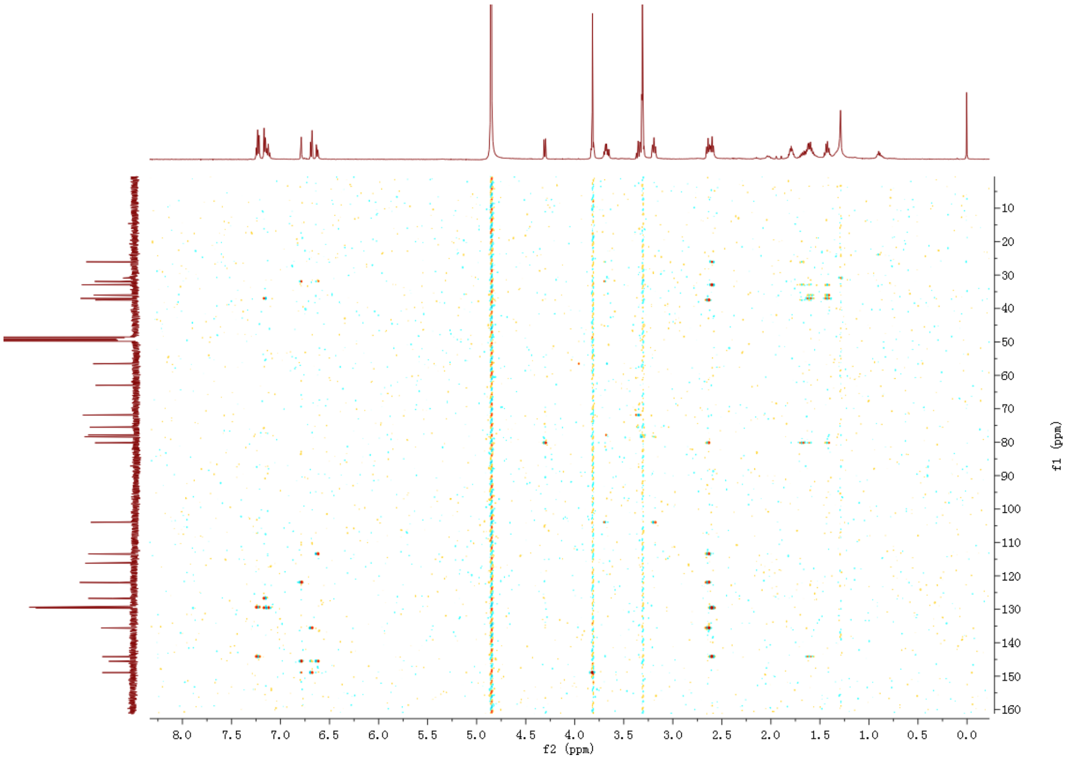

Figure S62. HMBC spectrum of **9**.

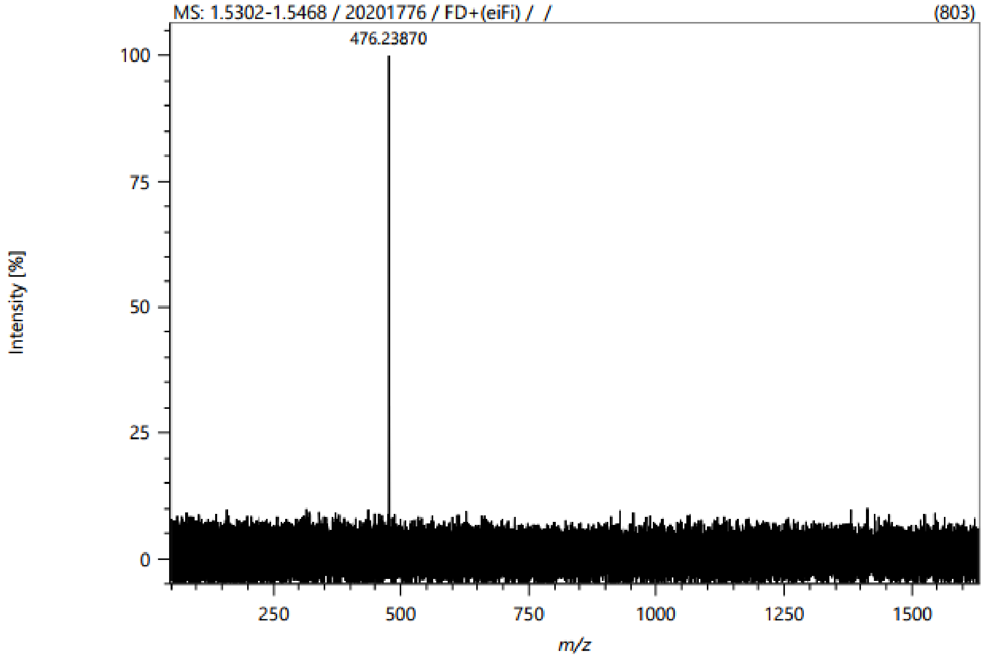

Elemental Composition

Parameters	
Tolerance:	30.00 mDa
Electron:	Odd/Even
Charge:	+1
DBE:	-90.0 - 90.0

Elements Set 1:

Symbol	C	H	O
Min	5	5	1
Max	26	36	8

Results

Mass	Intensity	Formula	Calculated Mass	Mass Difference [mDa]	Mass Difference [ppm]	DBE
476.23870	802.98	C26 H36 O8	476.24047	-1.77	-3.72	9.0

Figure S63. HRFDMS spectrum of **9**.

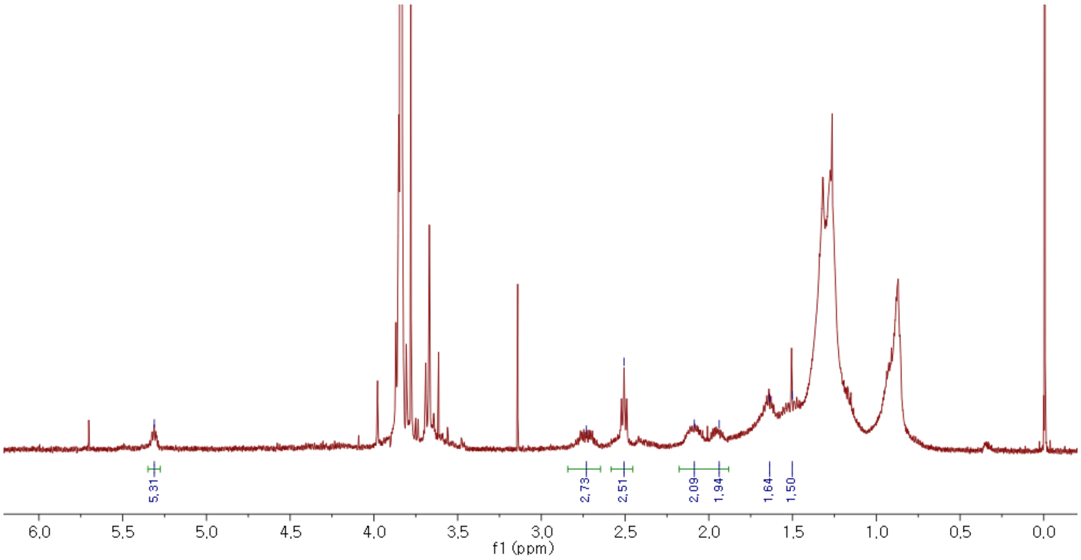

Figure S64. ^{1}H-NMR (pyridine-d_5, 500 MHz) spectrum of (*S*)-MTPA ester of **2**.

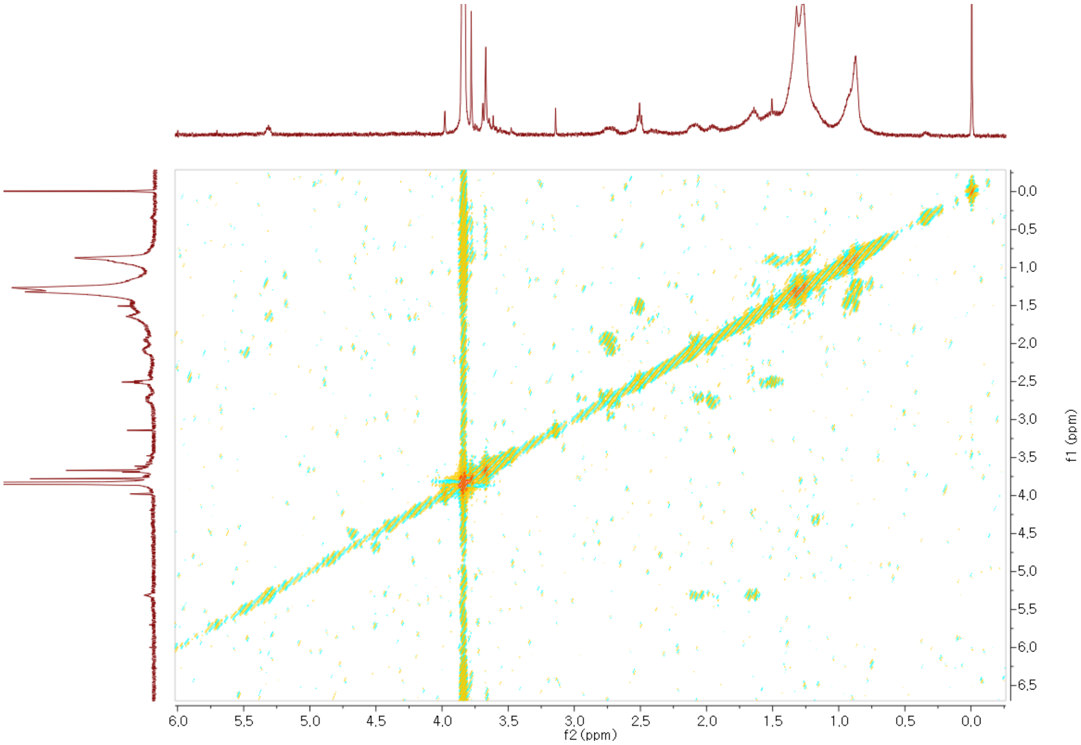

Figure S65. COSY spectrum of (*S*)-MTPA ester of **2**.

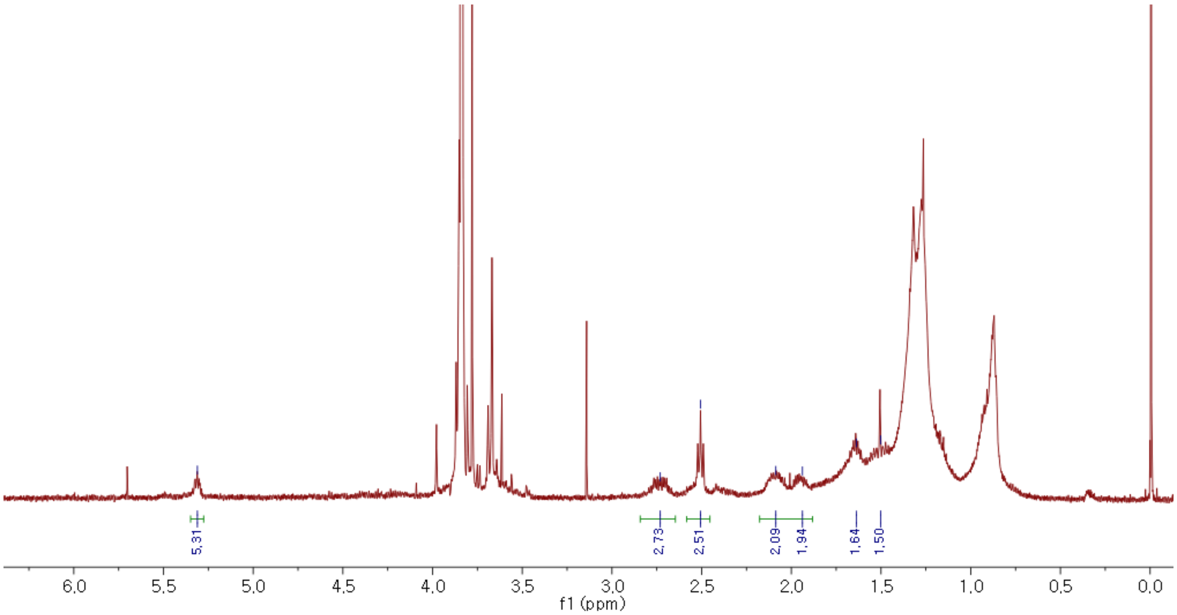

Figure S66. ^{1}H-NMR (pyridine-d_5, 500 MHz) spectrum of (*R*)-MTPA ester of **2**.

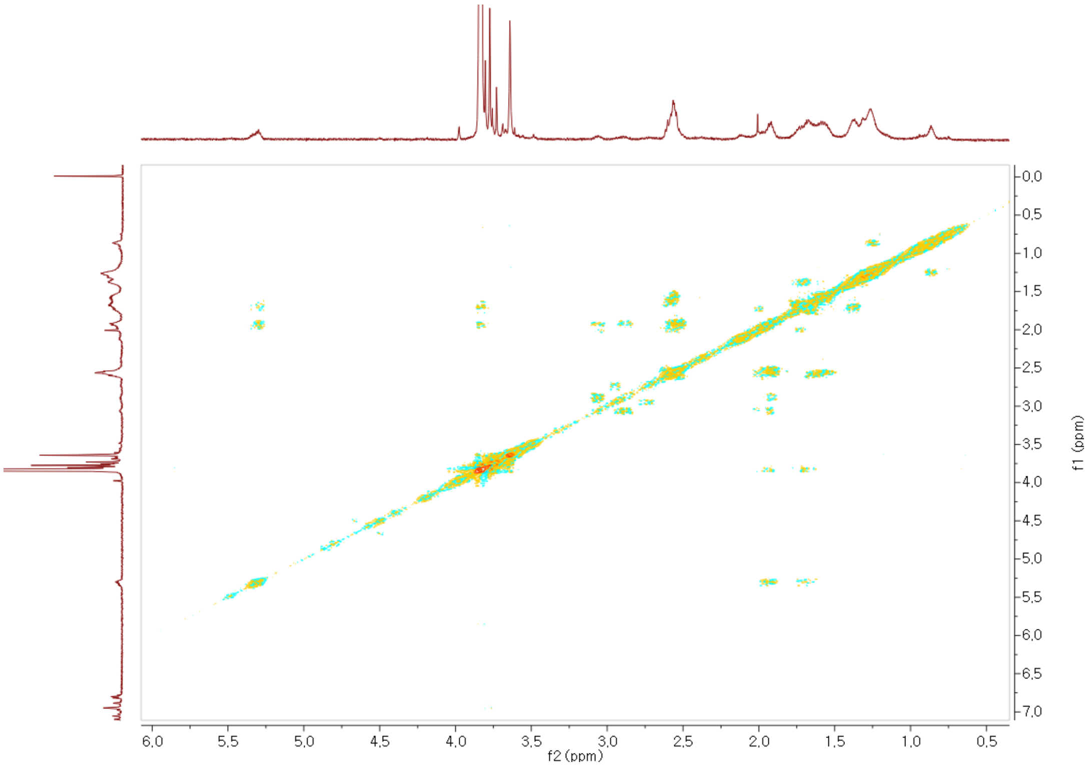

Figure S67. COSY spectrum of (*R*)-MTPA ester of **2**.

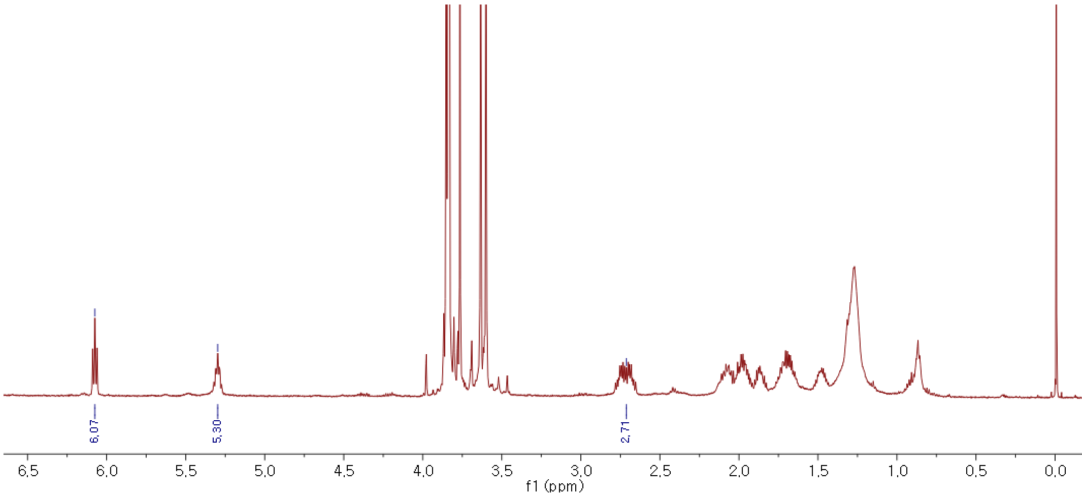

Figure S68. ^{1}H-NMR (pyridine-d_5, 500 MHz) spectrum of (*S*)-MTPA ester of **3a**.

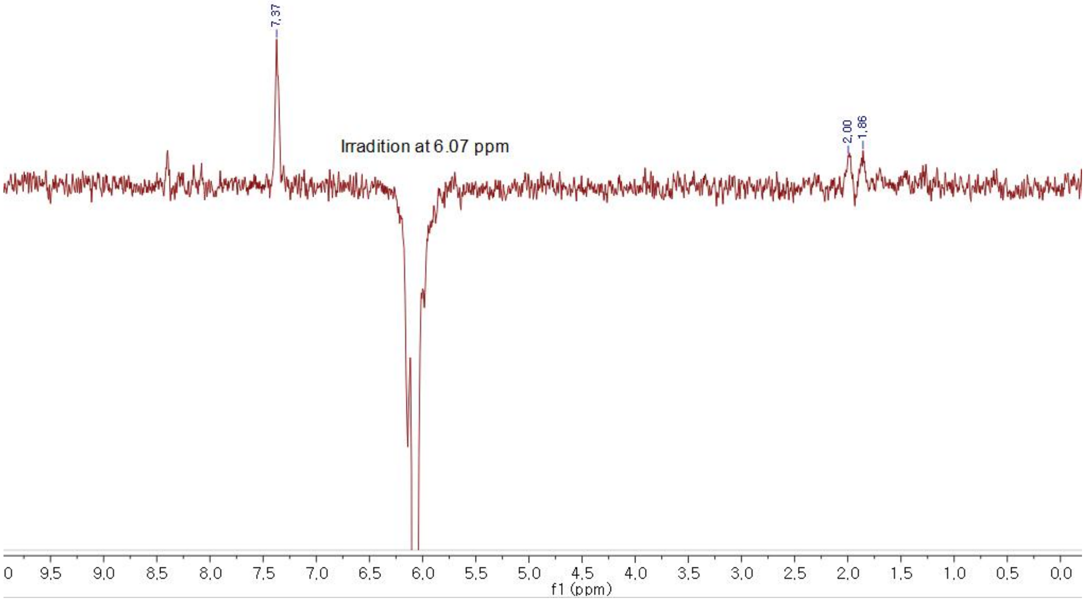

Figure S69. NOE spectrum of (*S*)-MTPA ester of **3a**.

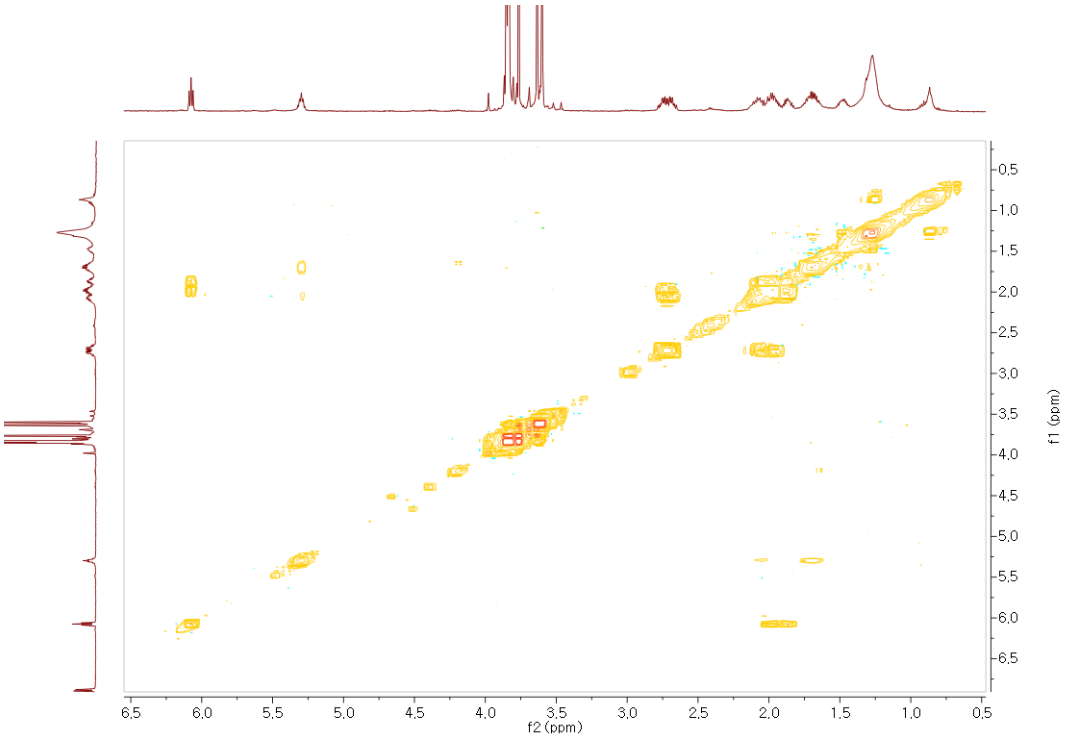

Figure S70. COSY spectrum of (*S*)-MTPA ester of **3a**.

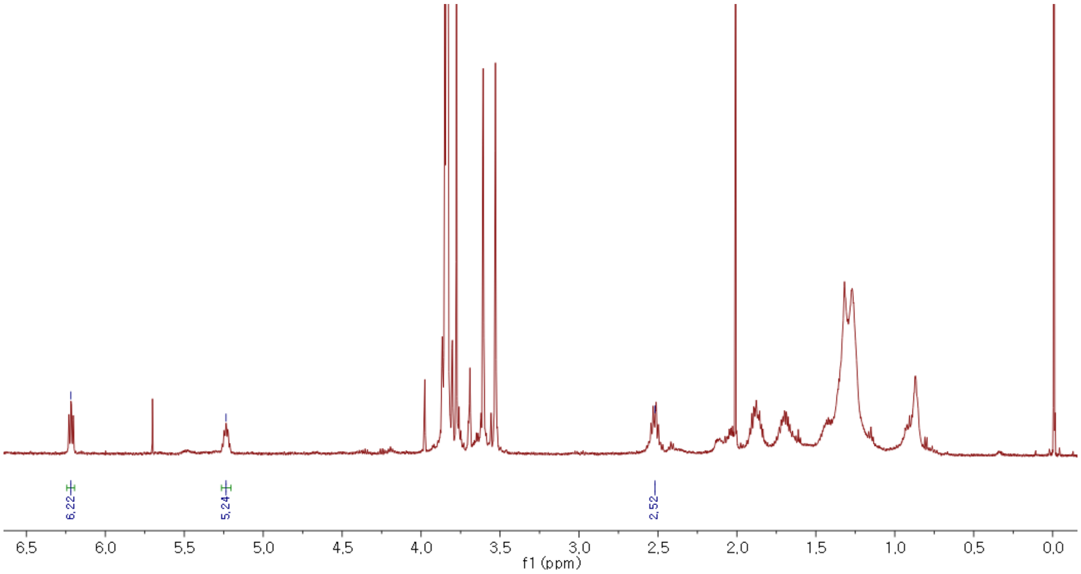

Figure S71. ^{1}H-NMR (pyridine-d_5, 500 MHz) spectrum of (*R*)-MTPA ester of **3a**.

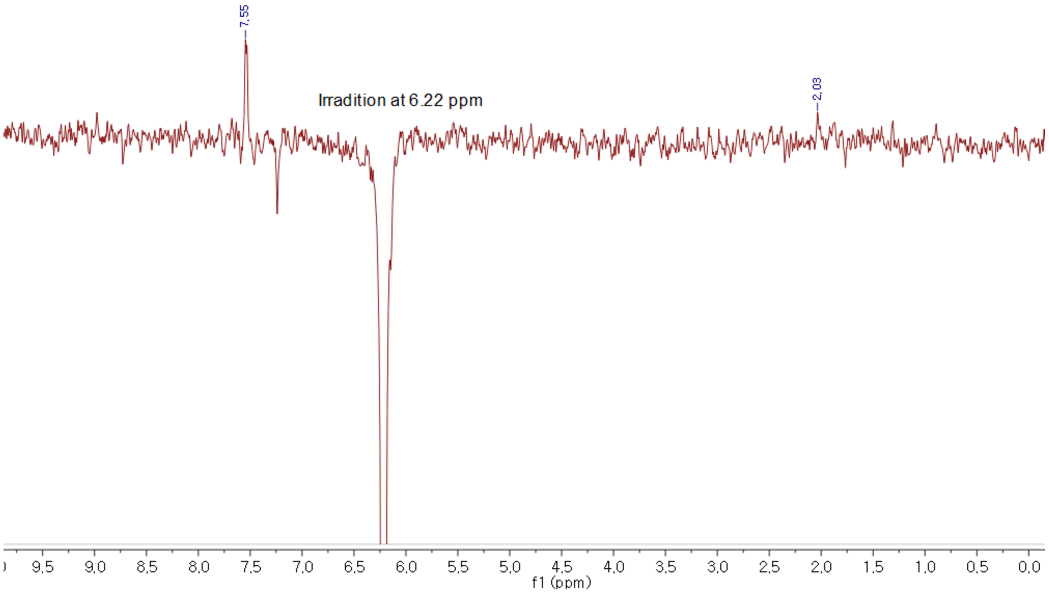

Figure S72. NOE spectrum of (*R*)-MTPA ester of **3a**.

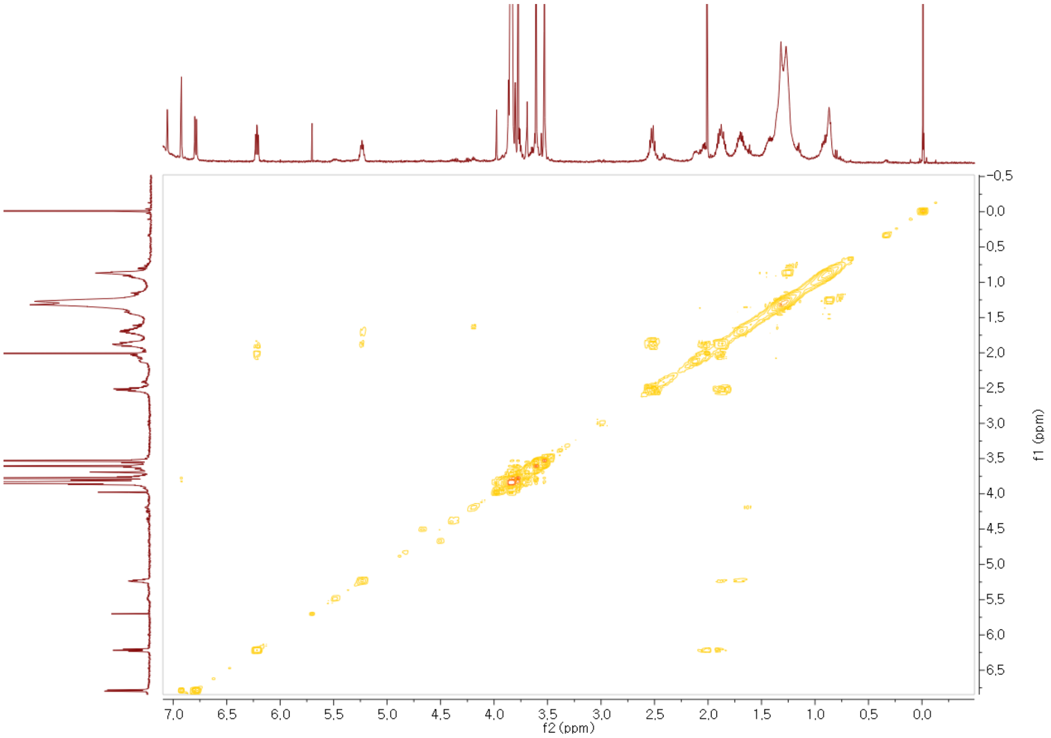

Figure S73. COSY spectrum of (*R*)-MTPA ester of **3a**.

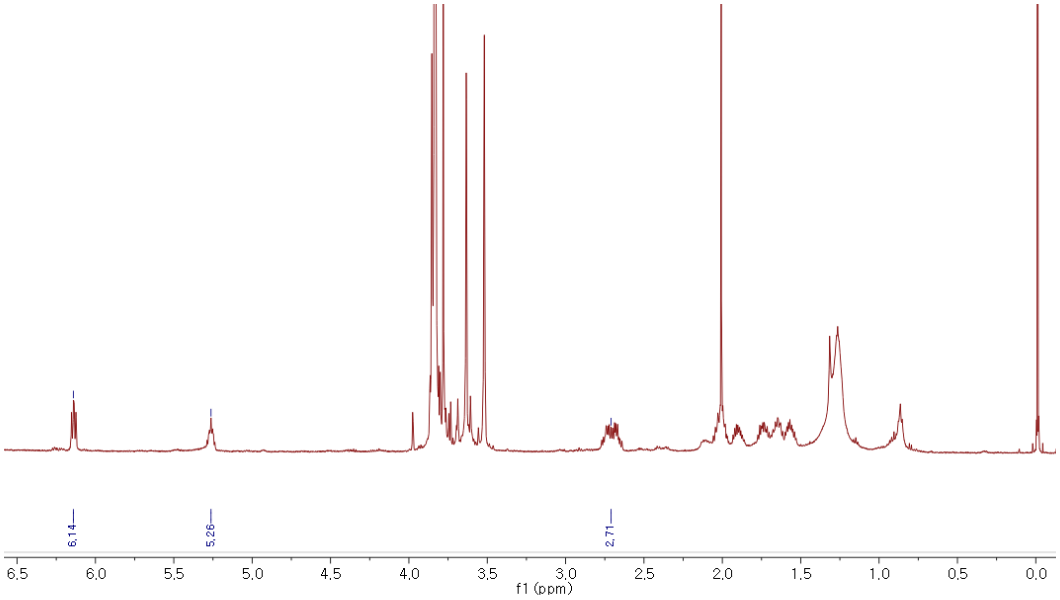

Figure S74. ^{1}H-NMR (pyridine-d_5, 500 MHz) spectrum of (*S*)-MTPA ester of **3b**.

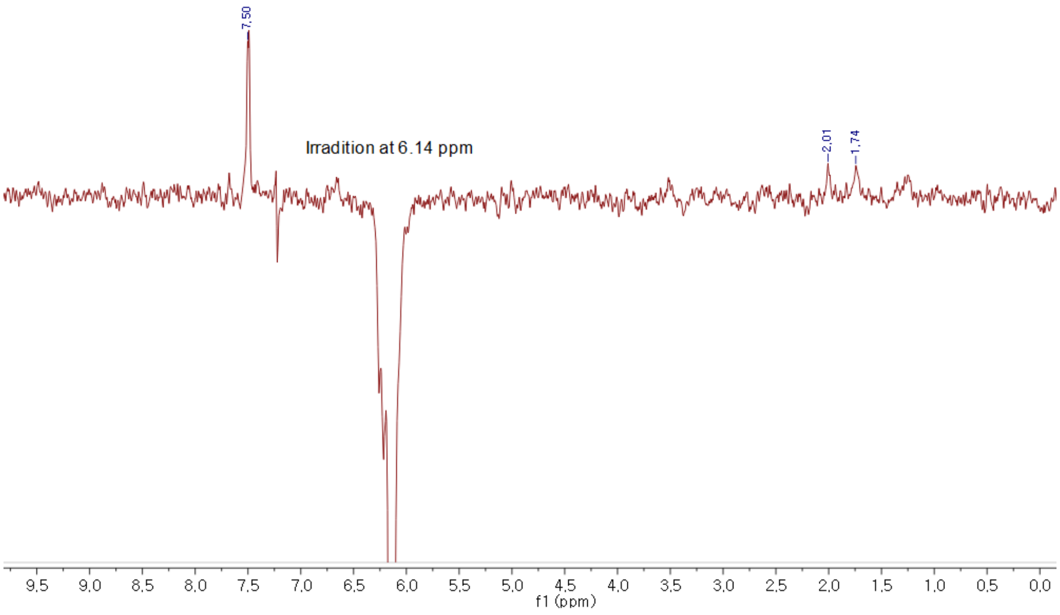

Figure S75. NOE spectrum of (*S*)-MTPA ester of **3b**.

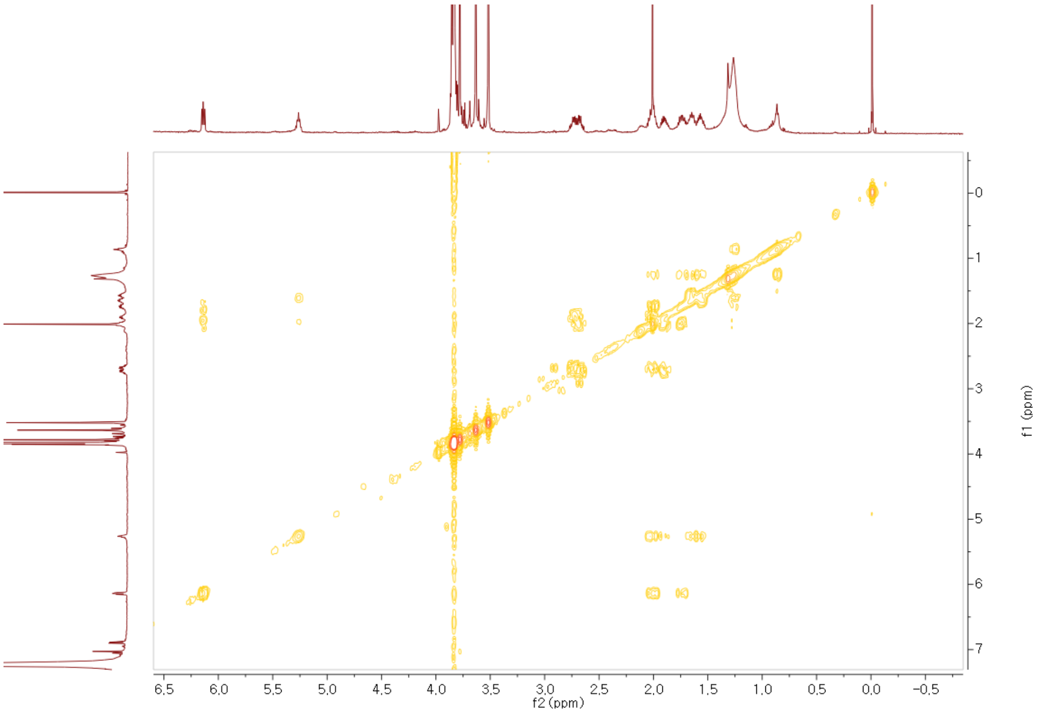

Figure S76. COSY spectrum of (*S*)-MTPA ester of **3b**.

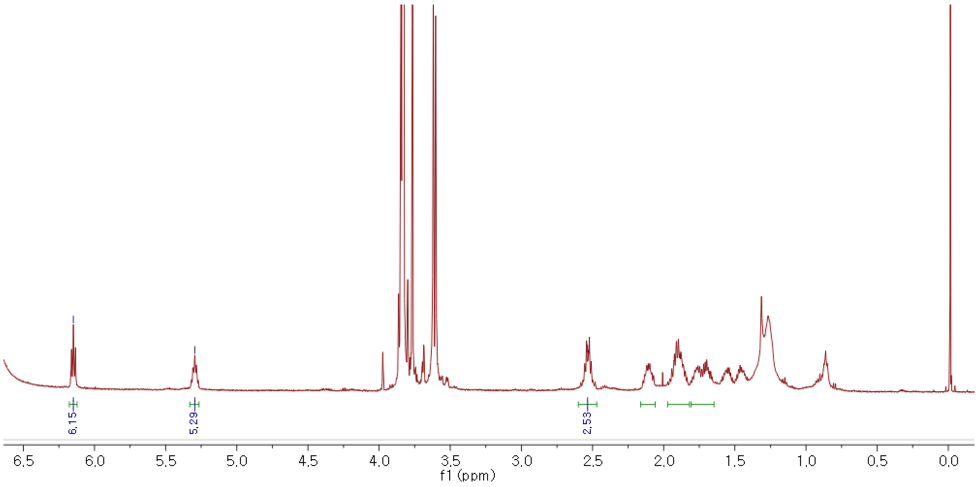

Figure S77. ¹H-NMR (pyridine-d_5, 500 MHz) spectrum of (*R*)-MTPA ester of **3b**.

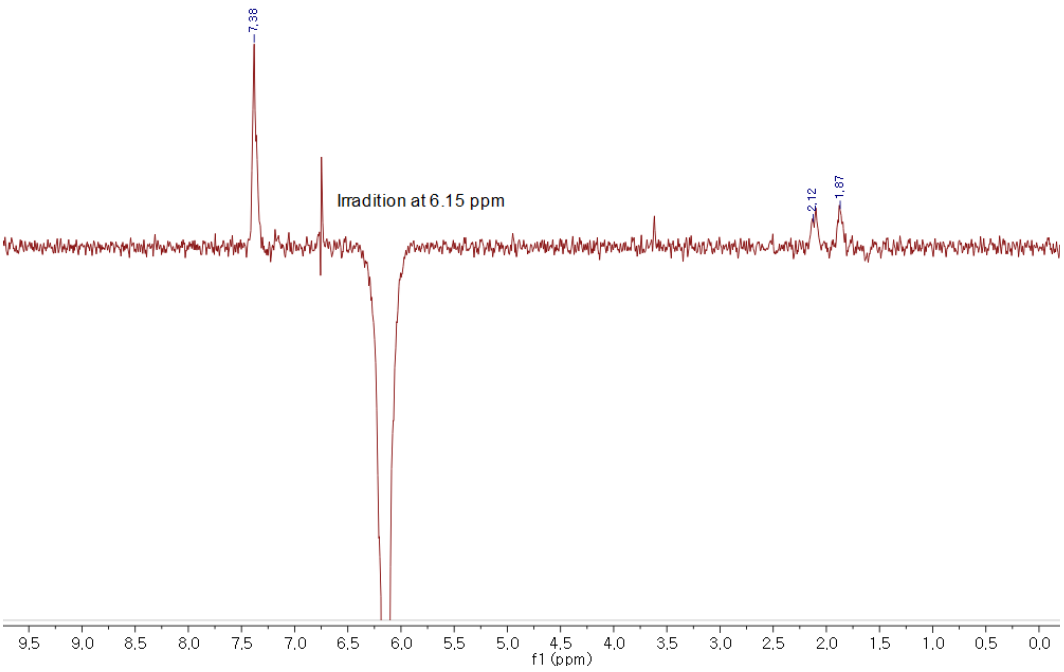

Figure S78. NOE spectrum of (*R*)-MTPA ester of **3b**.

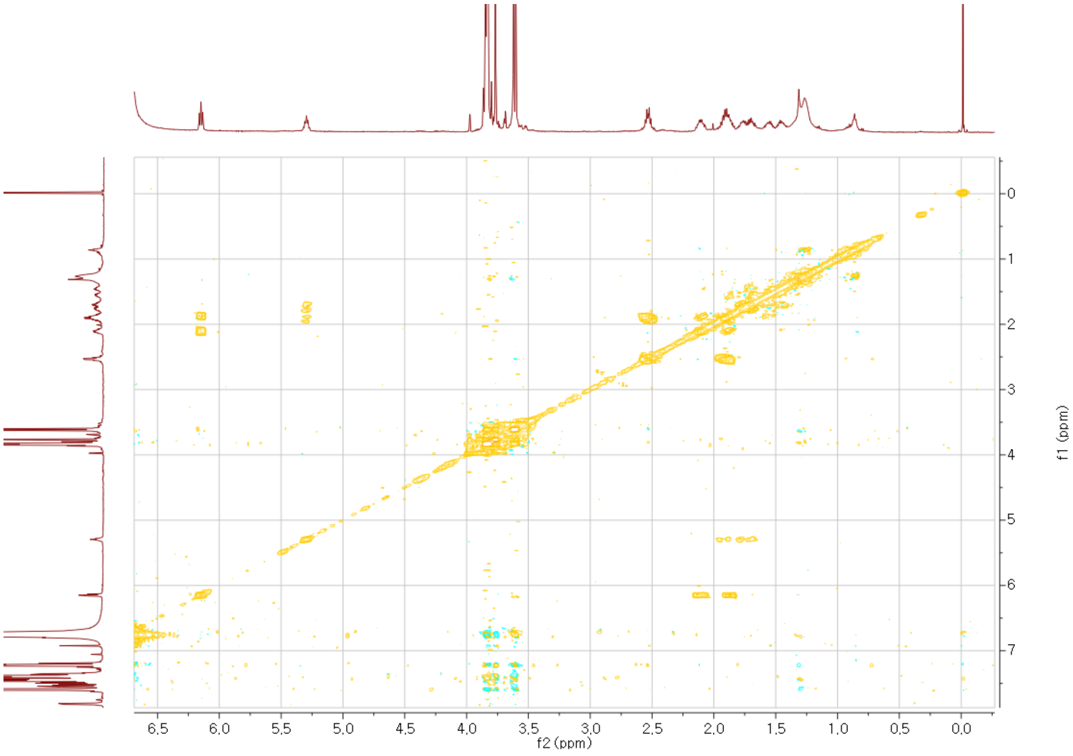

Figure S79. COSY spectrum of (*R*)-MTPA ester of **3b**.

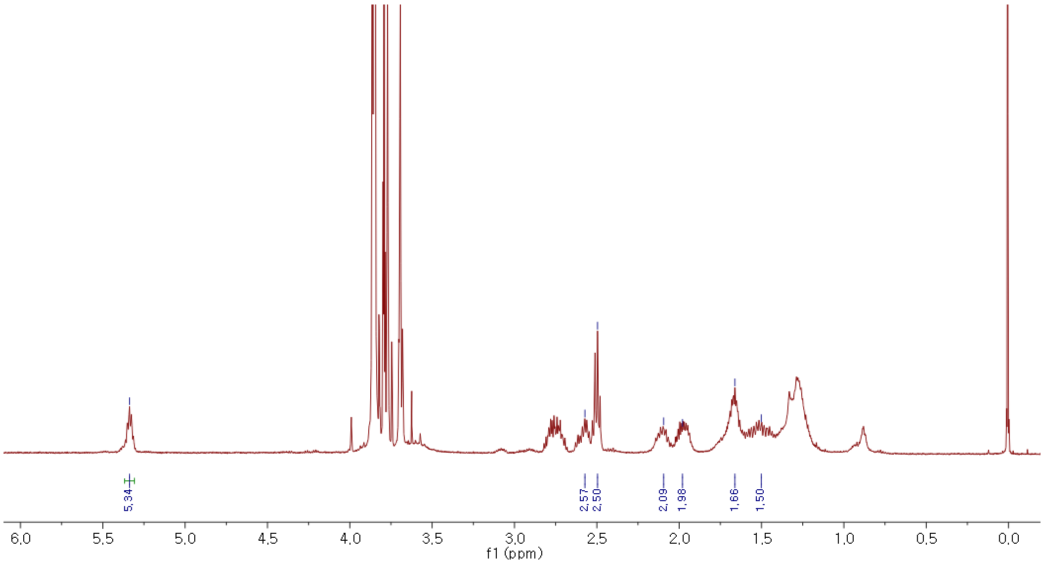

Figure S80. ^{1}H-NMR (pyridine-d_5, 500 MHz) spectrum of (*S*)-MTPA ester of **5**.

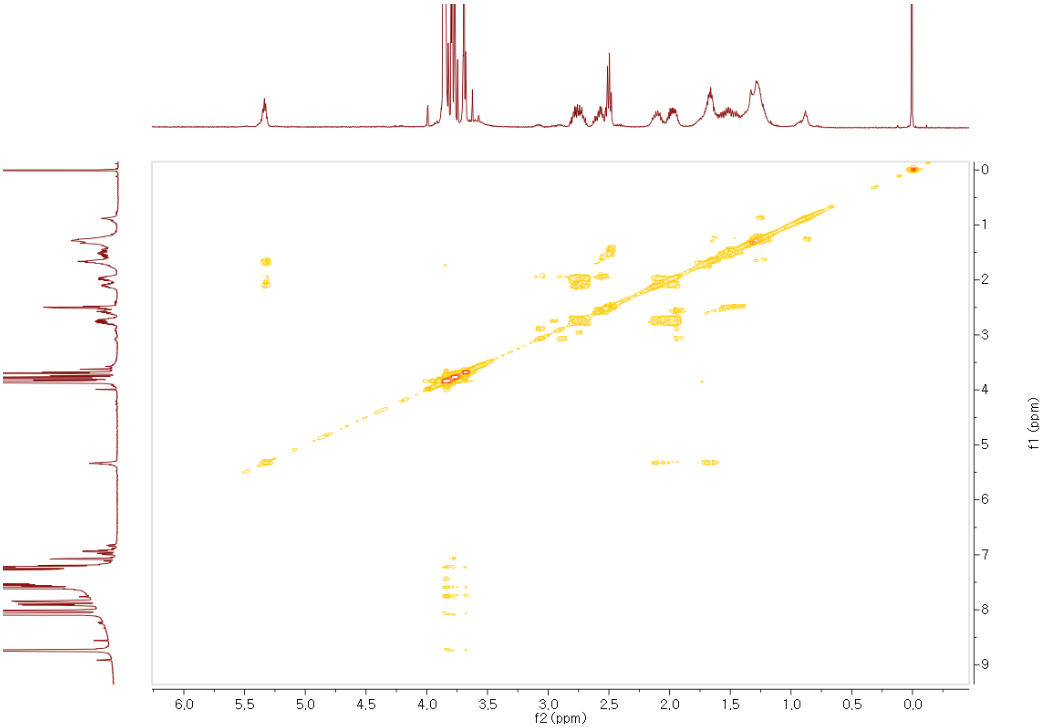

Figure S81. COSY spectrum of (*S*)-MTPA ester of **5**.

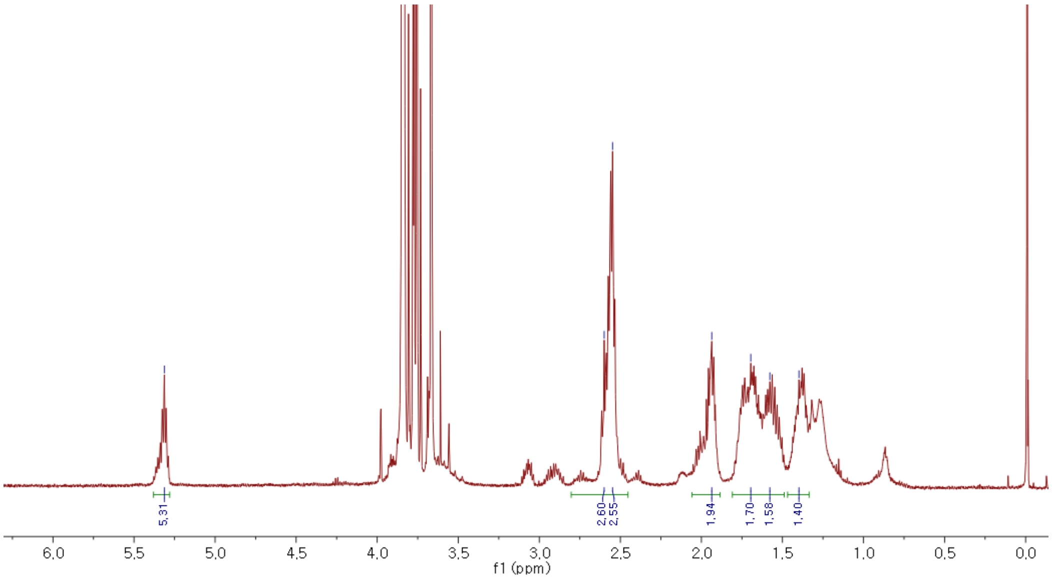

Figure S82. ^{1}H-NMR (pyridine-d_5, 500 MHz) spectrum of (*R*)-MTPA ester of **5**.

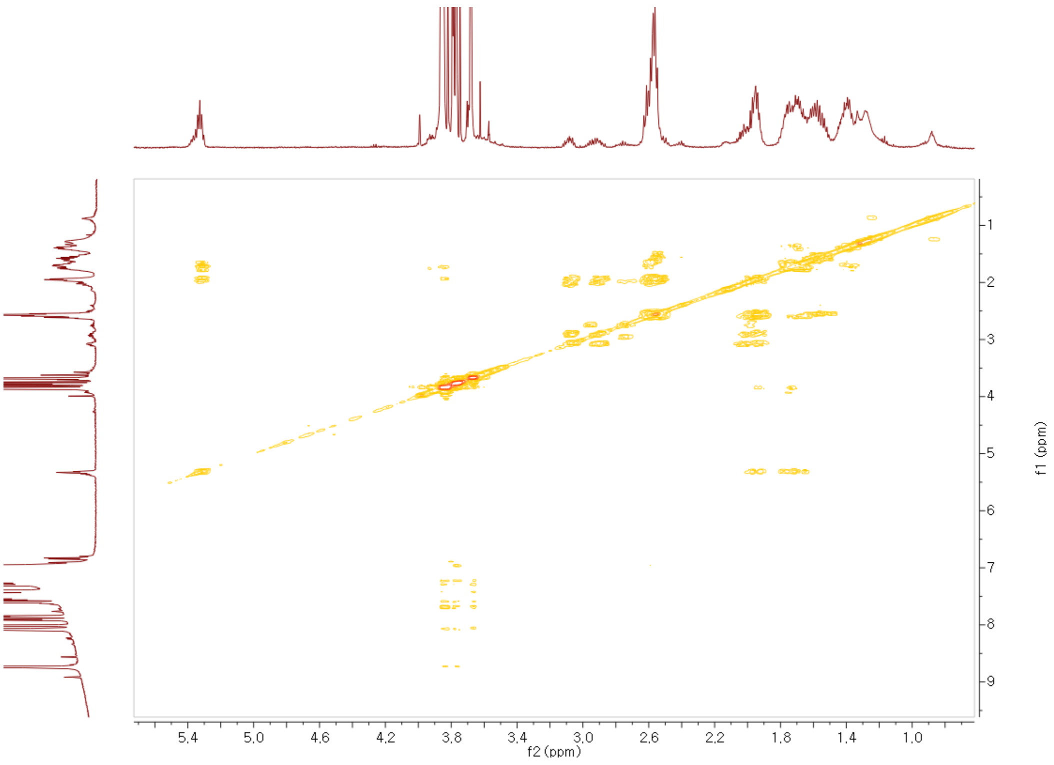

Figure S83. COSY spectrum of (*R*)-MTPA ester of **5**.

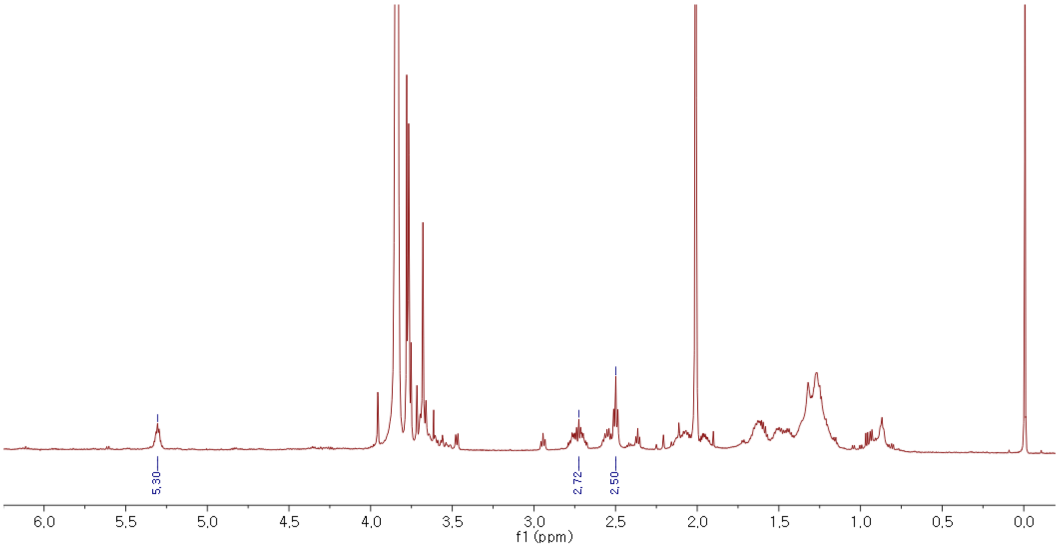

Figure S84. ^{1}H-NMR (pyridine-d_5, 500 MHz) spectrum of (*S*)-MTPA ester of **6**.

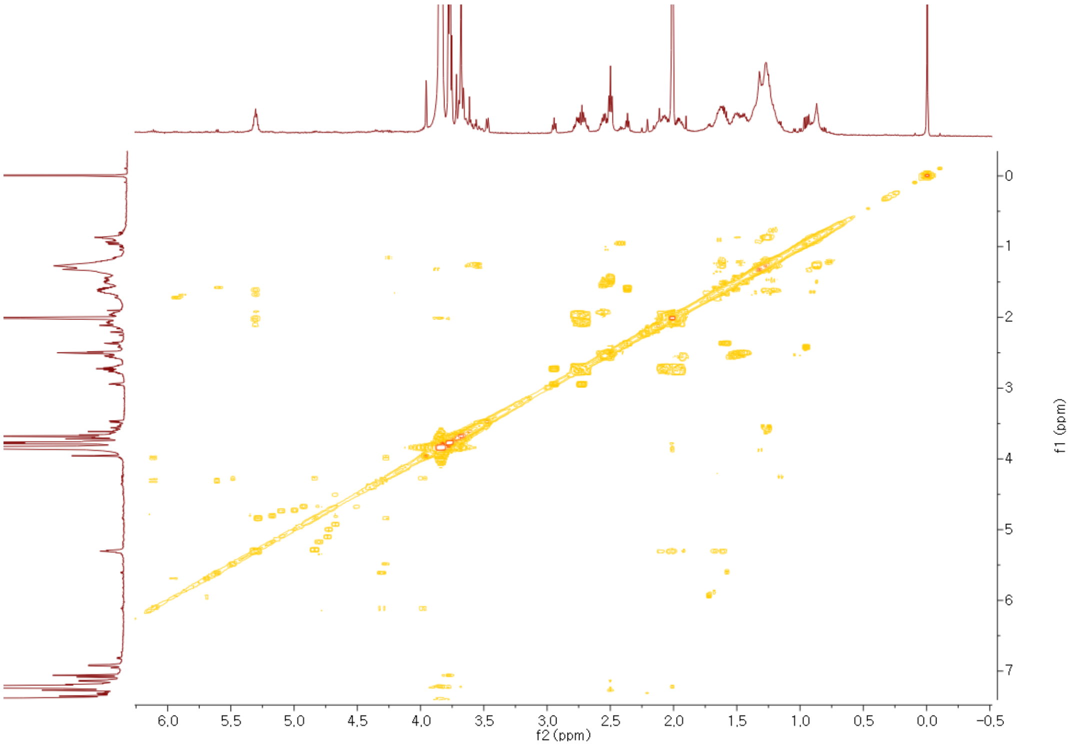

Figure S85. COSY spectrum of (*S*)-MTPA ester of **6**.

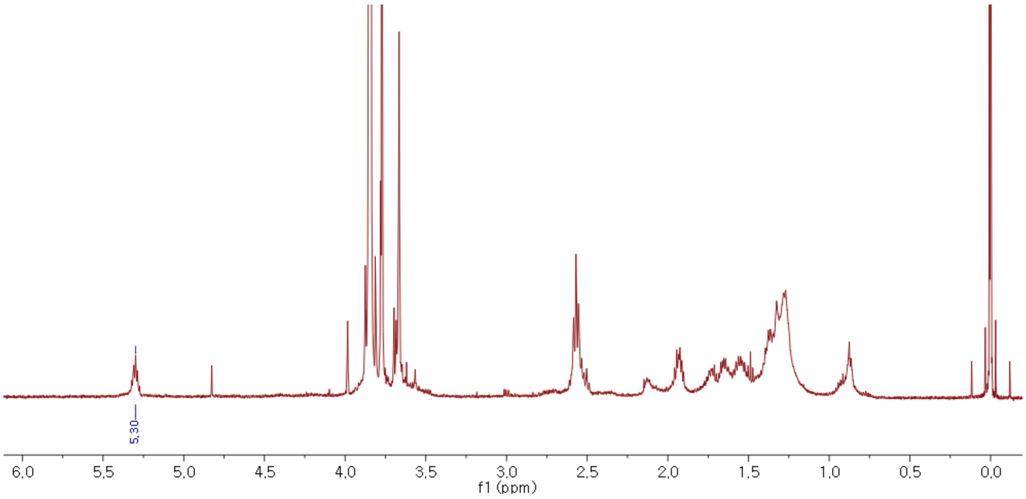

Figure S86. ^{1}H-NMR (pyridine-d_5, 500 MHz) spectrum of (*R*)-MTPA ester of **6**.

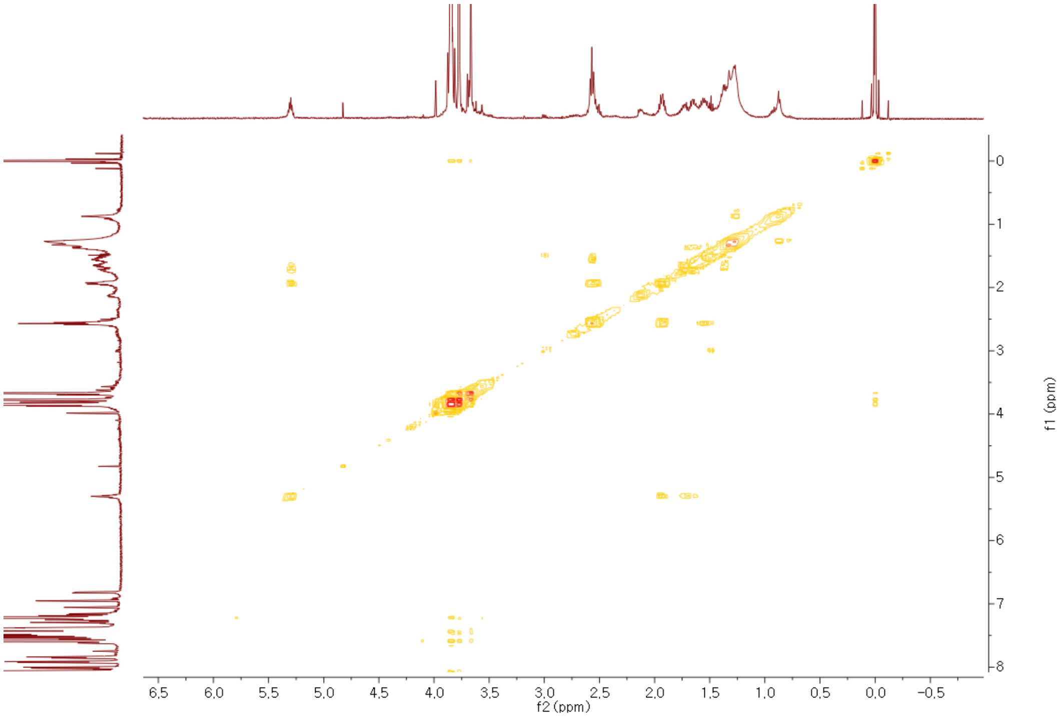

Figure S87. COSY spectrum of (*R*)-MTPA ester of **6**.

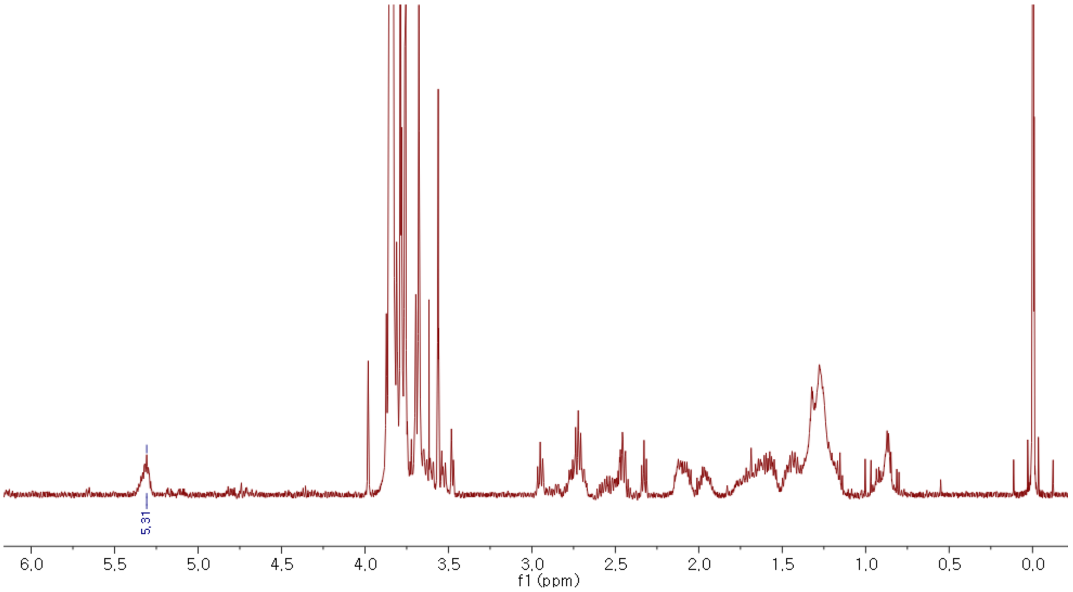

Figure S88. ^{1}H-NMR (pyridine-d_5, 500 MHz) spectrum of (*S*)-MTPA ester of **7**.

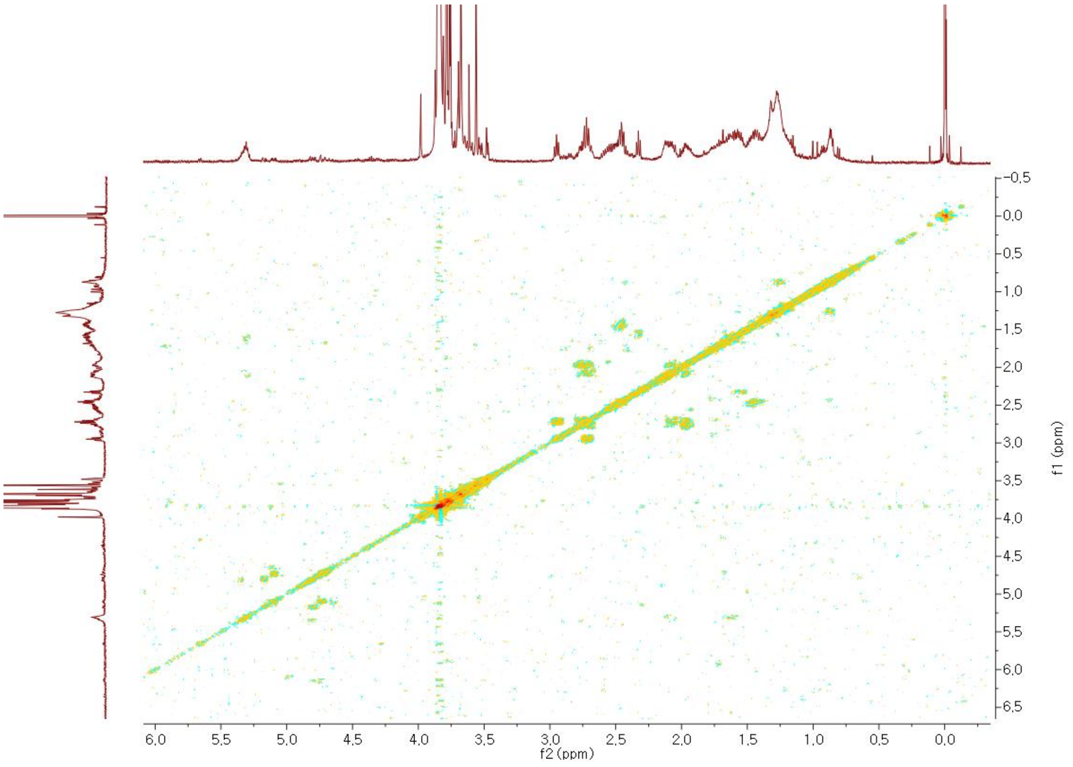

Figure S89. COSY spectrum of (*S*)-MTPA ester of **7**.

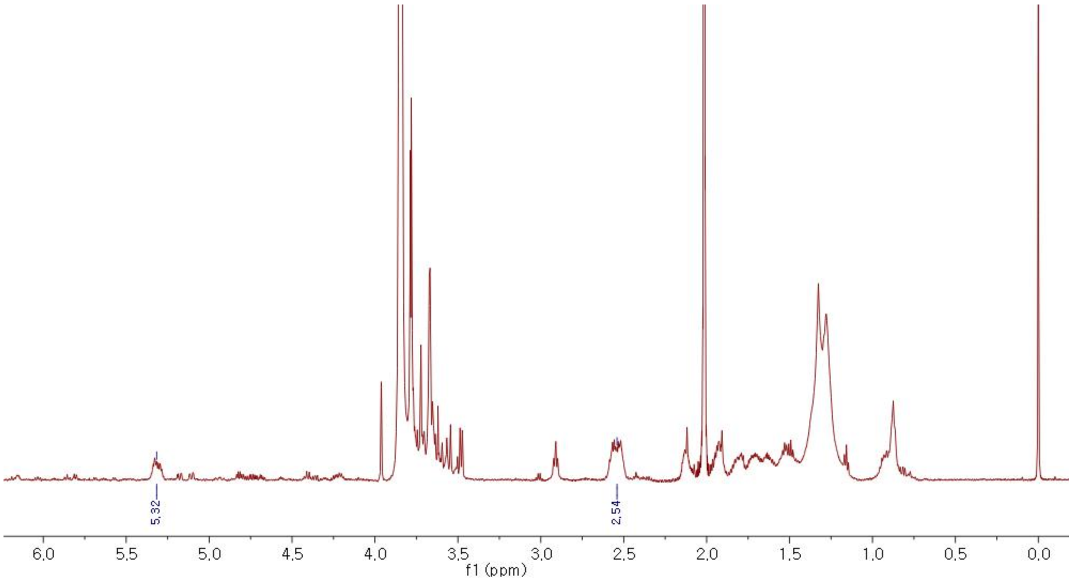

Figure S90. ^{1}H-NMR (pyridine-d_5, 500 MHz) spectrum of (*R*)-MTPA ester of **7**.

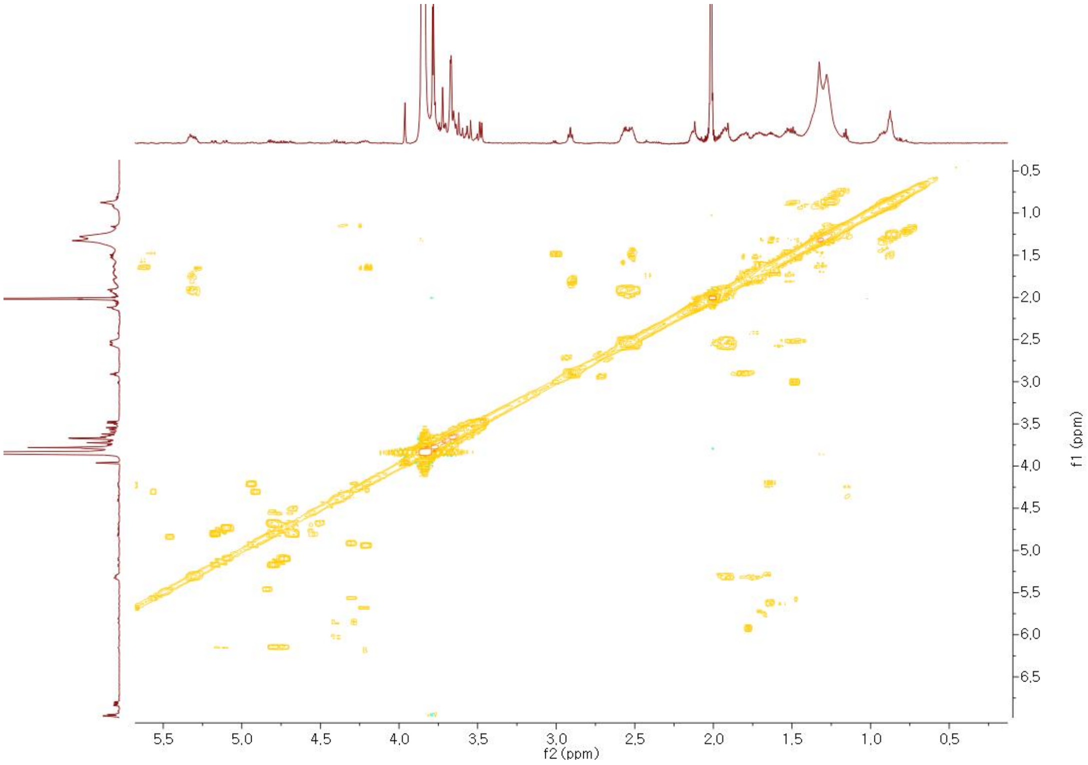

Figure S91. COSY spectrum of (*S*)-MTPA ester of **7**.
